# Taxonomic revision of *Physalis* in Mexico

**DOI:** 10.3389/fgene.2023.1080176

**Published:** 2023-04-14

**Authors:** Mahinda Martínez, Ofelia Vargas-Ponce, Pilar Zamora-Tavares

**Affiliations:** ^1^ Facultad de Ciencias Naturales, Universidad Autónoma de Querétaro, Querétaro, Mexico; ^2^ Laboratorio Nacional de Identificación y Caracterización Vegetal, Querétaro, Mexico; ^3^ Instituto de Botánica, departamento de Botánica y Zoología, Centro Universitario de Ciencias Biológicas y Agropecuarias, Universidad de Guadalajara, Guadalajara, Jalisco, Mexico; ^4^ Laboratorio Nacional de Identificación y Caracterización Vegetal, Guadalajara, Mexico

**Keywords:** revision, artificial keys, conservation, descriptions, distribution, uses

## Abstract

*Physalis* (Solanaceae, Solanoideae) is an American genus of ca. 90 species, with its diversity centered on Mexico. We recognize 61 species within the country, for which we provide a generic morphological description, an artificial key to determine species, and brief descriptions. We include distributions, habitats, diagnostic characters, phenology, and uses. Distribution maps and field photographs are also provided. We include conservation status as evaluated by the IUCN.

## 1 Introduction

*Physalis* (Solanoideae, Solanaceae) has been the subject of a large amount of research to determine its generic limits (Whitson and Manos, 2005; Zamora-Tavares et al., 2016), to address taxonomic problems (Pretz and Deanna, 2020), and describe cryptic species (Pyne et al., 2019). The plants are of economic interest because of their fleshy edible fruits, some of which are cultivated [e.g., *P. philadelphica* Lam., Zamora-Tavares et al. (2015); *P. peruviana* L., Muniz et al. (2014); and *P. angulata* L., Vargas-Ponce et al. (2016)], and others which are gathered from their wild populations as food or medicine (Kindscher et al., 2012; Martínez et al., 2022). As currently circumscribed, *Physalis* is an American genus, with its diversity centered on the United States (28 species; Pretz and Deanna, 2020), Mexico (61 species; this work), Central America [22 species, two of which are endemic, and absent from Mexico; Knapp et al. (2005)], and the West Indies (current estimates are not available). Two species (*P. viscosa* L. and *P. subilsiana* Toledo) are restricted to South America. However, assigning names to herbarium specimens remains complicated because many of the taxonomically important characters (e.g., calyx shape and anther color) are lost during herborization, and good field notes are usually lacking. In addition, many Mexican species were described by Waterfall (1967) based on single collections, making it difficult to assess their validity and morphological variation. Some of the Mexican species ignored in floristic works [e.g., *P. purpurea* Wiggins and *P. vestita* Waterf. by Martínez et al. (2017)] have turned out to be valid after new evidence has been gathered. Our study aims to provide a key, short descriptions, and conservation status for the species known to occur in Mexico. We include field photographs, diagnostic characters, and distribution maps.

## 2 Methodology

We reviewed plant material deposited in Mexican (ANSM, CIIDIR, ENCB, IBUG, IEB, MEXU, QMEX, XAL) and United States (TEX-LL, MO, SD) herbaria (herbarium codes follow Thiers, 2016) and confirmed or corrected previous identifications. Extensive collections have been made for most Mexican states, with emphasis on the north, west, and center. Specimens were georeferenced whenever possible and included in a dataset. The conservation status of most Mexican species was evaluated in 2017 by Vargas and Martínez using the AOO and EOO methods based on our distribution dataset, applying the IUCN criteria (Goettsch et al., 2021). The status of each species is available on the IUCN website (https://www.iucnredlist.org/search?query=physalis&searchType=species; IUCN, 2022). Distributions outside Mexico were also taken into consideration.

Type material designated in the protolog was located directly at the various herbaria. Small herbaria were searched with TORCH. We include either the catalog or the barcode number. Specimens not located are noted as such. Only *P. campechiana* and *P. lagascae* remain without a type, since suitable specimens are probably deposited in European herbaria to which we have no access.

To elaborate the distribution maps, we included the specimens from the afore mentioned herbaria and specimens available as images, such as via SEINET and MEXU, only if the specimen could be confidently determined and the geographical coordinates either were given in the label or could be unambiguously assigned.

## 3 Morphology

### 3.1 Habit

Plants are frequently annuals that complete their life cycle within a year [e.g., *P. solanacea* (Schltdl.) Axelius or *P. patula* Miller] and appear after the summer rains in June or July. Perennial plants are rhizomatous types that die back as the unfavorable season begins (e.g., *P. cinerascens* (Dunal) Hitchc. or *P. vestita*); however, in mesic species, the whole plant might be present throughout the year (e.g., *P. volubilis* Waterf.). Finally, a few species are either shrubs [e.g., *P. campechiana* L. and *P. melanocystis* (B. L. Rob.) Bitter] or weak suffrutex that lean on other plants (e.g., *P. coztomatl* Dunal and *P. lignescens* Waterf.). The species are mostly erect and can grow up to 2–3 m high (e.g., *P. latiphysa* Waterf.), but a few (*P. gracilis* Miers, *P. queretaroensis* M. Martínez & L. Hernández, and *P. volubilis*) are decumbent and form long stolons that root at the nodes. Stems can branch profusely, especially in perennial plants, as in the case of *P. crassifolia* Beth., or remain a single stem or one with a few branches. Stems are mostly terete, with the exception of *P. angulata*, which has angled stems.

### 3.2 Leaves

The leaves are alternate, but in many cases the upper leaves are geminate, and on some occasions, they are geminate throughout the plant. Geminate leaves usually have a large leaf accompanied by a smaller one, which is frequently half the size but has the same shape. The leaves can be sessile or petiolate, and the base is frequently oblique.

### 3.3 Vestiture

Trichomes can be simple eglandular, glandular, or branched. Glandular trichomes lend a fetid smell to the plant (e.g., *P. nicandroides* Schltdl. and *P. glutinosa* Schltdl.) or gather debris and insects, giving the plant a dirty appearance (*P. sordida* Fernald). Many specimens are poorly preserved because pressing such plants is difficult. Although most species have some form of vestiture, a few are glabrous (*P. glabra* Benth.) or puberulent, and the trichomes cannot be detected by the naked eye. Several species are glabrescent, and trichomes are only present in the younger parts. A detailed description of hair types is provided by Seithe and Sullivan (1990).

### 3.4 Flowers

**FIGURE 1:**
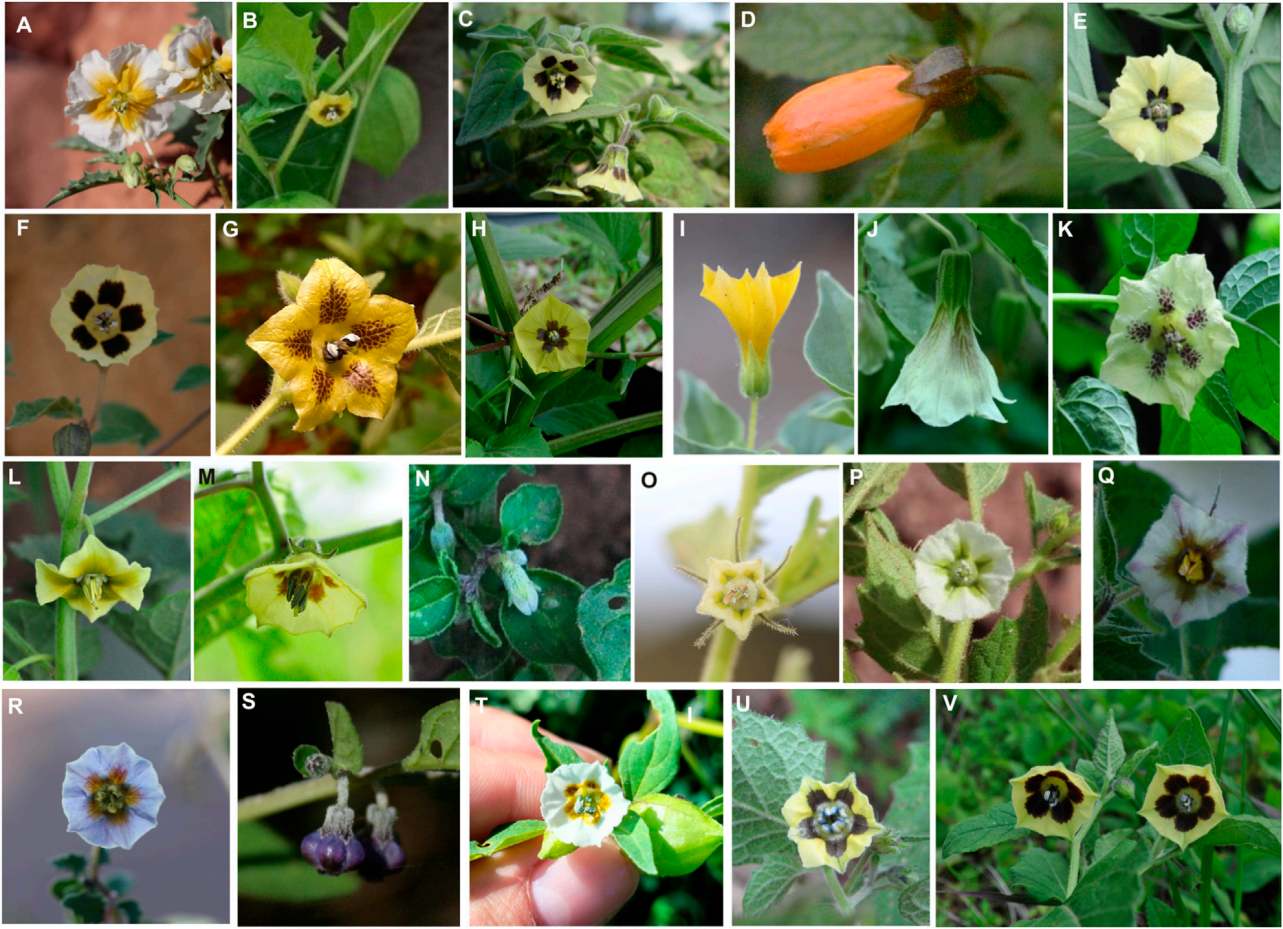
Corolla diversity of *Physalis* spp. **(A)** *P. acutifolia*, **(B)** *P. angulata*, **(C)** *P. angustiphysa*, **(D)** *P. campanula*, **(E)** *P. cinerascens*, **(F)** *P. chenopodiifolia*, **(G)** *P. coztomatl*, **(H)** *P. cordata*, **(I)** *P. crassifolia* var. *infundibularis*, **(J)** *P. glutinosa*, **(K)** *P. greenmanii*, **(L)** *P. hederifolia*, **(M)** *P. latiphysa*, **(N)** *P. microcarpa*, **(O)** *P. nicandroides*, **(P)** *P. patula*, **(Q)** *P. pruinosa*, **(R)** *P. purpurea*, **(S)** *P. solanacea*, **(T)** *P. sulphurea*, **(U)** *P. tamayoi*, **(V)** *P. waterfallii.*

Corolla shape is rotate to campanulate, 5-angled, or 5-lobed (Figures 1A–C, E–I, O–R, T–V). Tubular corollas occur only in *P. campanula* Standl. & Steyerm. (Figures 1D, 4E) and *P. glutinosa* Dunal (Figures 1J, 6C), and urceolate corolla occurs only in *P. solanacea* (Figures 1S, 14A). The color varies from almost white (Figures 1A, N, P, Q, T) to orange (Figures 1D, 4E). However, most species have yellow corollas (Figures 1B, C, E–I, L, M, U, V). Only *P. solanacea* (Figures 1S, 14A) and *P. purpurea* (Figures 1R, 12H) have purple corollas. Maculae are present in many species, varying in shape and color; a few species have compound macula [e.g., *P. coztomatl* Dunal (Figures 1G, 6A) or *P. greenmanii* Waterf (Figure 1K)], and other species lack maculations (Figures 1I, N). A dense mat of white trichomes is present at the corolla throat or at the filament insertion in most species. Martínez (1998) presents diagrams of corolla shapes and maculations.

### 3.5 Inflorescence

Most of the species have a solitary pendulous axillary flower. The exceptions are *P. aggregata* Waterf. (Figure 4C), *P. angustior* Waterf., *P. campechiana*, and *P. melanocystis* (Figure 9H), which have fascicles of two or more flowers. Flower peduncles are usually elongated in fruit.

### 3.6 Fruits

**FIGURE 2:**
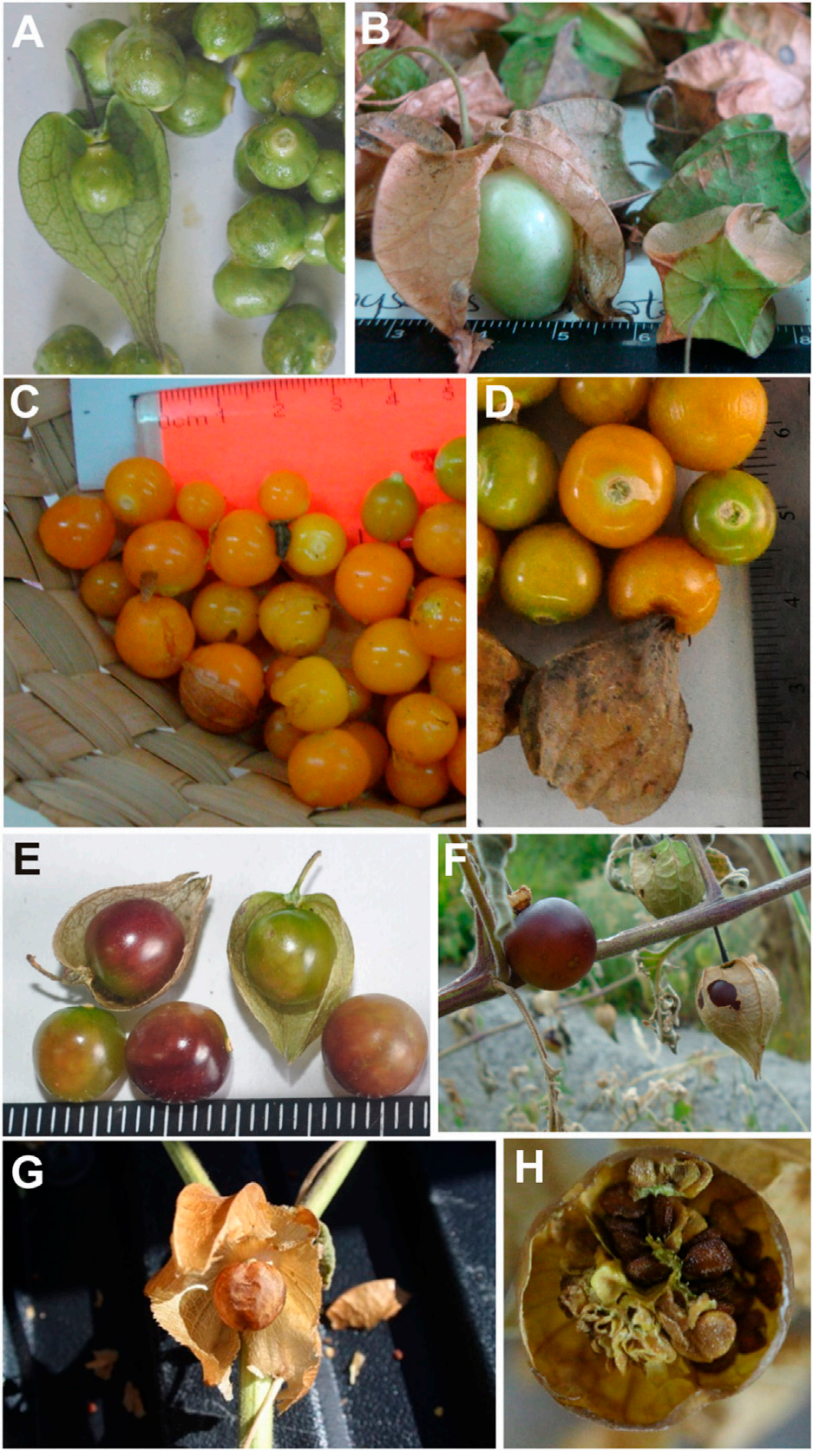
Diversity of fruits of *Physalis* spp. **(A)** *P. aggregata*, **(B)** *P. cordata*, **(C)** *P. gracilis*, **(D)** *P. grisea*, **(E)** *P. microcarpa*, **(F)** *P. chenopodiifolia*, **(G)** *P. nicandroides*; **(H)** cross-section of *P. nicandroides* fruit showing the dry pericarp.

The fruit of *Physalis* is always a two-carpellate berry that is usually juicy. These fruits mature in different colors: they can be green (Figures 2A, B), yellow (Figures 2C, D), orange, purple (Figure 2E), or almost black (Figure 2F). A few species present a dry pericarp [e.g., *P. nicandroides* (Figures 2G, H), *P. patula* Miller, and *P. latiphysa*], and the fruits are brown when mature. Flavors are acidic, bitter, or sweet, and in a few species, the fruit is strongly glutinous with a fetid odor. Seeds are commonly abundant, only *P. glutinosa* (Figure 6C) and *P. solanacea* (Figure 14A) always have a small number (5–10). Fruits formed at the end of the growing season may also contain fewer seeds.

**FIGURE 3:**
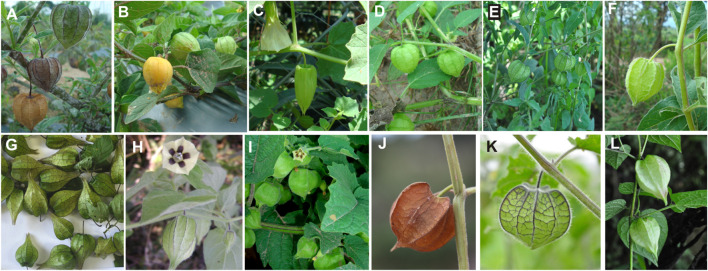
Fruiting calyces of *Physalis* spp. **(A)** *P. acutifolia*, **(B)** *P. gracilis*, **(C)** *P. glutinosa*, **(D)** *P. lagascae*, **(E)** *P. hastatula*, **(F)** *P. waterfallii*, **(G)** *P. aggregata*, **(H)** *P. angustiphysa*, **(I)** *P. grisea*, **(J)** *P. nicandroides*, **(K)** *P. pubescens*, **(L)** *P. porrecta.*

Fruits are covered by an accrescent calyx, which takes different shapes (see Martínez, 1998 for a diagram). Sometimes the calyx is evidently 10-costate (Figures 3A–F), and in other cases it is strongly 5-angled (Figures 3G–L). However, a few species have an intermediate position in which some of the nerves are more prominent than others. The insertion of the peduncle can be strongly invaginated, but invagination might be absent in other species. The fruiting calyx is green in immature fruits and becomes yellow (Figure 3A, B) or brown (Figure 3J) when the fruits mature. Only *P. campechiana* and *P. melanocystis* (Figure 9H) have purple or almost black fruiting calyces. The tip of the fruiting calyx lobes is constricted, but it remains open in *P. purpurea* (Figure 12H).

### 3.7 Seeds

Seeds of all the species of *Physalis* have a reniform shape and are compressed. The color varies from yellow to dark brown, and the size varies from 1 to 3 mm in Mexican species. The testa is described as foveolate. The etched seeds have undulated anticlinal walls and holes at the bottom, and have been described in some detail by Axelius (1992).

## 4 Distribution

Of the 61 species that we recognize as part of the Mexican flora, ten have a wide distribution that includes the United States, Central and South America, and the West Indies [*P. angulata*, *P. cordata* Miller, *P. grisea* (Waterf.) M. Martínez, *P. ignota* Britton, *P. lagascae* Roem. & Schult., *P. microcarpa* Urb. & Ekman, *P. philadelphica*, *P. pruinosa*, *P. pubescens* L., and *P. solanacea*]. Seven species are distributed in Mexico and the southern portion of the United States [*P. acutifolia* (Miers) Sandwith, *P. caudella* Standl., *P. cinerascens*, *P. crassifolia*, *P. hederifolia* A. Gray, *P. latiphysa*, and *P. spathulifolia* (Torr.) B. L. Turner]. Eight grow in Mexico and Mesoamerica (*P. angustiphysa* Waterf., *P. campanula*, *P. campechiana*, *P. lassa* Standl. & Steyerm., *P. leptophylla* B.L. Rob. & Greenm., *P. melanocystis*, *P. nicandroides*, and *P. porrecta* Waterf.). The remainder (36 species) are endemic to Mexico, 18 of which are micro-endemic to one state (e.g., *P. greenmanii*, *P. glabra*, *P. mcvaughii* Waterf., *P. minimaculata* Waterf., *P. purpurea*, and *P. vestita*), to a few locations (*P. hintonii* Waterf. and *P. pennellii* Waterf.), or to a mountain range (*P. angustior* Waterf., *P. hunzikeriana* M. Martínez, *P. lignescens* Waterf., *P. longiloba* O. Vargas, M. Martínez & Dávila, and *P. longipedicellata* Waterf.). See Table 1 for species distribution and conservation status in Mexico.

**TABLE 1:** Species distribution and conservation status of *Physalis* in Mexico.

Species	Conservation status	Distribution in Mexico
*P. acutifolia*	LC	B.C., B.C.S., Chih., Chis., Col., Camp., Jal., Mich., Nay., Sin., Son., Zac.
*P. aggregata*	VU	Jal., Oax.
*P. ampla*	LC	Col., Chih., Dgo., Gto., Jal., Mich., Nay., Oax., Qro., Sin., Son.
*P. angulata*	LC	Ags., B.C.S., Camp., Chis., Chih., Col., Dgo., Gto., Jal., Mich., Nay., N.L., Oax., Qro., Tab., Tamps., Sin., Son., Ver., Zac.
*P. angustior*	DD	Mor.
*P. angustiphysa*	LC	Chis., Gto., Jal., Mich., Nay., Son., Tamps.
*P. campanula*	NT	Chis., Hgo., Oax., Ver.
*P. campechiana*	LC	Camp., Chis., Oax., Q. Roo, Tab., Tam., Ver., Yuc.
*P. caudella*	LC	Chih., Coah., Dgo., Hgo., Gto., N.L., Qro., S.L.P., Son., Tamps., Zac.
*P. chenopodiifolia*	LC	Ags., Chih., Cd. Mex., Dgo., Edo. Mex., Gro., Gto., Hgo., Jal., Mich., N.L., Pue., Qro., S.L.P., Tlax., Zac.
*P. cinerascens*	LC	Ags., Camp., Chis., Coah., Edo. Mex., Gto., Hgo., Jal., Mich., N.L., Oax., Pue., Qro., S.L.P., Tamps., Ver., Yuc., Zac.
*P. cordata*	LC	Camp., Chis., Col., Gro., Jal., Mich., Nay., Oax., Sin., Tab., Ver., Yuc.
*P. coztomatl*	LC	Cd. Mex., Edo. Mex., Gro., Hgo., Jal., Mich., Mor., Nay., Oax., Pue., Qro., Tlax., Ver.
*P. crassifolia*	LC	B.C., B.C.S., Sin., Son.
*P. glabra*	LC	B.C.S.
*P. glutinosa*	LC	Ags., Chih., Cd. Mex., Dgo., Gto., Hgo., Jal., Zac.
*P. gracilis*	LC	Camp., Chis., Hgo., Jal., Mor., Nay., Oax., Pue., Qro., Q. Roo, S.L.P., Tab., Tamps., Ver.
*P. greenmanii*	EN	Ver.
*P. grisea*	LC	Jal., Mich.
*P. hastatula*	EN	Ags., Gto., Jal., Zac.
*P. hederifolia*	LC	Chih., Coah., Dgo., Gro., Jal., N.L., Oax., Qro., S.L.P., Son., Tamps., Zac.
*P. hintonii*	LC	Edo. Mex., Mich., Ver.
*P. hunzikeriana*	DD	N.L.
*P. ignota*	LC	Chis., Oax.
*P. lagascae*	LC	Ags., Camp., Chis., Chih., Col., Dgo., Edo. Mex., Gto., Gro., Jal., Mich., Mor., Nay., N.L., Oax., Pue., Qro., Q. Roo, Sin., Tab., Ver., Yuc., Zac.
*P. lassa*	LC	Chis., Col.
*P. latecorollata*	DD	Oax.
*P. latiphysa*	LC	Gto., Mich., Son.
*P. leptophylla*	LC	B.C.S, Chih., Col., Dgo, Edo. Mex., Gro., Jal., Mich., Nay., Oax., Sin., Son.
*P. lignescens*	EN	Jal.
*P. longiloba*	LC	Jal.
*P. longipedicellata*	LC	Jal.
*P. mcvaughii*	NT	Jal.
*P. melanocystis*	LC	Camp., Chis., Col., Gro., Hgo., Jal., Oax., Qro., S.L.P., Tab., Tamps., Ver.
*P. microcarpa*	LC	Chis., Edo. Mex., Jal., Son.
*P. minimaculata*	VU	Mich.
*P. minuta*	LC	Col., Chis., Gro., Jal., Nay., Oax.
*P. nicandroides*	LC	Ags., B.C.S., Chis., Col., Dgo., Edo. Mex., Gto., Gro., Hgo., Jal., Mich., N.L., Mor., Nay., Oax., Pue., Qro., S.L.P., Sin., Son., Tamps., Ver., Yuc., Zac.
*P. orizabae*	LC	Ags., Cd. Mex., Chih., Dgo., Edo. Mex., Gto., Gro., Hgo., Jal., Mich., Mor., Nay., N.L., Oax., Pue., Qro., S.L.P., Ver., Zac.
*P. parvianthera*	DD	Mor.
*P. patula*	LC	Ags., Chih., Col., Cd. Mex., Dgo., Edo. Mex., Gto., Hgo., Jal., Mich., Oax., Pue., Qro., Sin., Tlax., Ver., Zac.
*P. pennellii*	NT	S.L.P.
*P. philadelphica*	LC	Present in all the Mexican states except Camp., Tlax., and Yuc.
*P. porrecta*	DD	Chis., Gro., Oax.
*P. pringlei*	LC	Cd. Mex., Dgo., Oax.
*P. pruinosa*	LC	Chis., Chih., Edo. Mex., Col., Gro., Jal., Mor., Nay., Oax., Qro., Sin., Son., Tamps., Ver., Yuc., Zac.
*P. pubescens*	LC	BCS., Camp., Chis., Chih., Coah., Col., Dgo., Gro., Hgo., Jal., Mich., Nay., NL., Oax., Pue., Qro., Q. Roo, Sin., Son., Tab., Tamps., Ver., Yuc.
*P. purpurea*	DD	Son.
*P. queretaroensis*	LC	Qro.
*P. rydbergii*	DD	Gto., Mich, Qro., Sin.
*P. sancti-josephi*	DD	Hgo., Jal, Nay., Qro., S.L.P.
*P. solanacea*	LC	Chih., Coah., Dgo., Gto., Hgo., Jal., Mich., N.L., Oax., Pue., Qro., S.L.P., Tamps., Ver., Zac.
*P. sordida*	LC	Coah., Dgo., Edo. Mex., Gto., Hgo., Jal., Mich., NL., Oax., Pue., Qro., S.L.P., Tam., Zac.
*P. spathulifolia*	LC	Tamps
*P. subrepens*	LC	Edo. Mex., Hgo., Jal., Ver.
*P. sulphurea*	LC	Cd. Mex., Dgo., Gto., Jal., Mich., Qro.
*P. tamayoi*	NT	Jal.
*P. tehuacanensis*	CR	Pue.
*P. vestita*	Probably VU	Sin.
*P. volubilis*	LC	Mich., Jal.
*P. waterfallii*	LC	Jal., Mich., Qro.

## 5 Economic importance, current or potential

In Mexico, sixteen wild species are locally used as food and for medicinal purposes (Vargas-Ponce et al., 2016; Martínez et al., 2022). The edible fruits of some *Physalis* species are nutritionally important (Puente et al., 2011; Zamora-Tavares et al., 2015; Valdivia-Mares et al., 2016). *Physalis acutifolia*, *P. angulata*, *P. chenopodifolia* Lam., *P. cinerascens*, *P. cordata*, *P. coztomatl*, *P. gracilis*, *P. philadelphica*, and *P. pubescens* are harvested from their natural populations for consumption (Santiaguillo Hernández and Blas, 2009; Vargas-Ponce et al., 2011; Martínez et al., 2022). The flavor of the fruits can be sweet or sour, and they are consumed fresh or in jams, sauces, and other dishes (Santiaguillo Hernández and Blas, 2009; Vargas-Ponce et al., 2016). In particular, *P. philadelphica* is cultivated extensively in Mexico (Peña-Lomelí et al., 2001; Zamora-Tavares et al., 2015) and, to a lesser extent, in Guatemala and Nicaragua (López et al., 2009; Samuels, 2009). Mexico has a *per capita* consumption of *P. philadelphica* of 5.3 kg; this is the fifth horticultural crop in Mexico (SIAP 2019). It is also exported to the United States of America, the United Arab Emirates, the United Kingdom, France, Poland, Saudi Arabia, Canada, Switzerland, and Jamaica (SIAP, 2019). In recent years, *P. angulata* has gained importance at the regional scale (Vargas-Ponce et al., 2016). The cultural tradition of harvesting, the preference for small, acid-flavored fruits, and the high price and high yield make *P. angulata* a species with the potential to be cultivated extensively (Morales-Saavedra et al., 2019).

The roots, leaves, stems, fruits, and fruiting calyces of several species (*P. philadelphica*, *P. angulata*, *P. chenopodifolia*, *P. cinerascens*, *P. pubescens*, and *P. coztomatl*) are used by local populations as remedies. They are used as diuretics and against headaches, stomach pains, diarrhea, skin rash, and diabetes (Martínez et al., 2022). The various species of *Physalis* also have a reputation as fodder for cattle.

## 6 Taxonomic history

The taxonomic history of *Physalis* is complex due to controversies regarding the diagnostic characters traditionally used for its classification. From Linnaeus (1753) to Rydberg (1896), the characters commonly used to subdivide the genus included habit, flower arrangement, shape of the fruiting calyx, and pubescence (Martínez 1998). Waterfall (1967) monographed the vast majority of species. His study was followed by several nomenclature changes and the discovery of new species. Martínez (1999) proposed an infrageneric classification based on habit, flower arrangement, flower and fruit morphology, trichome micromorphology, habitat, and geographical distribution. According to Martínez (1999), *Physalis* has four subgenera: 1) *Physalis* L., 2) *Physalodendron* (G. Don.) M. Martínez, 3) *Quincula* (Raf.) M. Martínez, and 4) *Rydbergis* Hendrych. The latter includes nine sections: *Angulatae* (Rydb.) Menzel, *Campanulae* M. Martínez, *Carpenteri* M. Martínez, *Coztomatl* M. Martínez, *Epeteiorhiza* G. Don, *Lanceolata* (Rydb.) Menzel, *Rydbergae* M. Martínez, *Tehuacanae* M. Martínez, and *Viscose* (Rydb.) Menzel. Regional floristic studies have further advanced knowledge of *Physalis*. They have included the southeastern United States (Sullivan 2004), Mesoamerica (Knapp et al., 2005), and Mexico (Martínez et al., 2017; Martínez 2020). The molecular evidence does not support the proposal of Martínez (1999), and this concept of the genus *Physalis* is paraphyletic (Zamora-Tavares et al., 2016; Deanna et al., 2018). However, the *Physalis* subgenus *Rydbergis* is monophyletic (Zamora-Tavares et al., 2016; Deanna et al., 2018) and includes 75 American native species (Martínez 1999). The paraphyly of *Physalis* has been partially resolved by segregating species as for *Physalis alkekengi* L., as *Alkekengi officinarum* Moench (Whitson, 2011) and *P. carpenteri* Riddell as *Calliphysalis carpenteri* (Riddell) M. Whitson (Whitson, 2012). This implied the proposal of *Physalis pubescens* L. as the new nomenclatural type for the genus (Whitson, 2011). *Physalis lobata* Raf., which was recognized by Martínez (1999) as a monotypic subgenus, was rehabilitated as the genus *Quincula* Raf. by Barboza (2000). However, further taxonomic changes are needed to make *Physalis* a monophyletic group.

## 7 Generic description and species key

*Physalis* L., sp. Pl. 1:182, 1753. Conserved type: *Physalis pubescens* L. (Whitson 2011).

Shrubs, suffrutex, annual, or perennial herbs forming rhizomes; pubescence of simple eglandular, glandular, or branching trichomes, frequently a mixture of two types; leaves petiolate, seldomly sessile, alternate but frequently geminate, especially on the upper branches, blades oblong, elliptic, ovate, or lanceolate, less frequently rhombic, trulate, or linear, margin entire or dentate; flowers solitary or aggregated in few-flowered axillary fascicles, pedunculate; calyx campanulate to tubular-campanulate, 5-lobed, lobes triangular to acuminate; flower buds plicate, corolla actinomorphic, rotate, campanulate, tubular-campanulate, or urceolate, mostly yellow but some species with purple, orange, or white petals, yellow flowers commonly with five simple or compound purple strongly contrasting maculations, or maculations greenish or brown, not strongly contrasting, sometimes maculations lacking, corolla throat usually pubescent at the insertion of filaments; filaments filiform or flattened; stamens 5, anthers dehiscing longitudinally, oblong, linear-oblong or sagittate, green, yellow, blue, blue-tinged, or purple; style filiform, stigma capitate or clavate; fruit a two-carpellate spheric berry, green, yellow, orange, purple, brown, or almost black at maturity, that is loosely and completely covered by the accrescent calyx; fruiting calyces 5-angled or 10-costate; seeds usually numerous, yellow or brown at maturity, laterally compressed, testa foveolate.

**Artificial key to the species of *Physalis* in Mexico**

1. Plants shrubby or suffrutescent. ……………………… **Key 1**
1. Plants annual or perennial, but not shrubby… … … ………2
  2. Plants with branched trichomes………………… …**Key 2**
  2. Plants with simple glandular or eglandular trichomes; or plants glabrous, but branched trichomes absent…… ……3
    3. Plants glabrous or minutely puberulent**…………Key 3**
    3. Plants variously pubescent, but not minutely puberulent………………… ……… ……… …… 4
    4. Plants with simple eglandular trichomes…………**Key 4**
    4. Plants with glandular trichomes, sometimes mixed with simple eglandular trichomes ……………………**Key 5**

**Key 1: Plants shrubby or suffrutescent**

1. Corolla tubular or campanulate, flowers solitary……………**2**
  2. Corolla tubular, limb not expanded, apex minutely 5-dentate, 1.5–2.0 cm long and 1 cm wide, orange-yellowish, immaculate, fruiting calyx glabrous………………………… ***P. campanula***
  2. Corolla campanulate, limb expanded, 2.5–3.5 cm long and 2.5–3–5 cm wide when fully expanded, yellow, with five large compound maculations, fruiting calyx pubescent…… ……………………………………………………… ***P. glutinosa***
1. Corolla rotate, 5-lobed or not, flowers solitary or in fascicles……………………………………………………3
  3. Flowers in fascicles, with (1) two to seven flowers per node, fruiting calyx purple to almost black at maturity……………4
    4. Plants with simple trichomes… … ……… ***P. melanocystis***
    4. Plants with dendritic trichomes……… . ***P. campechiana***
  3. Flowers solitary or rarely with a shortened internode that aggregates one to three flowers, fruiting calyx green at maturity………………………… ……………………5
    5. Internode shortened and then one to three flowers aggregated………………………… …… ***P. angustior***
    5. Internode long, flowers solitary…………… …………6
      6. Fruiting calyx five-angled………………………… … 7
        7. Corolla pale yellow, anthers dark purple, plants restricted to Veracruz… ………… ***P. greenmanii***
        7. Corolla yellow, anthers dull blue, plants from Jalisco………… ……… ……… ……………8
          8. Flowers on peduncles up to 1.7 cm long, corolla 1.5–2.1 cm in diameter, reflexed flowering calyx lobes………………………… . ***P. lignescens***
          8. Flowers on peduncles up to 4.5 cm long, corolla 2.2–2.4 cm in diameter, erect flowering calyx lobes… …………………… ***P. longipedicellata***
      6. Fruiting calyx 10-costate …………………………9
        9. Pubescence abundant, simple glandular and eglandular trichomes mixed throughout the plant… …… …………………………… .10
        10. Corolla 2.8–4.0 cm in diameter, fruiting calyx 4.0–4.5 cm long, with lanceolate-ovate lobes 11–15 mm long… ………………… . ***P. coztomatl***
        10. Corolla 1.2–2.0 cm in diameter, fruiting calyx 2.5–3.0 cm long, with triangular lobes 4–5 mm long…………………………… . ***P. sancti-josephi***
        9. Pubescence absent, or vestiture of simple eglandular and glandular trichomes scarce………………11
          11. Flower on peduncles 2.5–3 cm long; calyx with deltoid lobes, 3–5 mm long……………………………… . ***P. mcvaughii***
          11. Flower on peduncles 0.5–1.0 cm long; calyx with acuminate lobes, 1.5–2.5 mm long……… ………………………… ***P. tamayoi***

**Key 2: Plants with branched trichomes**

1. Trichomes branched only twice, long multicellular trichomes also present…………………… ……… …………… ***P. vestita***
1. Trichomes branched several times, long multicellular trichomes mostly absent……………………………… ……………2
  2. Leaves spathulate, plants restricted to coastal sand dunes in Tamaulipas………… …… … ………… ***P. spathulifolia***
  2. Leaves never spathulate, plants of other habitats… ………3
    3. Anthers yellow, leaf margins entire, plants of arid zones…………………… ……… …… ***P. cinerascens***
    3. Anthers purple, blue, or blue-tinged but not yellow, leaf margins dentate or undulate; plants of mesic habitats, mostly forests………………………………………4
    4. Fruiting peduncles 1.0–1.6 cm long, plants from Michoacán and Estado de México……… ***P. hintonii***
    4. Fruiting peduncles 2–3 cm long, plants from Nuevo León……………………… …… ***P. hunzikeriana***

**Key 3: Plants glabrous or minutely puberulent**

1. Plants annual……………………………………………2
  2. Corollas urceolate or tubular-campanulate………………3
    3. Corollas tubular-campanulate, whitish; plants prostrate to erect up to 40 cm tall…………………… ***P. microcarpa***
    3. Corollas urceolate, purple; plants erect up to 1 m tall**………… ……… ……… …………*P. solanacea***
  2. Corollas rotate-campanulate………………………………4
    4. Corollas whitish with slightly contrasting maculations, plants of inundated habitats, stems hollow…… ……………… ……… ……………… ………… . ***P. sulphurea***
    4. Corollas pale yellow or whitish but evidently maculated, plants in dry areas, stems solid………………………5
      5. Fruiting calyx strongly 5-angled ………… ***P. cordata***
      5. Fruiting calyx 10-costate… ………………………6
        6. Flowering peduncles 1.8–6 cm long, corolla whitish or pale yellow………… ………… . . ***P. acutifolia***
        6. Flowering peduncles 0.3–1.0 cm long, corolla yellow………… ……………… ……………7
          7. Stems angled, anthers straight after dehiscence ………………………………… ***P. angulata***
          7. Stems terete, anthers convolute after dehiscence …………………………… . ***P. philadelphica***
1. Plants perennial……………………………………… …8
  8. Fruiting calyx 5-angled……… ……… ……… ………9
    9. Plants erect, pubescent only on one side of the stem, restricted to mesophytic forests of Chiapas and Oaxaca …………………………… ……… ………………… . ***P. porrecta***
    9. Plants creeping, glabrous, growing in sand dunes ……….…………………… ……… …………… . ***P. minuta***
  8. Fruiting calyx 10-costate………………………………10
    10. Leaves hastate……………………………………11
      11. Corolla with blue slightly contrasting maculations, plants endemic to Baja California Sur, in sand dunes and oasis……………………………… ***P. glabra***
      11 Corolla with reddish-brown evident maculations, plants endemic to the Mexican Plateau, in desert scrubs……… ……… ……… …… ***P. hastatula***
    10 Leaves ovate or lanceolate, never hastate ***……P. caudella***

**Key 4: Plants with simple eglandular trichomes**

1. Flowers aggregated, with short internodes……… ***P. aggregata***
1. Flowers solitary………………………… ………………2
  2. Corolla purple, with yellowish-brown maculations…… ………………… ……………………… . ***P. purpurea***
  2. Corolla yellow, with almost black, reddish-brown, or dark purple maculations, but never yellowish maculations……3
    3. Plants annual or biannual …………………………4
      4. Fruiting calyx 5-angled…… …… ……… . ***P. grisea***
      4. Fruiting calyx 10-costate… ………… ……… …5
        5. Trichomes on the fruiting calyx with a widened base………………………………… . ***P. ampla***
        5. Trichomes on the fruiting calyx without a widened base…………………………… … . ***P. lagascae***
    3. Plants perennial
      6. Plants decumbent, rooting at the nodes…… … …7
        7. Flowering peduncles 3.5–8.5 cm long, fruiting calyx 5-angled…………………………… … ***P. volubilis***
        7. Flowering peduncles up to 2.5 cm long, fruiting calyx 5-angled or 10-costate… … ………………… .8
          8. Fruiting calyx 5-angled, plants pubescent throughout, internode 6.0–10 cm long, leaf margins entire or dentate…… . . ***P. queretaroensis***
          8. Fruiting calyx 10-costate, pubescence restricted to young parts, internodes up 6 cm long, leaf margins entire…………………………… . . ***P. gracilis***
      6. Plants erect, not rooting at the nodes…… ………9
        9. Stigma capitate, flowering buds apiculate…… …………………………………… ***P. waterfallii***
        9. Stigma clavate, flowering buds rounded………10
          10 Plants with long multicellular trichomes 2–3 mm long, leaves with entire margin…………… …………… … ………11
            11. Flowering peduncles 5–7 mm… … …… ***P. latecorollata***
            11. Flowering peduncles 8–23 mm……………… ***P. subrepens***
          10. Plants with trichomes shorter than 2 mm, leaves with dentate margin…………………… ……… … ………………12
            12. Fruiting calyx lobes 8–14 mm long, narrow, acuminate …………… ……… ………………… . ***P. longiloba***
            12. Fruiting calyx lobes 3–7 mm long, ovate to triangular …………………………………………… ……13
              13. Plants gray due to abundant pubescence, leaf margins with 3–6 teeth per side…………… ***P. chenopodiifolia***
              13. Plants green, leaf margins entire or with 2 or 3 teeth per side…………………………………… ***P. orizabae***

**Key 5: Plants with glandular trichomes, sometimes mixed with simple eglandular trichomes**

1. Plants annual………………………………………… …2
  2. Fruiting calyx 5-angled……… ……… … …… ………3
    3. Corolla with purple maculations……… … ***P. pubescens***
    3. Corolla with usually green, olivaceous, or reddish-brown maculations………… ……… ……… ……………4
      4. Stems, peduncles, and fruiting calyx densely pubescent with gray glutinous short trichomes……… . ***P. ignota***
      4. Stems not densely pubescent, trichomes glandular with orange tips………………………………………5
        5. Fruiting peduncles thick (1.1–2.0 mm), corolla creamy whitish… ……… ……… ***P. nicandroides***
        5. Fruiting peduncles thin (0.5 mm), corolla yellow…6
          6. Plants small, up to 0.8 m high, fruiting calyx 1.5–3.0 cm wide…………………… ***P. patula***
          6. Plants large, higher than 1 m, fruiting calyx 2.5–4.0 cm wide…………… ……… …… .7
            7. Fruiting peduncles 1.0–1.5 cm long, corolla maculations reddish-brown, stems with trichomes of the same size…… . ***P. latiphysa***
            7. Fruiting peduncles up to 5.5 cm long, corolla maculations pinkish-brown, stems with a mixture of long and short trichomes………… … . .……… ……………… ***P. pruinosa***
  2. Fruiting calyx 10-costate………… ……… ……………8
    8. Anthers 1.5–2.5 mm long, blue or blue-tinged; fruiting calyx 1.5–2.5 cm long……… ……… ………… ***P. leptophylla***
    8. Anthers 4 mm long, purple; fruiting calyx 0.9–1.9 cm long……… ……… ……… ……… ***P. minimaculata***
1. Plants perennial………… ……… ……… …………… .9
  9. Leaf margin with large teeth, both surfaces densely covered with large trichomes, plants endemic to Tehuacán in Puebla …………………………………… … . ***P. tehuacanensis***
  9. Leaf margin entire or toothed, but the teeth small; trichomes do not cover the leaf surface………………10
    10. Anthers short, 1.5–1.8 mm long, plants small, shorter than 40 cm high…………… ……… ***P. parvianthera***
    10. Anthers larger than 2 mm, plants larger than 40 cm high…………………………………………… .11
      11. Flowering peduncle <10 mm long………… … .12
        12. Anthers smaller than 3 mm……………… .13
          13. Plants prostrate, fruiting calyx 5-angled…… ……………………… ……… . ***P. angustiphysa***
          13. Plants erect, fruiting calyx 10-costate …………14
            14. Plants small, <20 cm tall, endemic to dry areas in San Luis Potosí…………… … …………… ………… … ***P. pennellii***
            14. Plants larger than 50 cm, distributed in mesic habitats in western and southern Mexico …………………… ………… . ***P. lassa***
      12. Anthers larger than 3 mm…………………15
        15. Plants densely glandular, dirty, corollas 1.5–2.5 cm in diameter ***…… …P. sordida***
        15. Plants glandular but not dirty, corolla 0.7–1.2 cm in diameter………… ……………… ………………16
          16. Leaf blades ovate to subcordate, corollas immaculate …………………………………… ***P. hederifolia***
          16. Leaf blades lanceolate to trulate, corollas evidently maculated… ……… … …… ……… ***P. rydbergii***
      11. Flowering peduncle >11 mm long…… …………… .17
        17. Fruiting calyx 5-angled……… …………. ***P. pringlei***
        17. Fruiting calyx 10-costate…… ………… ***P. crassifolia***

## 8 Species descriptions

1. *Physalis acutifolia* (Miers) Sandwith, Kew Bull. 14 (2): 232 (1960)

**FIGURE 4:**
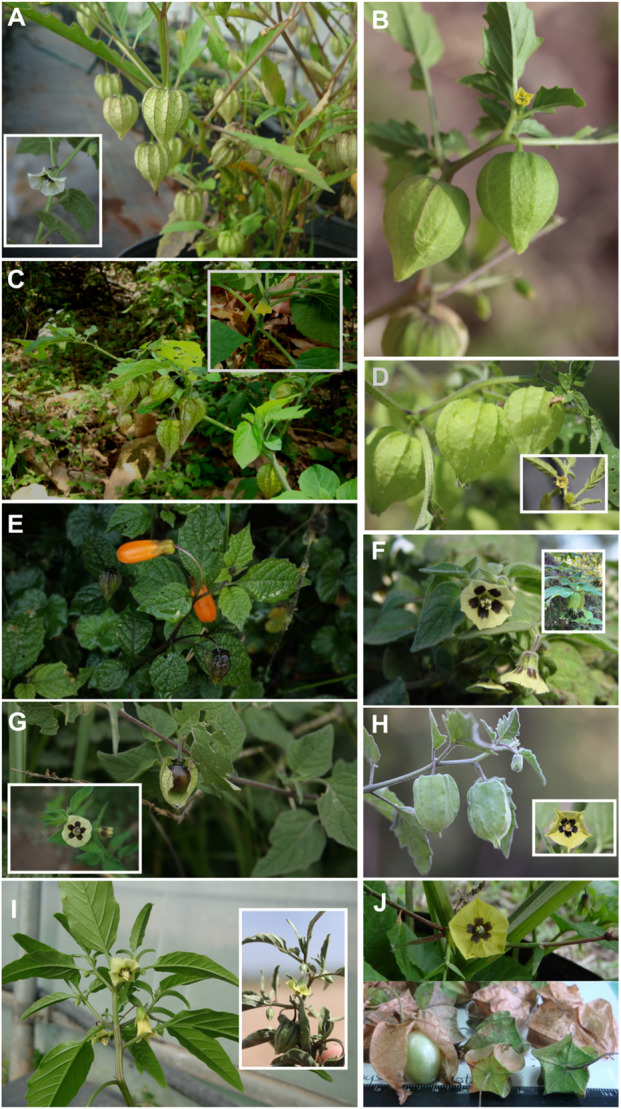
Details of flowers and fruits of *Physalis* spp. **(A)** *P. acutifolia*, **(B)** *P. angulata*, **(C)** *P. aggregata*, **(D)** *P. ampla*, **(E)** *P. campanula*, **(F)** *P. angustiphysa*, **(G)** *P. chenopodiifolia*, **(H)** *P. cinerascens*, **(I)** *P. caudella*, **(J)** *P. cordata.*

**Type**: United States. California, T. Coulter 593 (holotype: K Barcode K000759429!). *Saracha acutifolia* Miers, Ann. Mag. Nat. Hist. ser. 2,3 (18): 449. 1849. (Figures 1A, 4A).

*= Physalis wrightii* Proc. Amer. Acad. Arts x. (1875) 63. Type: USA, Texas, prairies of the San Pedro river, C. Wright 1602 (holotype: GH barcode 00003296!, isotypes: NY barcode 00138864!, US barcode 00027376!, microfilm MEXU!).

Erect annual herb up to 1 m tall; stem highly ramified, glabrous; leaves alternate throughout, petioles 1.1–6.6 cm long; blades lanceolate, 2.8–12.6 cm long, 1.1–6.7 cm wide, apex acute to attenuate, base oblique to cuneate, decurrent, margin sinuate-dentate to serrate, glabrous; flowers solitary on peduncles 1.8–6.0 cm long; calyx with triangular lobes 2–3 mm long, corolla rotate, pale yellow to whitish, 7–17 mm in diameter, with five brownish-to-greenish slightly contrasting maculations, corolla throat pubescent; anthers purple, 1.5–3.0 mm long, sometimes convolute after anthesis; fruiting peduncle 2.5–6.0 cm long, fruiting calyx 10-costate, almost round, 2–3.4 cm long, 1.6–2.4 cm wide, glabrous; mature berry green, ca. 1.5 cm in diameter with numerous yellow seeds ca. 1.5 mm in diameter.

**FIGURE 5:**
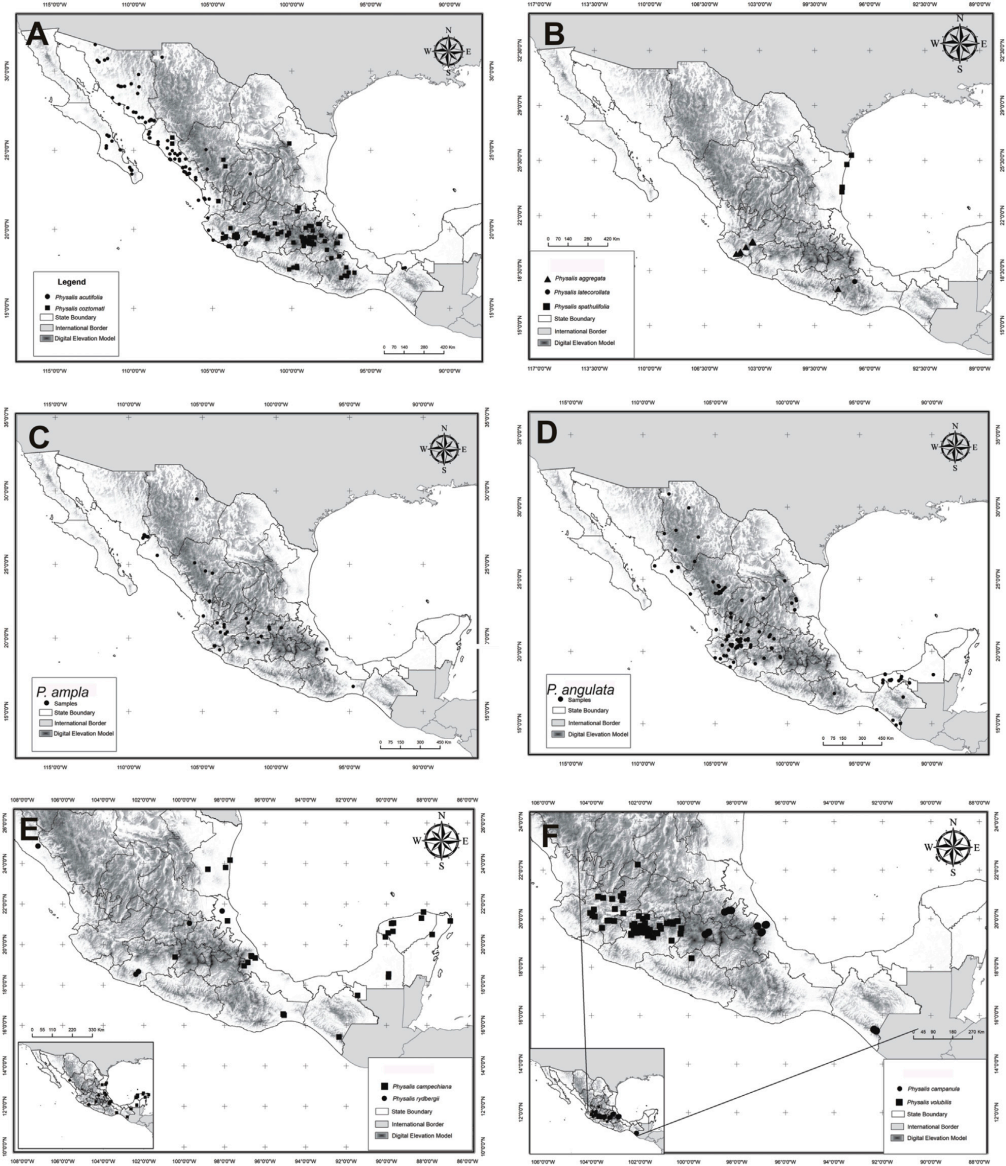
Geographic distribution of *Physalis* spp. **(A)** *P. acutifolia* and *P. coztomatl*; **(B)** *P. aggregata*, *P. latecorollata*, and *P. spathulifolia*; **(C)** *P. ampla*; **(D)** *P. angulata*; **(E)** *P. campechiana* and *P. rydbergii*; **(F)** *P. campanula* and *P. volubilis.*

**Distribution and habitat:** Common in the Pacific slopes on volcanic rocks near sandy riverbanks or coastal vegetation, tropical deciduous forest, or mangroves up to 1,500 m elevation. B.C., B.C.S., Chih., Chis., Col., Camp., Jal., Mich., Nay., Sin., Son., Zac. (Figure 5A).

**Diagnostic characters:** A whitish corolla, serrate leaves, and long flowering and fruiting peduncles.

**Common names and uses:** Tomatillo. The fruits are eaten in Baja California.

**Phenology:** Flowering and fruiting from September to March.

**Conservation status**: LC.

**Representative specimens examined.** MEXICO. **Baja California:** Rocky slope above Ejido San Matias, 31.32911N, −115.54025W, *R.Thorne 57,561* (SD). **Baja California Sur**: Loreto, río Huatamote en el km 58 de la carretera Loreto-Ciudad Insurgentes, *M. Martínez 9525* (QMEX). **Chihuahua:** Janos, border of Chihuahua and Sonora, *S. White 2576* (MEXU). **Chiapas:** Tonalá, tidal marsh near Puerto Arista, *D. E. Breedlove 25,546* (MEXU). **Colima**: Tecoman, upper edge of the lagoon near kilometer post 55 on the highway between Cuyutlán and Armeria, 9 km W of the Rio Armeria bridge, near 18° 55′ N, 104° 01′ W, *Sanders 11,921* (MEXU, MICH). **Campeche:** Palizada, *E. Matuda 3867* (MEXU). **Jalisco**: Tomatlán, near the new road *ca.* 25 km northwest of Río san Nicolás and 20 km southeast of Tomatlán, *R. McVaugh 25,310* (MEXU). **Michoacán:** Tepalcatepec, ejido Tepalcatepec, carretera Coalcomán, *Agundis 1065* (MEXU, ENCB). **Nayarit:** Santiago Ixcluitla a 10 km al W de Unión de Corrientes, camino a Mezcaltitán, *O. Téllez 12,112* (MEXU, MICH, ENCB). **Sinaloa:** 1 km al W de El Quemado, saliendo de El Quelite, municipio de Mazatlán, *M. Martínez 9934* (QMEX). **Sonora:** Mesa del Seri, desviación hacia Sahuaripa, municipio de Hermosillo, *M. Martínez 9834* (QMEX). **Zacatecas:** Jalpa, Huanasco, *R. Hernández 9651* (MEXU).

2. *Physalis aggregata* Waterf., Rhodora 69: 108 (1967)

**Type:** Mexico, Oaxaca, rancho de Calderon, San Juan, L. C. Smith 771 (holotype: GH barcode 3284!). (Figures 2A, 4C).

Erect annual herb up to 1 m tall; stem ramified, glabrous; leaves alternate throughout, petioles 1.2–5.0 cm long; blades ovate or suborbicular, 2.3–9.5 cm long, 2.2–7.0 cm wide, apex acute to acuminate, base oblique, decurrent, margin sinuate to dentate, glabrous; flowers solitary to almost aggregate with short internodes on peduncles 3.0–9.5 cm long; calyx with ovate lobes ca. 1.5 mm long, corolla rotate, yellow, 8–11 mm in diameter, with five dark brown maculations, corolla throat pubescent; anthers blue, 1.5–3.0 mm long; fruiting peduncle 1–16 mm long, fruiting calyx 10-costate, inflated at the base and contracted at the upper third, 2.8–3.9 cm long, 1.8–2.2 cm wide, glabrous; mature berry green, ca. 1 cm in diameter with numerous yellow seeds ca. 1.5 mm in diameter.

**Distribution and habitat:** Open sites in oak forest or ecotone with cloud forest at 1,800–2,100 m of elevation. Jal, Oax. Figure 5B.

**Diagnostic characters:** Flowers aggregated together, or almost, by short internodes, but the mature fruits are solitary.

**Common names and uses**: Tomatillo.

**Phenology:** Flowering in July and August, and the fruits collected in March.

**Conservation status**: VU.

**Representative specimens examined.** MEXICO. **Jalisco**: Jocotepec, camino de ascenso al cerro Viejo, por Las Trojes, *A. Rodríguez 993* (IBUG, IEB, MEXU). **Oaxaca:** Santiago Juxtlahuaca, 1–2 km del poblado El Manzanal, carretera a Infiernillo. Distrito Juxtlahuaca, *J. Calzada 20,724* (MEXU).

3. *Physalis ampla* Waterf., Rhodora 69: 219 (1967)

**Type:** Sinaloa, vicinity of Culiacán, Cofradía, T. S. Brandegee s.n. (holotype: UC barcode 104119!). (Figure 4D).

Erect annual herb 10–30 cm high, stem pubescent with eglandular multicellular divergent trichomes; petioles 0.3–1.5 cm long; blades ovate to rhombic, 2–4 cm long, 1.0–1.5 cm wide, apex acute, base attenuate, margin entire to dentate with three to four teeth per side, pubescent with multicellular eglandular trichomes on both surfaces; flowers solitary on peduncles ca. 2 mm long; calyx with deltoid lobes ca. 1 mm long; corolla rotate, yellow, ca. 3 mm in diameter with five slightly contrasting darker maculations; anthers green or blue, 1–1.2 mm long; fruiting peduncles 3–4 mm long, fruiting calyx 10-costate 1.5–2.0 cm long, 1.5 cm wide, pubescent with multicellular trichomes with a widened base; mature berry spherical ca. 1 cm in diameter with numerous dark brown seeds 1–2 mm in diameter.

**Distribution and habitat:** A small plant of grasslands, tropical deciduous forest, and farming areas; it grows from sea level to 2,000 m. Col., Chih., Dgo., Gto., Jal., Mich., Nay., Oax., Qro., Sin., Son. (Figure 5C).

**Diagnostic characters**: A widened persistent base of the multicellular trichomes in the flowering and fruiting calyx.

**Common names and uses:** Tomate verde. No uses known.

**Phenology:** Flowers and fruits from August to October.

**Conservation status**: LC.

**Representative specimens examined.** MEXICO. **Colima:** Pueblo Juárez, *J. Mallet 12* (MEXU). **Chihuahua:** Temósachic, Nabogame *J. E. LaFerrière 1638* (MEXU). **Durango:** Canantlán, ca. 3 km del pueblo sobre la carretera a Santiago Papasquiaro, *M. Martínez 9745* (QMEX). **Guanajuato:** Santiago Maravatío, cerro Prieto, cerca de La Leona, *Rzedowski 40591* (IEB, MEXU). **Jalisco:** Jocotepec: cerro Viejo, camino que sale del poblado de Zapotitlán de Vadillo a El Aguacero, *O. Vargas 833* (IBUG). **Michoacán:** alrededores de Sanguijuelas, municipio de Churintzio, *J. Rzedowski 51986* (IEB). **Nayarit**: Nayar: 8 km aL NW de san Juan Peyotán, en los límites de Nayarit y Durango, camino a san Juan Peyotán, 22° 30′ N, 104° 30′ W, *Flores 1213* (MEXU). **Oaxaca:** San Miguel Chimalapa, camino Benito Juárez-La Ciénega, al E de la división, ca. 8 km al E de Benito Juárez, ca. 37 km en línea recta al NNE de San Pedro Tapanatepec, *S. Maya 3849* (MEXU). **Querétaro:** Los Manantiales, 1 km aL SE de la presa El Batán, municipio de Villa Corregidora, *L. Hernández 3609* (QMEX). **Sinaloa:** vicinity of Culiacán, Cofradía, T. Brandegee s.n. (UC). **Sonora:** Yécora, Puerto de la Cruz, north base of Mesa del Campanero, *T. Van Devender 96–477* (MEXU).

4. *Physalis angulata* L., sp. Pl. 1: 183 (1753)

Lectotype (designated by D’Arcy in Ann. Missouri Bot. Gard. 60 (3):662. 1974): Anon. s.n. (LINN 247.9!). (Figures 1B, 4B).

Synonyms in Pretz and Deanna (2020).

Erect annual herb 30–40 cm high; stem angular, puberulent with few eglandular trichomes to almost glabrous; basal leaves caducous, petioles 0.5–3.5 cm long; blades elliptic to elliptic-ovate, 2.5–5.0 cm long, 1.5–3.0 cm wide, apex acute, base attenuate, margin dentate with two to seven teeth per side, puberulent on both surfaces; flowers solitary on peduncles 3–5 mm long; calyx with deltoid lobes ca. 2 mm long; corolla rotate, yellow, ca. 10 mm in diameter with five not strongly contrasting blue maculations, corolla throat almost glabrous; anthers yellow with blue lines ca. 2 mm long; fruiting peduncle ca. 1 cm long, fruiting calyx 10-costate, 2.5–3.0 cm long, 1.5–2.5 cm wide, puberulent; mature berry spherical 1.0–1.5 cm in diameter, green at maturity with numerous yellow seeds ca. 2 mm in diameter.

**Distribution and habitat:** A common weed associated with cornfields, rivers, grasslands, pine–oak forests, or tropical deciduous forests; it grows from sea level up to 2,400 m. Ags., B.C.S., Camp., Chih., Chis., Col., Dgo., Gto., Jal., Mich., Nay., N.L., Oax., Qro., Tab., Tamps., Sin., Son., Ver., Zac (Figure 5D).

**Diagnostic characters:** Almost glabrous plant, with a small corolla and 10-costate fruiting calyx.

**Common names and uses:** Tomate, tomate de bote, tomate ajuareño, tomate milpero, tomatillo, miltomate, tumat’cho, tomate silvestre, tomate de tierra fría, tomate cuarentano, tuyuki (huichol). The species is self-pollinated, and spontaneous plants are tolerated and harvested in Jalisco, Querétaro, Chihuahua, and Tamaulipas. The fruits are smaller than those of *P. philadelphica* but are commonly sold in marketplaces.

**Phenology:** Flowering and fruiting from June to March.

**Conservation status**: LC.

**Representative specimens examined.** MEXICO. **Aguascalientes**: Calvillo, área six Calvillo, *Meza, Soto y León 505* (IBUG). **Baja California Sur**: Comondú, Sierra La Giganta, Rancho Viejo El Metate, 15 km al E. de Los Llanos de San Julio, *M. Domínguez 3359* (HCIB). **Campeche:** Calakmul, a 17 km al S de la caseta de entrada a Calakmul, *E. Martínez 28,93*0 (MEXU). **Chiapas:** Acapetahua, ejido Matamoros a 15 km aL sureste del poblado de Acapetagua, *R. Trujillo 20* (MEXU). **Chihuahua:** arroyo Batopilas, a orillas del río, *M. Martínez 9673* (QMEX). **Colima**: Colima, Comaquitla, *Sánchez-Ken 436* (MEXU). **Durango**: Puente Morteros, al NE de Durango, *González 2734* (CIIDIR-DGO). **Guanajuato**: Yuridia: 8 km al E de Yuridia, sobre la carretera a Salvatierra, *Rzedowski 40,314* (IEB). **Jalisco**: Jocotepec, camino de ascenso al cerro Viejo, por Las Trojes, *Rodríguez 994* (IBUG, IEB, MEXU, WIS). **Michoacán:** El Cerrito, 1 km al E de Jerécuaro, municipio de Zinapécuaro*, M.J. Jasso 1441* (IEB, QMEX). **Nayarit:** Santiago Ixcuintla, *O Tellez 12112* (MO). **Nuevo León:** San Francisco de los Blancos, afuera de Galeana, *M. Martínez 9636* (QMEX). **Oaxaca:** Sola de Vega, 25 km al S de Sola de Vega, *J. Linares 4452* (MEXU). **Querétaro:** Alrededores de La Llave, Municipio de San Juan del Río, *J. Rzedowski 50,167* (IEB, QMEX). **Tabasco:** Jonuta, *R. Fernández 1479* (MO). **Tamaulipas**: km 124 de la carretera Victoria-Miquihuana, ejido José María Morelos, *M. Martínez 9494* (QMEX). **Sinaloa:** Culiacán, a 20 km al N de Culiacán, carretera Culiacán a Guamúchil, *R.Vega 6360* (UAS). **Sonora:** Rancho Santa Bárbara, 42.3 km, ENE de Álamos, *T. Van Devender 2006–126*7 (ARIZ) **Veracruz:** Veracruz de Ignacio de la Llave, 1.6 km del poblado La Yerbabuena, sobre la carretera de La Yerbabuena a El Ojital, *M. Vázquez s. n* (XAL). Zacatecas: 3 km al W de Pueblo Viejo, Sierra de Morrones, cerro de Piñones, *J. Balleza 8821* (MEXU).

5. *Physalis angustior* Waterf., Rhodora 69: 114 (1967)

**Type:** Mexico, Morelos, Sierra de Tepoztlán, C. G. Pringle 7731 (holotype: VT, isotype: US barcode: 00027313!)

Shrub or suffrutescent, 1–2 m high, densely covered with articulated and partly glandular trichomes; petioles 3–5 cm long; blades ovate, 6–10 cm long, 4–8 cm wide, apex acuminate, base attenuate, margin entire or with one or two teeth per side; flowers solitary or 2–3-aggregated on peduncles 12–20 mm long; calyx lobes lanceolate-tapered 5–9 mm long; corolla yellow, 20–29 mm wide with five dark maculations, anthers violet, 3–4 mm long; fruiting peduncles 15–30 mm long, fruiting calyx 25–30 mm long and 15–20 mm wide; berries 12–15 mm in diameter.

**Distribution and habitat:** Tropical deciduous forest; grows at 2,200 m asl. Mor.

**Diagnostic characters:** Suffrutescent with large acuminate leaves.

**Common names and uses**: Unknown.

**Phenology:** Collected from November to May with flowers and fruit.

**Conservation status:** Data deficient (DD), possibly extinct as no recent collections are known of.

**Representative specimens examined.** MEXICO. **Morelos:** Sierra de Tepoztlán, *C.G. Pringle 7731* (MEXU).

6. *Physalis angustiphysa* Waterf., Rhodora 69: 228 (1967)

**Type:** Guatemala, Huehuetenango La Sierra, Steyermark 51977 (holotype: US barcode 00027314!, isotype: F catalog 1148724!, US barcode 02824504!). (Figures 1C, 3H, 4F).

Prostrate perennial herb 20–100 cm high; stems densely villous with multicellular glandular trichomes; blades ovate to deltate, 3–10 cm long, 3.5–7.0 cm wide, apex acute, base cordate, margin entire, sometimes dentate, villous on both surfaces; flowers solitary on peduncles 4.5–9.0 mm long; calyx with triangular lobes 2.5–3.5 mm long, corolla rotate, yellow, 1.0–1.5 cm in diameter with five strongly contrasting purple maculations, corolla throat densely pubescent; anthers blue, 2.8 mm long; fruiting peduncle 4.5–9.0 mm long, fruiting calyx strongly 5-angled, 2–3 cm long, ca. 1.5 cm wide, villous; mature berry spherical 1.5 cm in diameter with numerous yellow seeds ca. 2 mm in diameter.

**Distribution and habitat:** Tropical deciduous forest; pine and oak forest; grows from 800 to 2,100 m. Chis., Gto., Jal., Mich., Nay., Son., Tamps.

**Diagnostic characters:** A rare perennial, villous, and glandular plant with a narrow fruiting calyx.

**Common names and uses:** Chush-gutz, tomate, tomate zope, miltomate, chivol-antivo. No known uses.

**Phenology**: Flowering and fruiting from August to November.

**Conservation status**: LC.

**Representative specimens examined.** MEXICO. **Chiapas:** San Cristóbal de las Casas-Joigelito, Zinacatán, *B. López 641* (MEXU). **Guanajuato**: Ocampo, mas ó menos 4 km al S de Santa Bárbara, *E. Carranza 4314* (IEB, MEXU). **Jalisco:** Autlán de Navarro, Las Galeras, Sierra de Manantlán, *Vargas 861* (IBUG). **Michoacán:** Cerro la Alberca, municipio de Villa Jiménez, *E. Pérez y E. García 2590* (IEB, QMEX). **Nayarit**: A 7 km al W del Venado, camino a San Pedro Ixcatán y El Zopilote. O. Téllez Valdés y Gabriel Flores F (TEX). **Sonora:** El Divisadero, 1 km SE of El Llano on road to Bermúdez, *T. R. Vandevender 97–662* (QMEX). **Tamaulipas:** Jaumave, Balcón del Chihue, *M. Martínez 9475* (QMEX).

7. *Physalis campanula* Standl. & Steyerm., Publ. Field Mus. Nat. Hist., Bot. Ser. 23: 18 (1943)

**Type:** Guatemala: San Marcos: dry banks along upper part of Quebrada Canjula, between Sibinal and Canjula, Volcán Tacaná Steyermark 36067 (holotype: F catalog 151991!). (Figures 1D, 4E).

= *Physalis constricta* Waterf., Rhodora 69: 99 (1967). Mexico: Oaxaca, Ghiesbreght s.n. (holotype: P MNHN-P-P00387526)

Shrub 30 cm high; stem with multicellular glandular trichomes; blades ovate, 3–6 cm long, 2.5–4.0 cm wide, apex acute to acuminate, base attenuate, margin entire to dentate with one to five teeth per side, glandular villous on both surfaces; flowers solitary on peduncles 2.0–4.5 cm long; calyx with triangular lobes ca. 3 cm long, ca. 3 mm wide; corolla tubular, orange or yellow, 1.5–2.0 cm long, 1 cm wide, without contrasting maculations; anthers 4.5 mm long; fruiting calyx 10-costate, 2–3 cm long, 1.5 cm wide, densely glandular-pubescent; mature berry yellowish, spherical ca. 1.5 cm in diameter.

**Distribution and habitat:** Pine and oak forest, riparian vegetation; it grows from 2,200 to 3,000 m. Chis., Hgo., Oax., Ver. (Figure 5F).

**Diagnostic characters:** A rare shrub with orange, immaculate tubular corollas with the limb obscurely dentate.

**Common names and uses**: None known.

**Phenology**: Flowers and fruits from February to April.

**Conservation status**: NT.

**Representative specimens examined.** MEXICO. **Chiapas:** Motozintla, on the north and west slopes of the Cerro Mozotal below the microwave tower along the road from Huixtla to El Porvenir and Siltepec, *D. Breedlove 25,859* (MEXU) **Hidalgo**: Tenango de Doria, km 9 carr. 154, 8.8 km aL O de Cruz Tenango *P. Zamora Tavares et al. 260* (IBUG) **Oaxaca:** Ghiesbreght s.n*.* (P) **Veracruz**: Chiconquiaco, Arroyo Resbaloso, Ventura 4882 (ENCB, XAL).

8. *Physalis campechiana* L., Syst. Nat., ed. 10. 2: 933 (1759)

Mexico, Campeche Houston s.n. (BM). Type not designated. Original material category “figure” according to Natural History Museum (2022).

**=** *Physalis arborescens* L., sp. Pl., ed. 2. 1: 261 (1762). Lectotype designated by Waterfall (1967). *Physalis* in Mexico, Central America, and the West Indies. Rhodora 69 (777): 95. The name is illegitimate according to the Natural History Museum (2022).

= *Physalis mayana* Standl., Publ. Field Mus. Nat. Hist., Bot. Ser. 8: 42 (1930). Type: Mexico: Yucatán: without definite locality, G.F. Gaumer 24,381 (holotype: F barcode V0077207F!, isotype: US barcode 00027339!).

Shrub with few ramifications 80–450 cm high; stems with abundant dendritic trichomes, glabrescent; blades elliptic to ovate, 5–14 cm long, 2–7 cm wide, apex acute, base decurrent, margin entire, rarely crenate-dentate with six teeth per side, with dendritic trichomes on both surfaces; flowers aggregated in two to seven flowered fascicles, peduncles ca. 1 cm long; calyx with triangular lobes, tomentose with short, branching trichomes; corolla 5-lobed, yellowish, 8–12 mm long, the lobes 3–5 mm long, with dark maculations; anthers yellow, ca. 4 mm long; fruiting peduncles up to 1.2 cm long, fruiting calyx 10-costate, purple ca. 3 cm long, ca. 2 cm wide; mature berry bright red 7–11 mm in diameter with numerous yellow seeds 1.4–1.6 mm in diameter.

**Distribution and habitat:** Tropical deciduous forest; it grows from sea level up to 400 m. Camp., Chis., Oax., Q. Roo, Tab., Tam., Ver., Yuc. (Figure 5E).

**Diagnostic characters:** Along with *P. melanocystis*, it presents a shrubby habit and fascicled flowers, but *P. melanocystis* has simple trichomes while *P. campechiana* has branched ones.

**Common names and uses:** Tomatillo, tamaliyo, tomate. No uses known.

**Phenology:** Flowering and fruiting throughout the year.

**Conservation status**: LC.

**Representative specimens examined.** MEXICO. **Campeche:** Calakmul, a 37 km al S de la Caseta de entrada a Calakmul, *E. Martínez 28,151* (MEXU). **Chiapas:** *D. Breedlove 11800* (DS). **Oaxaca:** Agua Tibia, 500 m al N de Nizanda, *E. Pérez 1105* (MEXU). **Quintana Roo:** Tulum Sakbe' 3 a San Pedro *R. López 276* (QMEX). **Tabasco:** Tenosique, a 2.6 km arriba del Cerro, en el campamento de la Escollera y 5 km de Tenosique hacia E. Zapata *M. A. Magaña 843* (MEXU). **Tamaulipas:** Soto la Marina, 27.5 km del camino de Soto la Marina a la Pesca, *J. Jiménez 454* (MEXU). **Veracruz**: Tepetzintla, San José de Copaltitla, 7 km al NE de Tepetzintla *G. Castillo 2296* (QMEX). **Yucatán:** Río Lagartos 2 km E del crucero Río Lagartos rumbo a Las Coloradas, *P. Simá 2332* (MEXU).

9. *Physalis caudella* Standl., Publ. Field Mus. Nat. Hist., Bot. Ser. 17: 273 (1937)

**Type:** Mexico, Chihuahua, Cajurichi, Rio Mayo, H.S. Gentry 2710 (holotype: F barcode V0072995F9!, isotypes: ARIZ catalog 274368!, GH catalog 77344!, MEXU-27636, MO 503577, S, UC 582052, US barcode 27318!). (Figure 4I).

Synonyms in Pretz and Deanna (2020).

Erect perennial herb with long and short trichomes; petioles 5–20 mm long, blades ovate-lanceolate to linear-lanceolate, 4–8 cm long, (6–) 15–40 mm wide, apex acute, base attenuate, decurrent, margins entire to irregularly undulate; flowers solitary on peduncles 5–10 mm long; calyx with acuminate lobes 5–8 mm long, corolla rotate, yellow, ca. 2.2 cm wide with five reddish-blue or purplish maculations not strongly contrasting, or lacking; anthers bluish, ca. 3 mm long; fruiting peduncle 1.4–1.9 cm long, fruiting calyx 10-costate, 2.5–5.0 cm long and 2.5–3.0 cm wide, glabrous; mature berry yellow or orange, 1.0–1.3 cm in diameter with numerous yellow seeds ca. 2 mm in diameter.

**Distribution and habitat:** Open sites in pine–oak forest and farming areas; it grows from 2,000 to 2,200 m asl. Chih., Coah., Dgo., Hgo., Gto., N.L., Qro., S.L.P., Son., Tamps., Zac. (Figure 7A).

**Diagnostic characters:** A perennial with bluish anthers, purplish and not strongly contrasting maculations, and vestiture of spreading, jointed trichomes, long and short intermixed.

**Common names and uses:** Tomatillo, tomate borracho. No uses known.

**Phenology:** Flowering and fruiting from July to October.

**Conservation status**: LC.

**Representative specimens examined.** MEXICO. **Chihuahua:** camino Ocampo-Moris, km 53, Municipio de Moris, *M. Martínez 966*6 (QMEX). **Coahuila:** Tanque Nuevo, *M. Martínez 9605* (QMEX). **Durango**: Cuencamé, ejido Las Mercedes, Campo experimental de Zonas Áridas, aprox. 19 km al N de Cuencamé, carr. 40 Durango-Torreón, 24° 52′ N y 103° 42′ W**,** *A. Rodríguez 1501* (IEB). **Hidalgo:** El Arenal, 1 km al E de el Arenal, *M. López 103* (MEXU). **Guanajuato**: Ocampo, 4 km aL SW de La Escondida, *J. Rzedowski 52,206* (IEB, MEXU). **Nuevo León:** Doctor Arroyo, km 29 carretera a Doctor Arroyo*, M. Martínez 9468* (QMEX). **Querétaro:** Municipio de Landa de Matamoros, misión de Tilaco, a 25 km de Landa de Matamoros, *M. Martínez 2790* (QMEX). **San Luis Potosí:** 2 km al W de Estación Catorce, hacia Santa María del Refugio, *M. Martínez 9557* (QMEX). **Sonora**: Puerto de Los Aserraderos, *S. White 3207* (MEXU). **Tamaulipas:** km 23 a Miquihuana, *M. Martínez 9463* (QMEX). **Zacatecas:** San Tiburcio, ca. 3 km al E del entronque hacia Cedral, M. Martínez 9830 (QMEX).

10. *Physalis chenopodiifolia* Lam., Tabl. Encycl. 2 (3.1): 28 (1794)

**Type:** Peru, Anon. s.n. (holotype: P-LAM) (Figures 1F, 2F, 4G).

= *Physalis puberula* Fernald, Proc. Amer. Acad. Arts 36: 502 (1901). Type: Mexico, State of Mexico, Sacro Monte, Amecameca, C.G. Pringle 9147 (lectotype designated by Pretz and Deanna (2020) MEXU barcode MEXU 00027679!, isolectotypes: CAS barcode 0004034, F barcode V0073008F, GH barcode 00077364, MICH barcode 1109898, NMC 42525, RM barcode RM0004396, US barcode 00027360, VT barcode UVMVT026427). Pretz and Deanna interpreted the species as a synonym of *P. hederifolia* A. Gray. However, the leaves of *P. puberula* have entire margins and angled fruiting calyces similar to *P. chenopodiifolia.* Furthermore, *P. hederifolia* grows mostly in the northern states of Mexico.

= *P. chenopodiifolia* var. *chenopodiifolia* forma *immaculata* Waterf., Rhodora 69 (777): 110 (1967). Type: Mexico, Tlaxcala, sunny banks between cultivation, Mt. Malinche, Balls 4861 (holotype: US barcode 00027354!)

= *P. chenopodiifolia* var. *viridis* Waterf., Rhodora 69 (777): 110–111 (1967). Type: Mexico, Durango, Otinapa, E. Palmer 420 (holotype: US barcode 00027321!, isotypes: F barcode V0072998F!, GH 00077346!, NY barcode 138868!, UC 140093!).

= *P. chenopodiifolia* var. *exigua* Waterf,. Rhodora 69 (777): 110–111 (1967). Type: Mexico, San Luis Potosí, Charcas. C. Lundell 5398 (holotype: MICH 1109909!, isotype: US barcode 00027320!).

= *P. chenopodiifolia* var. *glandulosa* Waterf., Rhodora 69 (777): 111 (1967). Type: Mexico, Querétaro, cliffs on steep mountainsides, 22 miles NE of Zimapan, U.T. Waterfall 14170 (holotype: OKLA, isotypes: MICH, US. None of the cited specimens were located.

Erect perennial herb, up to 70 cm tall, with dense, short pubescence throughout the plant, giving it a cinereous appearance; petioles 0.6–2.3 (−3.2) cm; blade ovate to lanceolate 1.7–6.7 cm long, 0.7–4.1 cm wide, apex acute, suddenly attenuated, base oblique, decurrent, margin entire or with three to six teeth per side; flowers solitary on peduncles 9–15 mm long; calyx with narrow triangular to ovate, attenuate or subulate lobes 1.5–4.2 mm long; corolla rotate campanulate, yellow, 1.6–3 cm in diameter, with five strongly contrasting reddish-brown maculations, corolla throat densely pubescent; anthers blue, 2–3.5 mm long; fruiting peduncle 0.6–2.2 cm long, fruiting calyx 10-costate often with five more prominent angles, 2.2–3.4 cm long, 1.7–2.4 cm wide; mature berry green or purple-tinged, 1.3 cm in diameter with numerous yellow seeds ca. 2 mm in diameter.

**Distribution and habitat:** Open sites or pine–oak margins, around crops or roads, and hillsides with woody vegetation or in secondary grasslands. It develops between 2,000 and 3,100 m asl. Ags., Chih., Cd. Mex., Dgo., Edo. Mex., Gro., Gto., Hgo., Jal., Mich., N. L., Pue., Qro., S.L.P., Tlax., Zac. (Figure 7B).

**Diagnostic characters:** A perennial plant with cinereous appearance and strongly contrasting corolla maculations. The plants grow in different conditions, and the species is highly variable. However, we do not consider the variations to merit different names, as proposed by Waterfall (1967).

**Common names and uses:** Oxcones, tomate, tomatito, chapindikua, jaltomatillo. Roots, stems, and fruiting calyces are used in traditional medicine (Santiaguillo Hernández and Blas, 2009; Valdivia-Mares et al., 2016). The fruit is eaten fresh in the states of Mexico, Puebla, and Tlaxcala by the Mazahua population.

**Phenology:** Flowering and fruiting from June to October.

**Conservation status**: LC.

**Representative specimens examined.** MEXICO. **Aguascalientes:** San José de Gracia, 1/2 km antes de llegar a La Congoja, *O. Vargas 773* (IBUG). **Chihuahua:** Balleza, 46 km al E. de Cabórachi, entre Cabórachi y Baquiriachi *R. Hernández 8963* (MEXU). **Ciudad de México:** Coyoacán, Reserva del Pedregal de San Ángel *L. Mera 144* (MEXU. **Durango**: Nombre de Dios, *González-Elizondo 1042* (CIIDIR-DGO, MEXU). **Estado de México**: Huixquilucan Cerca de la presa “El Capulín,” Fraccionamiento La Herradura *J. Rzedowski 25,874* (MEXU). **Guerrero:** Ayutla de los Libres, Roca Colorada, *A. Díaz 192* (MEXU). **Guanajuato:** 4 km aL SW de La Escondida, municipio de Ocampo*, J. Rzedowski 52,206* (IEB, QMEX). **Hidalgo:** Santiago de Anaya, *A. Soriano 177* (MEXU). **Jalisco:** Acatic, 100 m adelante del entronque de la carretera Tepatitlán-Acatic, *O. Vargas 900* (IBUG). **Michoacán**: cerca de la Cima, municipio de Epitacio Huerta, *J. Rzedowski 49,579* (IEB, QMEX). **Nuevo León:** 14 km al N de San Lázaro, 3 km de la desviación hacia La Joya, *M. Martínez 9572* (QMEX). **Puebla:** supercarrtera a la altura de Estación, *W. Boege 166* (MEXU). **Querétaro:** 7 km aL SE de Tancoyol, municipio de Jalpan, *R. Fernández 3488* (ENCB, IEB). **San Luis Potosí:** 4 km hacia Alamillos, Sierra de Catorce, *M. Martínez 9559* (QMEX). **Tlaxcala:** Ixtenco, 3 km al W de San Juan Ixtenco sobre el camino para la Malinche, *D. Williams 112* (MEXU). **Zacatecas**: San Pedro Piedra Gorda: cerro Prieto, *Trujillo ZA-119* (ENCB).

11. *Physalis cinerascens* (Dunal) Hitchc., Key Spring Fl. Manhattan (1894) 32

**Type:** Mexico, Matamoros, circa Matamoros urbem, J. L. Berlandier 2316 (lectotype designated by Waterfall Rhodora 60 (713)136: GH barcode 00077295!, isolectotypes: F barcode V0073006F!, NY barcode 00138882!). *Physalis pensylvanica* var. *cinerascens* Dunal, Prodr. 13:435–436. 1852. (Figures 1E, 4H).

Synonyms in Pretz and Deanna (2020).

Prostrate to slightly erect perennial herb, branched from the base, densely pubescent, with stellate trichomes throughout; leaves alternate or geminate, petioles 0.5–3.5 cm long; blades ovate, suborbicular, or triangular-ovate, 1.5–6.7 cm long, 1.3–5.4 cm wide, apex obtuse to acute, base cuneate to truncate, decurrent, margin entire; flowers solitary on peduncles 0.6–2.2 cm long; calyx with deltoid lobes 1–2 mm long, corolla rotate, yellow, 8–13 mm in diameter, with five dark-brown-to-black maculations; anthers yellow, 3–4 mm long; fruiting peduncle 1.3–6 cm long, fruiting calyx 10-costate, nearly cylindrical, 1.4–3 cm long, 1.1–2.1 cm wide; mature berry yellow, ca. 1 cm in diameter with numerous yellow seeds ca. 2 mm in diameter.

**Distribution and habitat:** The species thrives in dry places, on limestone slopes with grassland, scrub, or tropical deciduous forest, in disturbed areas; it grows at 1,700–2,100 m asl. Ags., Camp., Chis., Coah., Edo. Mex., Gto., Hgo., Jal., Mich., N.L., Oax., Pue., Qro., S.L.P., Tamps., Ver., Yuc., Zac. (Figure 7C).

**Diagnostic characters:** Densely pubescent throughout with stellate trichomes; dark maculations that contrast with the yellowish corolla.

**Common names and uses:** Agush, costomate, jaltomate, oshcon de milpa, tabardillo blanco, tomatillo, xpajacan, pak-canul, xpakil, tomate, tomate silvestre, tomate de monte, pichandra. The plant is used against gastrointestinal ailments and skin infections and eaten fresh or used for the preparation of sauces (Santiaguillo Hernández and Blas, 2009; Martínez 2020).

**Phenology:** Flowering and fruiting from October to May.

**Conservation status:** LC.

**Representative specimens examined.** MEXICO. **Aguascalientes:** Rincón de Romos: Pabellón, *S. M. L. 728* (IBUG). **Campeche:** 3 km al S de Bolonchen de Rejón en los alrededores de la gruta de Xtacum-bilxuan, *E. Cabrera 8544* (MEXU). **Chiapas:** La Encañada, Ocozocuautla, *F. Pimentel 77* (MEXU). **Coahuila:** Tanque Nuevo, *M. Martínez 9607* (QMEX). **Estado de México**: Carreta Coacalco-Tultitlán, 1 km de Tultitlan, cabecera de municipio, *F. Espinosa 535* (MEXU). **Guanajuato:** Acámbaro: 5 km al W de Iramuco, sobre el camino a Santa Ana Maya, *J. Rzedowski 44,860* (IEB, ENCB). **Hidalgo:** SW of Zimapan ca. 1.5 km at crossing of Arroyo Santiago and the road to Estanzuela, *M. Mayfield 847* (MEXU). **Jalisco**: Lagos de Moreno, Est. Pedrito, Lagos de Moreno, a la orilla de la vía del ferrocarril*, Flores Nava 35* (IBUG). **Michoacán**: Morelia, Francisco Mujica, 9 km aL NE de Morelia, carretera a Maravatío, *J. Soto Núñez 6450* (ENCB). **Nuevo León**: 9.5 km al N de Aramberri, *M. Martínez 9624* (QMEX). **Oaxaca:** Ciudad Ixtepec, al pie del cerro La Pedrera. 14 km al N de la Ventosa, *M. Martínez 1905* (MEXU). **Puebla:** Zinacatepec, 3 km al E de Zinacatepec, *P. Tenorio 17184* **Querétaro:** 3–4 km al N de las Flores, rumbo al río Santa María, municipio de Jalpan, *E. Carranza 3154* (IEB, QMEX). **San Luis Potosí:** 5 km al N de Matehuala, hacia entronque San Roberto, *M. Martínez 9833* (QMEX). **Tamaulipas:** Matamoros, parque industrial en avenida División del Norte, *A. Herrera 9705* (QMEX). **Veracruz:** 2 km al N de Paso del Toro o Concepción, 5 km aL NE de 6 de Enero, *M. Martínez 1906* (MEXU). **Yucatán:** Dzidzantún, paraje a orilla del poblado San Francisco Manzanilla, *J. Santiaguillo 8* (QMEX). **Zacateca**s: 15 km aL SE de Sain Alto, *M. Martínez 9784 a* (QMEX).

12. *Physalis cordata* Mill., Gard. Dict., ed. 8. n. 14 (1768)

**Type:** Mexico, Veracruz, Houston s.n. (holotype: BM = BH neg 5099!). (Figures 1J, 2B, 4J).

Synonyms in Martínez (1998). Pretz and Deanna (2020) included *P. porrecta* Waterf. as a synonym; however, the original description states that the plant has articulated trichomes, whereas *P. cordata* is glabrous.

Erect annual herb, branched, up to 90 cm tall, glabrous; leaves alternate, petiole 1.5–8.6 cm long; blades ovate 8.5 (−12) cm long, 2–7.8 (−9) cm wide, apex acuminate, base oblique to cuneate, decurrent, margin serrate, glabrous; flowers solitary on peduncles 0.6–1.7 cm long; calyx with narrow triangular lobes 3.0–5.2 mm long, corolla campanulate rotate, yellow, 1.1–2.1 cm in diameter, with five purplish-brown maculations, corolla throat densely pubescent; anthers blue or purple, 2.2–4.0 mm long; fruiting peduncle 2.2–3.2 cm long, fruiting calyx 5-angled, 2.2–4.1 cm long, 1.5–3.0 cm wide, glabrous; mature berry spherical to ovoid, 1.2–1.3 cm in diameter, green or purple with yellow seeds 2–3 mm in diameter.

**Distribution and habitat:** A common plant of sandy soils near humid places, such as riparian or secondary and ruderal vegetation, associated with tropical deciduous or sub-deciduous forest; it develops from sea level to 1,300 m. Camp., Chis., Col., Gro., Jal., Nay., Mich., Oax., Sin., Tab., Ver., Yuc. (Figure 7D).

**Diagnostic characters:** In herbarium specimens, *Physalis cordata* is recognized by the shiny, finely toothed leaves, the glabrous 5-angled fruiting calyx, and peduncles up to 3.2 cm long.

**Common names and use:** Tomatillo miltomate; in Jalisco, the fruits are called *corazón de gato* and *corazón de pollo* according to their size.

**Phenology:** Flowering and fruiting from September to February.

**Conservation status**: LC.

**Representative specimens examined.** MEXICO. **Campeche:** Tuxpeña, *C. Lundell 936* (CAS; F). **Chiapas:** Esperanza, Escuintla, *E. Matuda* 16,484 (F). **Colima:** Tecomán, Cofradía de Morelos*, Maillet 425* (MEXU). **Guerrero**: 16 km of Acapulco on Rte 95, *J. Miller 295* MEXU). **Jalisco**: Cihuatlán, coastal plain near the highway to Autlán, four miles north of Bahía Navidad, *R. McVaugh 20,843* (MEXU, MICH). **Michoacán:** 5 km camino Aquila-La Placita, *B. Guerrero 166* (MEXU). **Nayarit:** Bahía de Banderas, 5 km al N del río Ameca, *Puga 9708* (IBUG). **Oaxaca:** vicinity of Cafetal Concordia, *C. Morton 2556* (US). **Sinaloa:** Santa Fe, *Ortega 4384* (US). **Tabasco:** Balancan Ojo de Agua*, J. Santiaguillo 1* (MEXU). **Veracruz:** San Andrés Tuxtla, Estación de Biología Tropical Los Tuxtlas, *G. Ibarra 1989 (MEXU).* **Yucatán:** Izamal, *Gaumer s.n.* (MEXU).

13. *Physalis coztomatl* Mociño & Sessé ex Dunal, Prodr. [A. P. de Candolle] 13 (2): 450 (1849)

**FIGURE 6:**
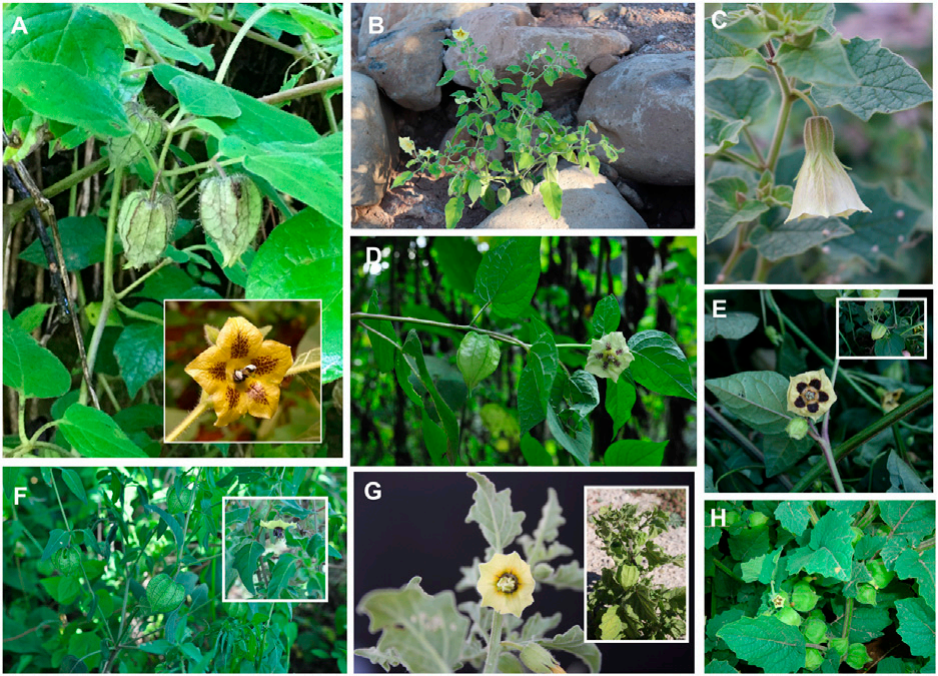
Details of flowers and fruits of *Physalis* spp. **(A)** *P. coztomatl*, **(B)** *P. crassifolia*, **(C)** *P. glutinosa*, **(D)** *P. greenmanii*, **(E)** *P. gracilis*, **(F)** *P. hastatula*, **(G)** *P. hederifolia*, **(H)** *P. grisea.*

**Type:** Drawing 48 of Sessé and Mociño at the Hunt Institute (holotype Copy 916 of Icones florae mexicanae ineditae). (Figures 1G, 6A).

= *Physalis stapelioide*s (Regel) Bitter, Repert. Spec. Nov. Regni Veg. 18: 5 (1922). *Saracha stapelioides* Regel, Suppl. Hort. Bot. Petropol. 18. 1864. Type not located, could be at LE or ZT (Thiers 2016).

= *Physalis acuminata* Greenm. Proc. Amer. Acad. Arts 35: 311 (1900). Type: Mexico: State of México: collected on the Sierra de las Cruces, C. G. Pringle 5315 (holotype: GH 00077342!, isotypes: F barcode V0072994F!, MEXU-28024).

Suffrutescent perennial branched from the base, up to 2 m high, pubescent with eglandular and glandular trichomes; leaves alternate or geminate, petioles 1.4–6.5 cm long; blades ovate to suborbicular ovate 5.7–19 cm long, 3.9–17 cm wide, apex acuminate, base cuneate, truncate to subcordate, decurrent, margin entire or with one or a few teeth, sometimes serrated; flowers solitary on peduncles 14–28 mm long; calyx with lanceolate-ovate lobes 11–15 mm long, corolla rotate, yellow, 2.8–4.0 cm in diameter with five reddish-brown-to-purple compound maculations, corolla throat densely pubescent; anthers purple, 3–5 mm long; fruiting peduncle 1.8–4.0 cm long, fruiting calyx 10-costate, pubescent, somewhat coriaceous, 4.0–5.7 cm long, 2.8–3.4 cm wide; mature berry green, ca. 2 cm in diameter with numerous yellow seeds ca. 2 mm in diameter.

**Distribution and habitat:** In pine, oak, *Abies*, or cloud forest, from 2,300 to 3,100 m. Cd. Mex., Edo. Mex., Gro., Hgo., Jal., Mich., Mor., Nay., Oax., Pue., Qro., Tlax., Ver. (Figure 5A).

**Diagnostic characters:** A large fruticose plant with dense pubescence, with large corollas and compound maculations; it grows at high altitudes.

**Common names and uses:** Coztomate; the plant is used as a diuretic and against stomach inflammation, diarrhea, and flatulence. Applied directly to the chest area, it helps against asthma (Hernández 1943).

**Phenology:** Flowering and fruiting throughout the year.

**Conservation status**: LC.

**Representative specimens examined.** MEXICO. **Ciudad de México:** Parque Nacional Desierto de los Leones, *E. Maldonado 7* (MEXU). **Estado de México:** Tlalmanalco, San Rafael *E. Matuda 27,578* (MEXU). **Guerrero:** Chilpancingo de los Bravo, 6 km aL NW de Omiltemi. Brecha Chilpancingo-Oniltemi-Las Joyas, *P. Tenorio 2588* (MEXU). **Hidalgo:** Tenango de Doria, 10 km al W de Tenango de Doria, *R. Hernández 4303* (MEXU). **Jalisco**: Ciudad Guzmán, km 16 de la brecha El Fresnito-La Joya, Nevado de Colima, *Ramírez Delgadillo 1730* (IBUG, WIS). **Michoacán:** Sierra de San Joaquín, 11 km al S de Tlalpujahua, municipio de Tlalpujahua, *S. Moreno 199* (IEB). **Morelos:** Huitzilac al N del Volcán Quimitepec, Sierra de Chichinautzin, *Y. González 1985* (MEXU). **Nayarit:** Nayar, arroyo de los Negros, ejido Colorado de la Mora, a 500 m del poblado, 21° 47′ N, 104° 38′ W, *Álvarez 187* (MEXU, MICH). **Oaxaca:** km 27 on road from Teotitlán to Huautla, at Puerto Soledad, *D. Spooner 953a* (MEXU). **Puebla:** faldas del Ixtlacihuatl, F. Salazar 1914 (MEXU). **Querétaro:** 1 km aL NE de Pinal de Amoles, municipio de Pinal de Amoles, *A. Herrera 203* (ENCB, IEB, MEXU). **Tlaxcala:** atrás de las cabañas del CREA en Ixtenco, *V. Sánchez 297* (MEXU). **Veracruz:** Calcahualco, 9 km W of Escola, Nee 23,189 (F, XAL).

14. *Physalis crassifolia* Benth., Bot. Voy. Sulphur [Bentham] 40 (1844)

**Type:** Mexico, Baja California Sur, Lower California, Bay of Magdalena R.B. Hinds s.n. (lectotype designated by Pretz and Deanna 2020 K barcode K000042380!). (Figures 1I, 6B).

Synonyms in Pretz and Deanna (2020).

Perennial herb, sometimes woody-stemmed, 10–40 cm tall, variously vestite with short and glandular trichomes; petioles 1–4 (−6) cm long; blades ovate (1−) 2–6 (−10) cm long, 1–5 (−7) cm wide, apex acute to acuminate, base truncate to cordate, margins entire or toothed; flower solitary on peduncles 1–3.3 cm long; calyx with triangular lobes 2–3 mm long, corolla campanulate, rotate to funnelform, yellow, 8–30 mm long and 8–20 mm wide, immaculate, or with slightly darkened maculations; anthers yellow, 2.5–3.5 mm long; fruiting peduncles 2.2–7 cm long, sometimes coiling, fruiting calyx 10-costate, 1–6 cm long, 8–35 mm wide; mature berry green, spherical, 1 cm in diameter, with numerous brown seeds ca. 2 mm in diameter.

**FIGURE 7:**
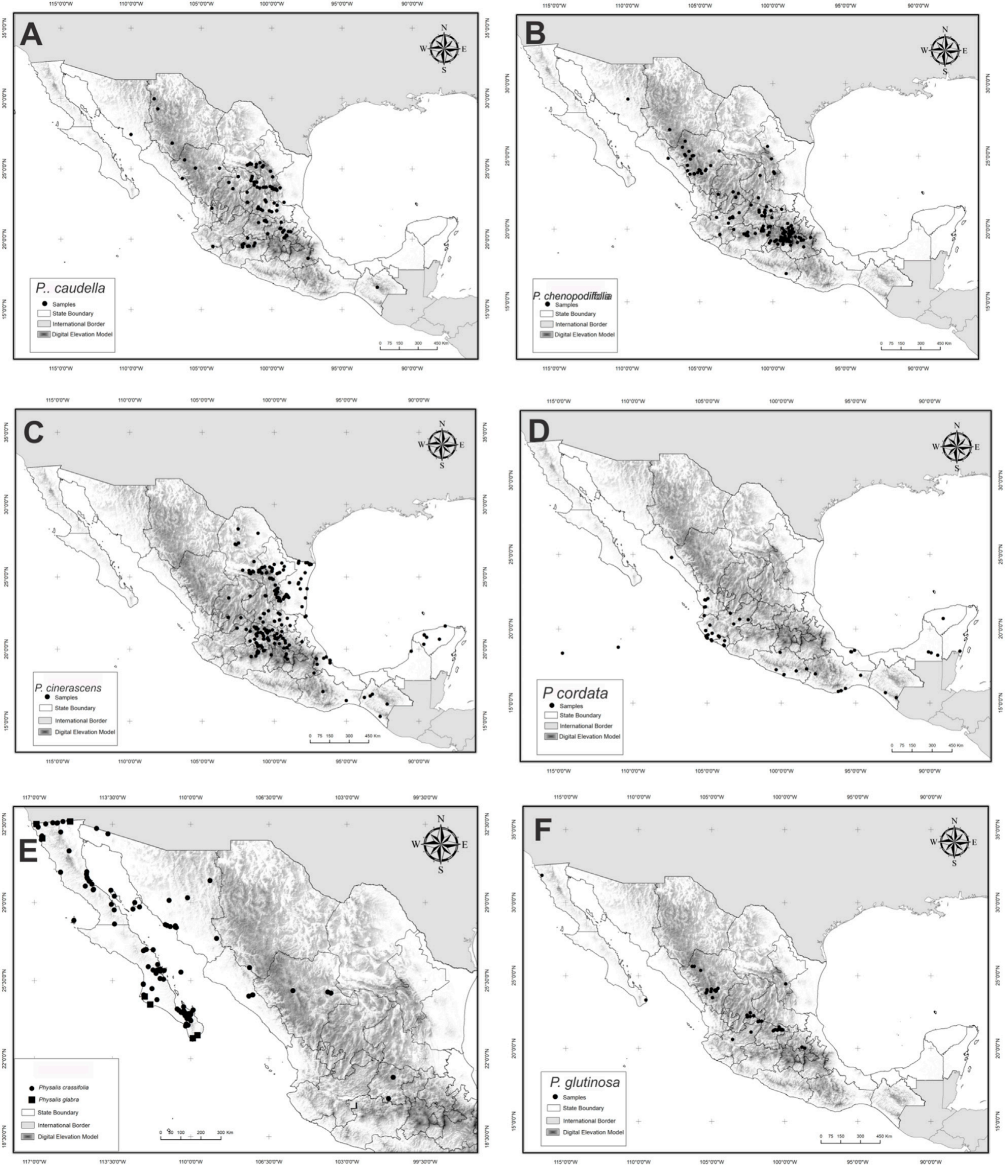
Geographic distribution of *Physalis spp*. **(A)** *P. caudella*, **(B)** *P. chenopodiifolia*, **(C)** *P. cinerascens*, **(D)** *P. cordata*, **(E)** *P. crassifolia* and *P. glabra*, **(F)** *P. glutinosa*.

**Distribution and habitat:** It grows in coastal dunes and sandy places, protected between rocks near cliffs and oases, from 0 to 200 m asl. B.C., B.C.S., Sin., Son. (Figure 7E).

**Diagnostic characters:** A perennial plant of desert areas, immaculate corollas that are sometimes funnelform, referred to as var. *infundibularis* I. M. Johnston.

**Common names and uses:** Tomatillo. No uses known.

**Phenology:** Flowering and fruiting throughout the year.

**Conservation status**: LC.

**Representative specimens examined.** MEXICO. **Baja California:** Cataviña, en el arroyo, *M. Martínez 9545* (QMEX). **Baja California Sur:** Comundu, poblado de Banderitas a 9.6 km al E de San Carlos, *M. Martínez 9524* (QMEX). **Sinaloa:** en Bagresitos, a 39 km al N de Culiacán, *E. Martínez 4122* (MEXU). **Sonora:** Pinacate Region Hourglass Canyon, two miles NE of Tinaja Huarache (=Pyramid Tank) on the west side of Sierra Pinacate, *R. Felger 19,175* (MEXU).

15. *Physalis glabra* Benth., Bot. Voy. Sulphur [Bentham] 39 (1844)

**Type:** Mexico, [Baja California Sur] Cape Lucas Hinds, s.n. 1841 (holotype: K specimen K000042387!)

= *P. hastata* Rydb. Mem. Torrey Bot. Club 4: 363 (1896). Type: Mexico, Baja California Sur, Todos Santos T. S. Brandegee 422 (holotype: UC not located, isotypes: GH not located, US catalog 00027327!)

Perennial herb, 10–100 cm long, glabrous or minutely puberulous; petioles 12–25 mm long, blades lanceolate 2–4 cm long, 0.7–3.0 cm wide, apex acute, base truncate to hastate, margin undulate with five to seven teeth; flowers solitary on peduncles 10–20 mm long; calyx with deltoid lobes ca. 2 mm long; corolla rotate, yellow, 9–20 mm in diameter, with blue slightly contrasting maculations; anthers yellow, 2.0–2.5 mm long; fruiting peduncles 17–30 mm long; fruiting calyx 10-costate, 10–23 mm long, 11–20 mm wide; mature berry spherical 10–12 mm in diameter, with numerous yellow seeds ca. 2 mm in diameter.

**Distribution and habitat:** Coastal dunes and oases, B.C.S. (Figure 7E).

**Diagnostic characters:** A glabrous woody perennial with small hastate leaves.

**Common names and uses**: None known.

**Phenology:** Flowering and fruiting from December to March.

**Conservation status**: LC.

**Representative specimens examined.** MEXICO. **Baja California Sur:** Los Cabos, *A. Anthony 353* (MEXU).

16. *Physalis glutinosa* Schltdl., Linnaea 19 (3): 310 (1846)

**Type:** Mexico, Hidalgo, Cuesta Blanca, Mineral del Monte, C. Ehrenberg 585 (there are two specimens deposited at HAL; we designate specimen barcode 042,219! as the lectotype, as it has more material and is better preserved). (Figures 1J, 3C, 6E).

= *Physalis cinerea* Waterf., Rhodora 69: 102 (1967). Type: Mexico, San Luis Potosí, in the region of San Luis Potosí, C. C. Parry & Ed. Palmer 649 (holotype: NY barcode 138,869!, isotype: GH 00077347!).

= *Physalis eximia* Standl., Fieldiana, Bot. 27: 273 (1937). Type: Mexico, Chihuahua, Majalca, D. H. LeSueur 894 (holotype: F barcode V0073000F!, isotypes: GH 00077351, UC not located).

= *Physalis glutinosa* var. *eximia* (Standl.) Waterf., Rhodora 69: 101 (1967).

Erect perennial herb, sometimes spreading, up to 1.5 m tall, branched, densely pubescent, glutinous, fetid; leaves alternate or geminate, petioles 1.2–4.7 cm long; blades ovate, subcordate or suborbicular, 3.3–8.2 cm long, 2.0–5.6 cm wide, apex acute to acuminate, base oblique, margin dentate, sinuate, or entire; flowers solitary on peduncles 1.5–2.0 cm long; calyx 1.1–1.7 cm long, 6–11 mm wide, corolla tubular campanulate, yellow or cream yellow, 22–35 mm long, 25–35 mm in diameter, with diffuse purple maculations; anthers purple, 4–5 mm long; fruiting peduncle 1.6–2 cm long, fruiting calyx 10-costate, reticulate, 2.4–4.2 cm long, 1.6–4.3 cm wide; mature berry spherical, 1.2–1.5 cm in diameter with very few (1–5) yellow seeds ca. 2.7 mm in diameter.

**Distribution and habitat:** An infrequent large plant that grows near xerophytic scrubs and dry oak forests at 2,000–2,500 m asl. Ags., Chih., Cd. Mex., Dgo., Gto., Hgo., Jal., Zac. (Figure 7F).

**Diagnostic characters:** A large plant, densely glutinous, pubescent, with large tubular-campanulate corollas.

**Common names and uses:** Tomatillo. No uses known.

**Phenology:** Flowering and fruiting from July to April, probably throughout the year.

**Conservation status**: LC.

**Representative specimens examined.** MEXICO. **Aguascalientes:** San José de Gracia, camino al cerro de La Ardilla, cuesta La Gloria*, De la Cerda García 730* (CIIDIR-DGO). **Chihuahua:** Majalca, *D. LeSueur 894* (F). **Ciudad de México:** Eslava, *E. Lyonnet 714* (MEXU). **Durango**: mountainside five miles southwest of Durango, *U. Waterfall 15,409* (MICH). **Guanajuato:** 4 km al S de Santa Bárbara, municipio de Ocampo, *E. Carranza y Col. 4314* (IEB, QMEX). **Hidalgo:** Metepec, *C. Pringle 13,125* (MEXU). **Jalisco**: Mezquitic, km 50 camino Bolaños-Tenzompa, al NE de Pinos Altos, comunidad indígena de Santa Catarina, *González Villarreal 3176* (CIIDIR-DGO, IBUG, IEB)**. Zacatecas:** 15 (air) miles NE of Estación Camacho on NW slopes near granitic summit of Pico de Teyra, *J. Henrickson 13,456* (MEXU).

17. *Physalis gracilis* Miers, Ann. Mag. Nat. Hist. ser. 2, 4 (19): 37 (1849)

**Type:** Mexico [Hidalgo] Real del Monte, T. Coulter 1222 (holotype: K barcode K000042379!, isotype: GH 00077352!). (Figures 2D, 3B, 6E).

Perennial herb, slightly erect to prostrate, up to 90 cm, rooting at the lower nodes, with short, eglandular, septate trichomes; leaves geminate throughout; petiole 8–15 mm long, blades ovate to sub-orbicular, 4.2–9.2 cm long, 2.1–5.2 cm wide, apex acute, base cuneate or oblique, narrowly decurrent, margin entire; flowers solitary on peduncles 1.3–2.5 cm long; calyx with deltoid to lanceolate lobes 3–4 mm long; corolla rotate-campanulate, yellow, 1.5–2.0 cm in diameter, with five reddish-brown maculations; anthers purple-blue, 3.0–3.5 mm long; fruiting peduncle 1.3–2.6 cm long, fruiting calyx 10-costate, 2.6–3.4 cm long, 1.1–2.1 cm wide; mature berry spherical, yellow, 8–10 mm in diameter with numerous yellow seeds ca. 1.5 mm in diameter.

**FIGURE 8:**
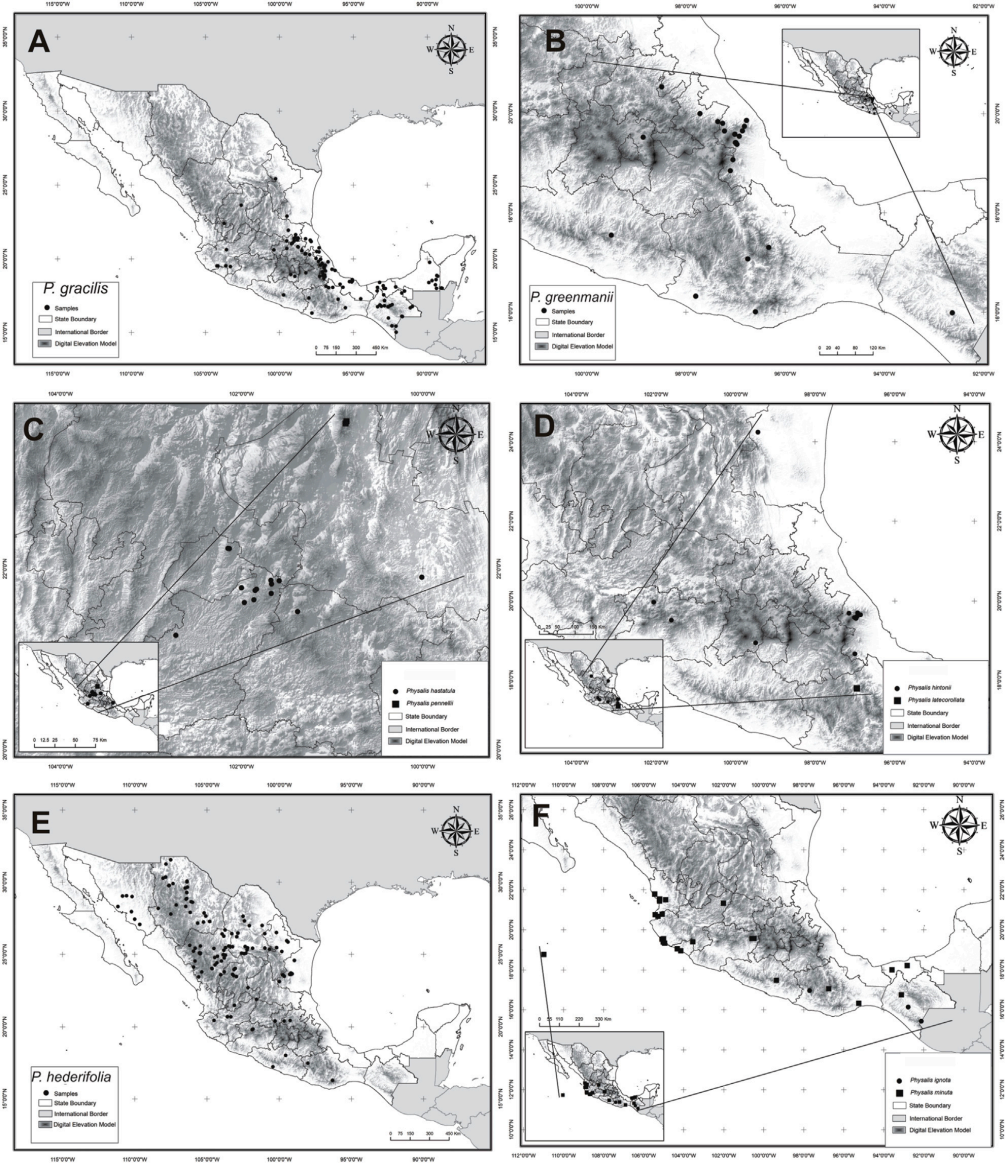
Geographic distribution of *Physalis* spp. **(A)** *P. gracilis*, **(B)** *P. greenmanii*, **(C)** *P. hastatula* and *P. pennellii*, **(D)** *P. hintonii* and *P. latecorollata*, **(E)** *P. hederifolia*, **(F)** *P. ignota* and *P. minuta.*

**Distribution and habitat:** Open areas of pine–oak or cloud forest, or as a weed in crops associated with the forest. 500–1,700 m asl. Camp., Chis., Hgo., Jal., Mor., Oax., Pue., Qro., Q. R., S.L.P., Tab., Tamps., Ver. (Figure 8A).

**Diagnostic characters:** A perennial with a prostrated habit, rooting at the basal nodes.

**Common names and uses:** Costomate, costomatillo, tomate, tomatillo, tomate borracho, tomatito amarillo, tomate de bolsa, miltomate de monte, chihol ch’o, chucky chan, moen’cho, chuchan. The sweet fruit is eaten fresh, and the plant is medicinal.

**Phenology:** Flowering and fruiting in July–September, probably throughout the year.

**Conservation status**: LC.

**Representative specimens examined.** MEXICO. **Campeche:** Calakmul, en Narciso Mendoza *D. Álvarez 17* (MEXU). **Chiapas:** Ocozocoautla de Espinosa, 1 km al N de Buenavista (El Suspiro) *E.* *Martínez 31,288* (MEXU). **Hidalgo:** Molango de Escamilla, Ismolintla, 5 km aL norte de Molango *R. Hernández 7094* (MEXU). **Jalisco:** Cuautitlán de García Barragán, Ayotitlán, cerro El Pinacate, 1/2 km adelante de san Miguel, *O. Vargas 805* (IBUG). **Morelos:** wet banks, barranca near Cuernavaca, *C. Pringle 6319* (MEXU). **Oaxaca:** San Lucas Ojitlán, del poblado El Zapotal a Mata Caña, *J. Calzada 14,314* (MEXU). **Puebla:** Xicotepec, 3 km adelante de Xicotepec de Juárez rumbo a Poza Rica, *M. Cházaro 562* (MEXU). **Querétaro:** en el pueblo de Concá en los nacimientos, municipio de Arroyo Seco*, M. Martínez 3456* (QMEX). **Quintana Roo:** Othón P. Blanco en Tomás Garrido, *E. Cabrera 17,013* (MEXU). **San Luis Potosí:** Xilitla, a 3 km aL NW de Huicihuayán, *P. Tenorio 63* (MEXU). **Tabasco:** Paraíso, *M. Magaña 20* (MEXU). **Tamaulipas:** Ejido Allende 30 km aL NW de Ocampo*, L. Hernández 1450* (MEXU). **Veracruz:** Rancho Guadalupe, 3 km W of Jalapa, *J. Calzada 1893* (MEXU, XAL).

18. *Physalis greenmanii* Waterf., Rhodora 69: 226 (1967)

**Type:** Mexico, Veracruz, Jalapa, C. G. Pringle 8104 (holotype: US barcode 00,027,326!, isotypes: F barcode V0073002F!, GH 00077353!, MEXU!, NY 138871!, UC 104116!, VT not located). (Figures 1K, 6D).

Perennial herb, 0.8–2 m long, weak and reclining on other vegetation; stem viscid-pilose with articulating trichomes; petiole 1.5–5.5 cm long, blades ovate, 5–10 cm long, 3.0–6.5 cm wide, apex acuminate, base cordate, margin entire or usually with one to two irregular, obtuse teeth per side; solitary flowers on peduncles 5–15 mm long; calyx 1.2–1.4 cm long; corolla rotate, pale yellow with five clusters of purple maculations, 15–28 mm wide, velvety-pubescent on the throat; anthers purple, 2.8–3.5 mm long; fruiting peduncles 7–10 mm long, fruiting calyx 5-angled; 3.0–4.5 cm long, 2.5–3 cm wide, mature berry spherical, ca. 1.5 cm in diameter, seeds ca. 1.8 mm in diameter.

**Distribution and habitat:** Cloud forest, 1,000–2,000 m asl., endemic to Ver. (Figure 8B).

**Diagnostic characters:** A perennial herb with cordate leaves, spotted maculations, corollas, and long purple anthers.

**Common names and uses:** None known.

**Phenology:** Flowering and fruiting from June to April, probably throughout the year.

**Conservation status**: EN.

**Representative specimens examined.** MEXICO. **Veracruz**: Xico, Cascada de Texolo, *P. Zamora-Tavares et al. 212* (IBUG).

19. *Physalis grisea* (Waterf.) M. Martínez, Taxon 42 (1): 104 (1993): (1993)

**Type:** USA, Massachusetts, Middlesex Co., Cambridge, W. Deane s.n. (holotype: GH barcode 00003293!, isotype: NY!). *Physalis pubescen*s var. *grisea* Waterf. Rhodora 60 (714): 167–168. 1958. (Figures 3I, 6H).

Erect annual herb, sericeous with non-glandular multicellular trichomes; petioles 5–7 cm long, blades ovate to lanceolate, 10–20 cm long, 5.0–5.4 cm wide, apex acute to acuminate, base oblique, margins coarsely dentate; flowers solitary on peduncles 4–6 mm long; calyx with triangular lobes 1.5–2.2 mm long, corolla rotate, yellow, 0.7–1.0 mm in diameter with simple dark purple maculations; filaments purple, anthers yellow or with a faint blue tinge, 1.1–2.0 mm long; fruiting peduncles 6–10 mm long, fruiting calyx strongly 5-angled, 2.5–3.0 cm long, 1.0–2.2 cm wide, sericeous throughout; mature berry yellow, spherical, 1.0–1.5 cm in diameter, with numerous brown seeds 1.5–2.0 mm in diameter.

**Distribution and habitat:** A spontaneous plant in disturbed areas. The plant is common in the United States but has been collected only recently in Mexico at 1,500 m asl in oak forest and crop fields. Jal., Mich.

**Diagnostic characters:** Annual, the leaves are orange-tinged.

**Common names and uses:** Tomatillo. The fruits are edible and mostly sweet.

**Phenology:** Flowering from May to October.

**Conservation status:** Not evaluated, but the species is widely distributed.

**Representative specimens examined.** MEXICO. **Jalisco:** Zapopan, cerro El Tepopote, frente al poblado de La Venta del Astillero, ladera de exposición este, *A. Rodríguez 6578* (IBUG, UAQ, MEXU). **Michoacán**: Sahuayo, áreas de cultivo de Sahuayo, *J. Sánchez 122* (IBUG, UAQ, MEXU).

20. *Physalis hastatula* Waterf., Rhodora 69: 111 (1967)

**Type:** Mexico, Jalisco, Cerro La Campana, near km 36 SW of Ojuelos on the road to Aguascalientes, R. McVaugh 16778 (holotype: MICH barcode 1000037!; isotypes: ENCB!, LL!, OKLA not located). (Figures 3E, 6F).

Prostrate perennial herb, branched from the base, glabrous or almost puberulent, with eglandular, short trichomes; leaves solitary or geminate, petiole up to 11 mm long; blades lanceolate to linear-lanceolate, often hastate, sometimes asymmetrical, 1–5 cm long, 0.7–2.4 cm wide, apex acute, base oblique, cuneate, decurrent, margin entire or with one to two teeth on both sides; flowers solitary on peduncles 6–10 mm long; calyx with narrow triangular lobes 3–5 mm long, corolla campanulate rotate, yellow, 0.8–2.1 cm in diameter, with simple dark brown to yellowish-brown maculations; anthers yellow, 2.0–2.5 mm long; fruiting peduncle 8–9 mm long, fruiting calyx 10-costate, with five more prominent angles, 1.4–2.5 cm long, 1.0–1.7 cm wide; mature berry green ca. 10 mm diameter, with yellow seeds ca. 1.8 mm in diameter.

**Distribution and habitat:** It develops in thorny forests with *Opuntia*, *Acacia*, and *Mimosa*, or grasslands on rhyolitic rocks or close to bodies of water in sandy places. It grows from 1,880 to 2,020 m asl. Ags., Gto., Jal., Zac. (Figure 8C).

**Diagnostic characters:** Hastate leaves, almost prostrated habit, and cinereal appearance of the plant.

**Common names and uses**: None known.

**Phenology:** Flowering and fruiting from August to September.

**Conservation status**: EN.

**Representative specimens examined.** MEXICO. **Aguascalientes:** Rincón de Romos, shrub-covered, nearly treeless mountainsides ca. 20 km east of Rincón de Romos, road to Asientos, between cerro Altamira and cerro San Juan, *R. McVaugh 23,758* (MICH). **Guanajuato:** cerca de la Quebrada, municipio de Ocampo, *J. Rzedowski 52,320* (IEB, QMEX). **Jalisco:** km 36 de la carretera Ojuelos-Lagos de Moreno, cercano al arroyo del poblado La Troje, *O. Vargas 771* (IBUG). **Zacatecas**: Pinos, Los Alpes, 200 m al E del poblado, *O. Vargas 769* (IBUG).

21. *Physalis hederifolia* A. Gray, Proc. Amer. Acad. Arts 10: 65 (1874)

**Type:** United States. New Mexico, Western Texas to El Paso C. Wright 528 (lectotype selected by Pretz and Deanna (2020) GH barcode 00077275!, isolectotypes: BM barcode BM 000995493, GH barcode 00077273, K barcode K759425, US barcode 01178071 right-hand specimen only). (Figures 1L, 6G). Synonyms in Pretz and Deanna (2020), except for *P. puberula* Fernald; see comments under *P. chenopodiifolia* Lam.

Perennial herb 60–80 cm high, branched from a woody base, pubescent with eglandular and short, glandular trichomes; leaves alternate, petioles 1.6–2.6 cm long; blades ovate to subcordate, 2.4–3.5 cm long, 2.1–3.0 cm wide, apex acute, base cuneate, oblique to truncate, margin serrated; flowers solitary on peduncle 5–8 mm long; calyx with ovate-to-triangular lobes 2–4 mm long, corolla campanulate rotate, yellow, 0.7–1.2 cm in diameter, with five pale maculations; anthers yellow, 3.5–4.0 mm long; fruiting peduncle 9–13 cm long, fruiting calyx 10-costate with five more prominent angles, 2.5–2.8 cm long, 1.7–2.2 cm wide, mature berry green to orange, ca. 9 mm in diameter, with numerous yellow seeds 1.6–1.8 mm in diameter.

**Distribution and habitat:** Desert areas with *Larrea* and *Flourensia*, disturbed places with calcareous soils. It grows from 1,400 to 1,500 m asl. Chih., Coah., Dgo., Gro., Jal., N.L., Oax., Qro., S.L.P., Son., Tamps., Zac. (Figure 8E).

**Diagnostic characters:** Densely pubescent, with almost subcordate leaves, short peduncles, and almost immaculate corollas, yellow anthers.

**Common names and uses**: None known.

**Phenology:** Flowering from April to June and fruiting in August.

**Conservation status**: LC.

**Representative specimens examined**. MEXICO. **Chihuahua:** pasando San Juditas hacia San Buenaventura, *M. Martínez 9641* (QMEX). **Coahuila:** Las Margaritas, km 190 carretera San Pedro-Cuatro Ciénegas, *M. Martínez 9616* (QMEX). **Durango:** Tayoltita: El Pino, 20 km del entronque a Sapioris con la brecha Coyotes-san Miguel de Cruces, 24° 31′ N, 105° 49′ W, *P*. *Tenorio 6318* (MEXU). **Guerrero:** Zihuatanejo de Azueta en El Bálsamo, *J. Soto 8660* (MEXU). **Jalisco**: Ixtlahuacán del Río, puente Paso de Guadalupe, *Carrillo Reyes 3590 et al.* (IBUG). **Nuevo León:** 3 km al S de la desviación a Cerralvo, camino a Los Herrera, *M. Martínez 9819* (QMEX). **Oaxaca:** en las ruinas de Monte Albán entre los edificios, en lugares húmedos, *M. Martínez 1903* (MEXU). **Querétaro:** km 203 carretera a México*, E. Argüelles 781* (MEXU). **San Luis Potosí:** Salinas, gravelly desert along mex. 49, 33.4 miles E of Salinas (ENCB). **Sonora:** Naco, desviación a Naco, de la carr, Cananea-Agua Prieta, *P. Tenorio 13,622* (MEXU). **Tamaulipas:** km 5 de la carretera vía corta a Ciudad Victoria, 31 km aL NE de Jaumave, *M. Martínez 9890* (QMEX). **Zacatecas**: Melchor Ocampo, near and at Sierra del Yeso, almost due west of La Presa de los Angeles, 25° 04′ N, 102° 08′ 30″ W, *Johnston 11,531* (MEXU).

22. *Physalis hintonii* Waterf., Rhodora 69: 226 (1967)

**FIGURE 9:**
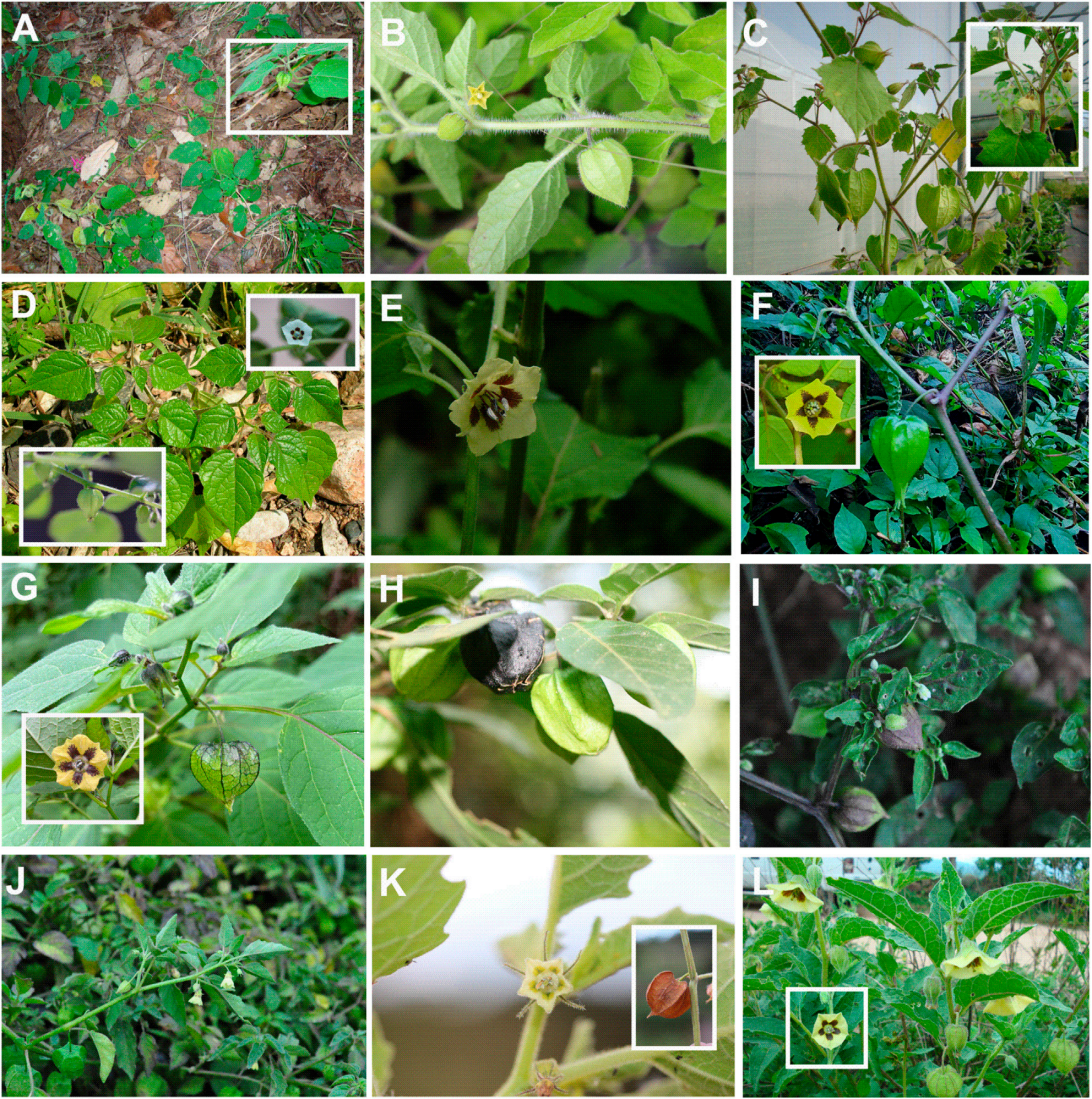
Details of flowers and fruits of *Physalis* spp. **(A)** *P. hintonii*, **(B)** *P. lagascae*, **(C)** *P. latiphysa*, **(D)** *P. leptophylla*, **(E)** *P. lignescens*, **(F)** *P. longiloba*, **(G)** *P. longipedicellata*, **(H)** *P. melanocystis*, **(I)** *P. microcarpa*, **(J)** *P. minuta*, **(K)** *P. nicandroides*, **(L)** *P. orizabae.*

**Type:** Mexico, Estado de Mexico, Tejupilco, Dist. of Temascaltepec, G. B. Hinton 8457 (holotype: NY barcode 477364 !, isotypes: F 1498378!, CAS 4031!, ENCB not located, GBH not located, GH 00077354, OKLA not located, US barcode 00027332!). (Figure 9A).

Erect perennial herb, 30–120 cm tall; stem with few branches with dense dendritic trichomes up to 1 mm long throughout; basal leaves geminate, petioles 10–35 mm long, blades ovate, 4–9 cm long, 4–5 cm wide, apex acute, base truncate to cordate, margin undulate to dentate; solitary flowers on peduncles 2.5–5.0 cm long; calyx with deltoid lobes 5–10 mm long, corolla campanulate-rotate, 17–20 mm wide, with five dark brown maculations; anthers violet, 3–4 mm long; fruiting peduncle 10–16 mm long, fruiting calyx 10-costate, open at the apex, 25–35 mm long and 18–22 mm wide; mature berry ca. 1 cm in diameter with numerous yellow seeds ca. 1 mm in diameter.

**Distribution and habitat:** Pine–oak forest; it grows at 1,200–2,100 m asl. Edo. Mex., Mich., Ver. (Figure 8D).

**Diagnostic characters:** *P. hintonii* is easily recognized by its spreading dendritic (several-branched) trichomes and its large flowering calyx with broad sepals.

**Common names and uses**: None known.

**Phenology:** Flowering and fruiting from March to July.

**Conservation status**: LC.

**Representative specimens examined.** MEXICO. **Estado de México**: Tenancingo, al O de Exhacienda San José Tenería, *P. Zamora-Tavares et al. 272* (IBUG)**. Michoacán:** Presa la Yerbabuena, municipio de Tlazazalca, *E. Pérez 1600* (IEB, QMEX). **Veracruz**: Jilotepec, Piedra de Agua, *F. Ventura 9820* (ENCB, F, XAL).

23. *Physalis hunzikeriana* M. Martínez, Kurtziana 27 (2): 383 (−385, 1999)

**Type**: Mexico, Nuevo León, Cerro el Viejo, J. Hinton et al., 22104 (holotype: GBH!).

Erect perennial herb 30–100 cm high, stems with eglandular and glandular trichomes mixed with dendritic; leaves alternate, petioles 1.5–5.0 cm long; blades ovate, 6–11 cm long, 4.5–5.5 cm wide, apex acute to acuminate, base rounded to attenuate, margin dentate with three to six teeth per side, pubescent with glandular and dendritic trichomes; flowers solitary on peduncles 5–8 mm long; calyx with lanceolate lobes 10 mm long, corolla rotate, yellow, 22–25 mm in diameter with five purple maculations, corolla throat densely pubescent; anthers blue or yellow with blue lines 2.5–4 mm long; fruiting peduncle 20–30 mm long, fruiting calyx 5-angled, 35–50 mm long, 25–30 mm wide; mature berry spherical, 20 mm in diameter, with numerous yellow seeds ca. 2 mm in diameter.

**Distribution and habitat:** Pine–oak forest from 1,600 to 2,270 m asl. N.L.

**Diagnostic characters:** A perennial herb with a mixture of eglandular, glandular, and dendritic trichomes throughout the plant.

**Common names and uses**: None known.

**Phenology:** Flowering and fruiting in July; no other collection dates are known.

**Conservation status:** DD; the plant is known only from one mountain range.

**Representative specimens examined.** MEXICO. **Nuevo León:** municipio de Zaragoza, cerro El Viejo, *J. Hinton 22,255* (Herb. Hinton).

24. *Physalis ignota* Britton, Mem. Torrey Bot. Club 16: 100 (1920)

**Type:** Cuba Santa Clara Rio Arimao, Britton and Wilson 5767 (holotype: NY barcode 620785!).

= *Physalis pentagona* S.F.Blake, Contr. U.S. Natl. Herb. 24: 20 (1922). Type: Guatemala, Izabal, Los Amates, S.F.Blake 7313 (holotype: US barcode 00027352!)

Erect annual herb, 10–100 cm high, usually densely covered with short glutinous, multicellular trichomes throughout; petioles 2–7 cm long, blades ovate to rhombic-ovate, 4–12 cm long, 3–8 cm wide, apex acuminate, base oblique to cordate, margin entire to repand-dentate; flowers solitary on peduncles 3–7 mm long; calyx with triangular lobes; corolla campanulate, yellowish, 6–10 mm in diameter, sparsely hairy in the throat, immaculate; anthers yellow or bluish tinged, 2.0–2.5 mm long; fruiting peduncle 7–15 mm long, fruiting calyx strongly 5-angled, 2.8–5 cm long, 2–4 cm wide, usually densely and evenly covered with short, erect, jointed trichomes; mature berry spheric to ovoid, 9–15 mm in diameter with numerous yellow seeds ca. 1.5 mm in diameter.

**Distribution and habitat:** In wet or moist scrub, sometimes on stony or rocky slopes from sea level to 1,250 m. Chis., Oax. (Figure 8F).

**Diagnostic characters:** 5-angled fruiting calyces, the stem, peduncles, and fruiting calyx densely pubescent with gray, short trichomes.

**Common names and uses:** Vejiga de perro, tomatillo, soplón, farolito. No uses known.

**Phenology:** Flowering and fruiting throughout the year.

**Conservation status**: LC.

**Representative specimens examined.** MEXICO. **Chiapas:** Amatenango de la Frontera, steep slopes and dry ravines along Río Cuilco between Nuevo Amatenango and Frontera Comalapa *D. Breedlove 41,575* (MEXU). **Oaxaca:** Santo Domingo Tehuantepec, Llano de Lumbre, Santiago Lachiguirí, Jalapa del Marqués *E. Cruz 97a* (MEXU).

25. *Physalis lagascae* Roem. & Schult., Syst. Veg., ed. 15 bis [Roemer & Schultes] 4: 679 (1819)

**Type:** based on *P. parviflora* Lagasca. (Figures 3D, 9B).

= *Physalis lagascae* var. *glabrescens* O.E.Schulz, Symb. Antill. (Urban). 6 (1): 147 (1909). Type: Cuba, provincia de la Habana prope Calabazar, Wright 3636, (holotype: US barcode 00027335!)

= *Physalis parviculea* S.F.Blake, Contr. U.S. Natl. Herb. 24: 20 (1922). Type: Guatemala, Izabal, Los Amates, S.F.Blake 7318 holotype: US barcode 00027348!)

Erect annual herb 15–70 cm tall; stem branched, with adpressed whitish trichomes; leaves geminate, petioles 1.3–4.6 (–8) cm long; blades ovate to suborbicular ovate, 2.6–7.0 cm long, 1.3–4.2 cm wide, apex acuminate, base oblique to subcordate, margin entire to sinuate lobed; flowers solitary on peduncle 3–8 mm long; calyx with ovate lobes 1.5–2.5 mm long, corolla campanulate, rotated to pentagonal, yellow, 6–8 mm in diameter, with five almost black maculations; corolla throat pubescent; anthers yellow or blue 2 mm long; fruiting peduncle ca. 4 mm long, fruiting calyx 10-costate, 1.2–2.0 cm long, 1.2–2.0 cm wide; mature berry ca. 10 mm diameter with numerous yellow seeds ca. 2 mm in diameter.

**FIGURE 10:**
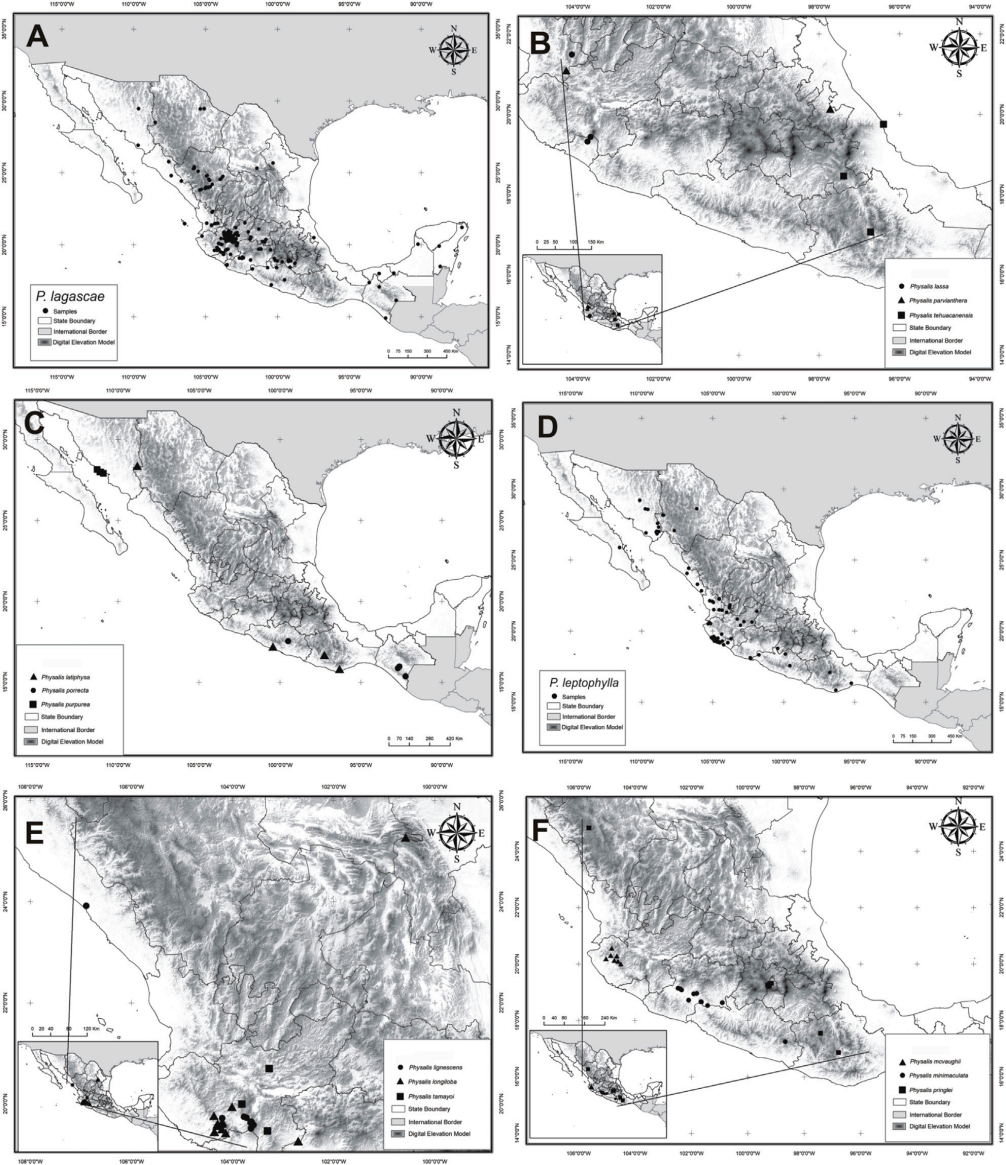
Geographic distribution of *Physalis* spp. **(A)** *P. lagascae*; **(B)** *P. lassa*, *P. parvianthera*, and *P. tehuacanensis*; **(C)** *P. latiphysa*, *P. porrecta*, and *P. purpurea*; **(D)** *P. leptophylla*; **(E)** *P. longiloba*, *P. lignescens*, and *P. tamayoi*; **(F)** *P. mcvaughii*, *P. minimaculata*, and *P. pringlei.*

**Distribution and habitat:** The species is generally found in secondary vegetation as a weed, in grasslands, or associated with tropical deciduous forest. It grows from 0 to 2,100 m asl. Ags., Camp., Chis., Chih., Col., Dgo., Edo. Mex., Gto., Gro., Jal., Mich., Mor., Nay., N.L., Oax., Pue., Qro., Q. Roo, Sin., Tab., Ver., Yuc., Zac. (Figure 10A).

**Diagnostic characters:** Densely pubescent at the stem base. The species is easily recognized by the presence of eglandular, white conspicuous trichomes in flower buds and calyces, as well as small corollas with contrasting dark, triangular maculations.

**Common names and uses:** Tomatillo, vejiga de perro, chimpululu. No uses known.

**Phenology:** Flowering and fruiting from June to December.

**Conservation status**: LC.

**Representative specimens examined.** MEXICO. **Aguascalientes:** 6.5 km aL SW de Tanque de Los Jiménez, *J. Sierra 881* (QMEX). **Campeche:** en las Palomas, al sur de Candelaria, sobre el Río Candelaria, *E. Cabrera 4809* (MEXU). **Chiapas:** *D. Breedlove 26,384* (MO). **Chihuahua**: Temósachic, Nabogame, *J. LaFerrière 1551* (MEXU). **Colima:** Comala: Noguera, más o menos 13 km aL norte de Comala, *M. Arreguín 754* (MEXU). **Durango:** 5 km aL NW de Yerbanis, km 128 carretera Yerbanís-Cuencamé, *M. Martínez 9782* (QMEX). **Estado de México:** Temascaltepec, Pantoja, *G. B. Hinton 8606* (MEXU). **Guanajuato:** Abasolo: 35 km aL SW de Cuerámaro, sobre el camino a la Barranca del Chilar, *J. Rzedowski 45,015* (CIIDIR-SGO, ENCB, MICH). **Guerrero:** Rincon de la Via, *H. Kruse 541* (MEXU). **Jalisco:** Tala, márgenes del arroyo Caliente, 100 m antes de su nacimiento, Bosque Escuela, La Primavera, *A. Rodríguez 1471* (IBUG, IEB, MEXU)**. Michoacán**: Apatzingán, 6 km aL noroeste de Apatzingán, *O. Vargas 853* (IBUG). **Morelos:** Amacuzac, noroeste de Amacuzac, *L. Estrada 1295* (MEXU). **Nayarit**: La Yesca, La Manga, *Bugarín s. n*. (CHAPA, IBUG). **Nuevo León:** Mt. Obispado, *J. Roybal 1071* (MEXU). **Oaxaca:** Yomillin Cañon, *C. Pringle 7004* (MEXU). **Puebla:** Matamoros, *F. Miranda 2390* (MEXU). **Querétaro**; San Juan del Río, km 175.5 carretera Querétaro-México en la desviación a San Clemente, *M. Martínez 2990* (QMEX). **Quintana Roo**: a 18 km al W de Ucum, S de E de Nachi Cocom, *O. Tellez 2412* (MEXU). **Sinaloa:** Culiacán, Tierras Blancas 2 km al N de El Rincón, brecha a Tamazula, *P. Tenorio 8299* (MEXU). **Tabasco:** centro por el km 11 de Villahermosa a Cardenas, *M. Magaña 1918* MEXU). **Veracruz:** Coatzintla, Palamar de Zapata, *Cortés 125* (MEXU). **Yucatán:** Hacienda Cancabchen *A. Mizrahi s. n.* (UADY). **Zacatecas**: Jalpa, pastured hills five miles southwest of Jalpa, *R. McVaugh 18,513* (MICH).

26 *Physalis lassa* Standl. & Steyerm., Publ. Field Mus. Nat. Hist., Bot. Ser. 23: 19 (1943)

**Type:** Guatemala, Dept. Jalapa, grassy thickets, between Jalapa and Montaña Miramundo, J. Steyermark 32868 (holotype: F barcode 0077205F!).

Erect to prostrate perennial herb, branched, up to 50 cm tall, densely pubescent with eglandular septate trichomes mixed with some glandular, especially on the fruiting calyx; leaves alternate to geminate, petioles 1.0–3.8 cm long; blades ovate to suborbicular ovate, 3.5–7.5 cm long, 1.7–4.9 cm wide, apex acute to obtuse, base oblique to truncate, margin entire to sinuate or with few teeth; flowers solitary on peduncles 5–7 mm long; calyx with triangular lobes ca. 3 mm long, corolla rotate-campanulate, yellow, 10 mm in diameter, with five very dark purple maculations; anthers blue, 2.5–3.0 mm long; fruiting peduncle 0.9–1.7 cm long, fruiting calyx 10-costate, 2.6–3.5 cm long, 1.9–2.2 cm wide, pubescent; mature berry 1.0–1.5 cm in diameter with numerous yellow seeds ca. 2 mm in diameter.

**Distribution and habitat:** It grows at the margins of the mountain–cloud forest at an altitude of 1,500 m. Chis., Col. (Figure 10B).

**Diagnostic characters:** Corolla large, with contrasting maculations, large fruiting calyx. The long, glandular, dense pubescence gives *P. lassa* a wooly appearance with reddish-brown coloration.

**Common names and uses:** Tomatillo; no uses known.

**Phenology:** Flowering and fruiting in November.

**Conservation status**: LC.

**Representative specimens examined.** MEXICO. **Chiapas:** La Grandeza, *E. Matuda 1556* (MEXU). **Colima**: Comala, rancho El Jabalí, 20 km (airline) N of Colima in the SW foothills of the Volcán de Colima, El Agostadero, ridgetop in hills above the shrine 4.9 km SE of Hac. San Antonio on the road to Comala, *J. Sanders 11,807* (MEXU, MICH).

27. *Physalis latecorollata* Waterf., Rhodora 69: 117 (1967)

**Type:** Mexico, Oaxaca, orilla de arroyo, campamento Rio Molino cerca de San Miguel Suchitepec, Garcia s.n. (holotype: ENCB!).

Perennial herb, 30 cm tall; stems villous, with articulated trichomes up to 2–3 mm long; petioles 15–25 mm long; blades ovate, 5–6 cm long and 3.8–4.2 cm wide, apex acute, base cuneate, margin entire; flowers solitary on peduncle 5–7 mm long; calyx 8–9 mm long and 7–8 mm wide; corolla 33 mm in diameter with violaceous maculations; anthers purple, ca. 3 mm long; fruiting calyx, mature berry and seeds unknown.

**Distribution and habitat:** Riparian vegetation, 2,500 m asl. Oax. (Figure 8D).

**Diagnostic characters:** The species is only known from the type specimen, which lacks fruits. Its woody rhizome, pubescence, and the shape of the leaves distinguish it from *P. orizabae.*

**Common names and uses**: None known.

**Phenology:** Fflowering in September.

**Conservation status:** DD.

**Representative specimens examined.** MEXICO. **Oaxaca:** orilla de arroyo, campamento Rio Molino cerca de San Miguel Suchitepec, *Garcia s.n*. (ENCB).

28. *Physalis latiphysa* Waterf., Rhodora 60: 169 (1958).

**Type:** USA, Arizona. Pima County. Ronstadt Ranch, plain east of Baboquivari Mountains, T.H. Kearney & R.H. Peebles 14425 (holotype: ARIZ catalog 90142!, isotype: OKLA 101862). (Figures 1M, 9C).

Annual herb, 15–200 cm tall, branched, more-or-less villous or glandular villous; petioles 1.5–7.0 cm long; blades ovate, 5–7 cm long, 3–5 cm wide, apex acuminate, base truncate, sometimes oblique, margin entire to few-toothed; flowers solitary on peduncles 3–8 mm long; calyx with narrowly lanceolate to acuminate lobes, corolla rotate, yellowish 3–4 mm in diameter with five small, dark maculations; fruiting peduncles 1.0–1.5 cm long, fruiting calyx strongly 5-angled, sparsely appressed-hairy, 2.5–4 cm long, 3–4 cm wide; mature berry brown, 13–17 mm in diameter with numerous dark brown seeds ca. 2 mm diameter.

**Distribution and habitat:** It grows in tropical deciduous forest at an altitude of 1,500–2,000 m. Gto., Mich., Son. (Figure 10C).

**Diagnostic characters:** It is distinguished from other species that have linear-subulate calyx lobes by its corollas with dark maculations and fruiting peduncles longer than those of *P. nicandroides*, but shorter than those of *P. pruinosa.*

**Common names and uses**: None known.

**Phenology:** Flowering and fruiting from May to November and probably throughout the year.

**Conservation status:** LC.

**Representative specimens examined.** MEXICO. **Guanajuato:** 4 km al N de Uriangato, municipio de Uriangato, *J. Rzedowski 51820* (IEB, QMEX). **Michoacán:** 7 km al E de Villa Jiménez, sobre el camino a Copándaro, *J. Rzedowski 40745* (IEB, QMEX). **Sonora:** 19 km al E de Yécora, orillas de la carretera, *M. Martínez 9704* (QMEX).

29. *Physalis leptophylla* B. L. Rob. & Greenm., Proc. Amer. Acad. Arts 29: 389 (1894)

**Type:** Mexico, Sinaloa, near Mazatlán, Wright 1252 (lectotype designated by D’Arcy, Ann. Missouri Bot. Gard. 60: 6641973 GH barcode 00003285!). (Figure 9D).

Annual herb, branched, spreading, up to 1 m tall, with eglandular, brown glandular trichomes; leaves alternate, petioles 0.9–3.8 (−7.2) cm long; blades ovate to suborbicular ovate, 2.7–8.0 cm long, 1.8–4.8 cm wide, apex acuminate, base oblique, margin entire; flowers solitary on peduncles 4–5 mm long; calyx with ovate lobes 3–4 mm long, corolla campanulate to rotate, whitish to pale yellow, 0.7–1.4 cm in diameter, with five simple, reddish-brown to dark purple-brown maculations; anthers blue, 1.5–2.5 mm long; fruiting peduncle 6–8 mm long, fruiting calyx 10-costate, 1.5–2.5 cm long, 1.4–1.7 cm wide, green, translucent when dry; mature berry spherical, 4–9 mm in diameter, with numerous yellow seeds ca. 1 mm in diameter.

**Distribution and habitat:** Tropical deciduous and dry oak forest along the Mexican Pacific coast from 50 to 800 m asl. B.C.S, Chih., Col., Dgo., Edo. Mex. Gro., Jal., Mich., Nay., Oax., Sin., Son. (Figure 10D).

**Diagnostic characters:** Entire leaves, acuminate and translucent when dry, small dark maculations, contrasting with the corolla, and calyx 10-costate in fruit. The pubescence is glandular.

**Common names and uses:** Tomate, tomatillo, tomate del monte, shiquipiltzi. The ripe fruits are cooked in Sonora.

**Phenology:** Flowering and fruiting from October to February.

**Conservation status**: LC.

**Representative specimens examined.** MEXICO. **Baja California Sur**: 2 km aL NE de Misión de San Javier, km 32 de la carretera, *M. Martínez 9966* (QMEX). **Chihuahua**: camino Ocampo-Moris, km 48.5, *M. Martínez 9663* (QMEX). **Colima:** Manzanillo, steep hills with occasional rock outcrops, *ca.* 14 miles west-north-west of Santiago, road to Cihuatlán, Jalisco, *R. McVaugh 20,782* (ENCB, MICH). **Durango:** 10 mi N of Tamazula, *A. Gentry 5270* (CAS, NY). **Estado de México:** Temascaltepec, at Bejucos, *G. Hinton 5204* (US). **Guerrero:** Leonardo Bravo 14 km camino Chichihualco-Filo de Caballos, *J. Calónico 4395* (MEXU). **Jalisco:** Puerto Vallarta, along the highway in the gorge of the Rio Horcones, south of Puerto Vallarta, about 11 km from Puerto Vallarta, *Dieterle 4083* (ENCB)**. Michoacán**: Lázaro Cárdenas: steep hills about 45–48 km south of Arteaga, 12–15 km north of Playa Azul, *McVaugh 22,605* (ENCB, MICH). **Nayarit:** La Yesca, Paso de los Bueyes, río Santiago, 12 km al E de Mojarras, brecha a Huajimic, 21° 29′ N, 104° 32′ W, *P*. *Tenorio 6841* (MEXU, MICH)**. Oaxaca:** Arroyo de Piedra ladera W, entrando por el Mármol, Cerro Guiengola. Distr. Tehuantepec, *L. Torres 233* (MEXU). **Sinaloa:** Concordia, El Cantil 32 km aL NE de Concordia, carretera Mazatlán-Durango, *P. Tenorio 2957 (*MEXU). **Sonora:** Río Mayo Region arroyo Las Rastras, southwest edge of the Sierra de Alamos, *T. Van Devender 95-134A* (ASU).

30. *Physalis lignescens* Waterf., Rhodora 69: 231 (1967)

**Type:** Mexico: Jalisco, NE slopes of the Nevado de Colima, below Canoa de Leoncito, R. McVaugh 13454 (holotype: OKLA 100185, isotypes: MICH barcode 1109905!, US barcode 00027336, MEXU barcode 114184!). (Figure 9E).

Erect perennial herb up to 50 cm and then becoming prostrate up to 1 m long, with few branches, often leans on other plants; pubescent with eglandular articulated trichomes of different lengths; leaves alternate at the base, geminate toward the apex, petiole 1–3.4 cm long; blades ovate to suborbicular ovate, 4.5–9.0 cm long, 2.4–6.1 cm wide, apex acute to short acuminate, base oblique, decurrent, or cuneate, margin entire, repand, or with several acute, irregular teeth; flowers solitary on peduncles 8–17 mm long; calyx 4–5 mm long, corolla campanulate rotate, yellow, 1.5–2.1 cm in diameter, with brown compound maculations; anthers purple 2.5–3.5 mm long; fruiting peduncle 0.8–1.7 cm long, fruiting calyx 5-angled 2.0–4.1 cm long, 1.4–2.6 cm wide, reticulate, pubescent only on the lobes; mature berry 6–12 mm in diameter, yellow seeds, 2–2.5 mm in diameter.

**Distribution and habitat:** Abundant along the paths and margins of pine–oak and cloud forest, in clearings or ravines. It grows between 2,000 and 2,300 m asl. Endemic to Parque Nacional Nevado de Colima, Jal. (Figure 10E).

**Diagnostic characters:** prostrate habit, lignified base, short flower calyx with reflexed lobes, corolla with compound maculations and 5-angled fruiting calyx.

**Common names and uses**: None known.

**Phenology:** It flowers and bears fruit abundantly from July to August. Later, it is found with flowers or scarce fruits.

**Conservation status:** EN.

**Representative specimens examined.** MEXICO. **Jalisco:** Venustiano Carranza: puerto El Floripondio, camino a la estación de Microondas Las Víboras, *A. Rodríguez 913* (ENCB, IBUG, MEXU).

31. *Physalis longiloba* O. Vargas, M. Martínez & Dávila. Brittonia 53 (4): 505–507 (2001)

**Type:** Mexico. Jalisco: Autlán de Navarro, Sierra de Manantlán, passing Rincón de Manantlán along the creek, 19 35′55″ N, 104 12′35″ W, O. Vargas 873 (holotype: IBUG!, isotypes: MEXU!, NY barcode 468345!, ENCB!, IEB!, ZEA!). (Figure 9F).

Prostrate perennial herb, 1 m long, branched, glabrescent or with eglandular, short, appressed, puberulent trichomes; leaves alternate at the base but soon geminate, petiole 1.5–3.6 (−4.6) mm long; blades ovate to suborbicular ovate, 3.6–7.9 (–10.2) cm long, 2.1–6.1 cm wide, apex acuminate, base oblique, cuneate to subcordate, margin entire, wavy, or toothed; flowers solitary on peduncles 6–15 mm long; calyx lobes 5.5–9.0 mm long; corolla rotate campanulate, reflected, yellow, 1.5–2.2 cm in diameter, with compound maculations, brown, corolla throat pubescent; anthers purple, blue when dry, 3–4 mm long; fruiting peduncle 0.8–1.4 (−2.2) cm long, fruiting calyx 5-angled, 3.0–4.2 cm long, 1.3–2.0 cm wide, puberulent; mature berry ca. 11 mm in diameter with yellow seeds 1–1.5 mm in diameter.

**Distribution and habitat:** It grows on slopes, open areas, or paths in cloud forest, very humid pine–oak forest, or near bodies of water; abundant at 2,100 m. Jalisco (endemic). Known only from the Sierra de Manantlán, Jal. (Figure 10E).

**Diagnostic characters:** The shape and length of the floriferous calyx lobes and the 5-angled calyx in fruit, which reaches a large size, in peduncles smaller than 2 cm.

**Common names and uses**: None known.

**Phenology:** It flowers and bears fruit from September to May.

**Conservation status**: LC.

**Representative specimens examined.** MEXICO. **Jalisco:** Autlán de Navarro, predio Las Joyas, cerca del letrero del Sendero ecológico, *O. Vargas 862* (IBUG, MEXU, ENCB).

32. *Physalis longipedicellata* Waterf., Rhodora 69: 230 (1967)

**Type:** Mexico: Jalisco, Cuautitlán de García Barragán, Sierra de Manantlán near Aserradero El Cuartón, 15–20 miles SE of Autlán, R. McVaugh 13828 (holotype: MICH barcode 1109904!, isotypes: BRIT 23921!, G barcode G00343149!, K barcode K000042281, OKLA not located, MEXU!, US barcode 00027337!). (Figure 9G).

Erect perennial herb, or with the main stem prostrate when it reaches dimensions of about 2 m long, pubescent with eglandular, short, white trichomes; leaves geminate, petioles 3.6–6.7 (−9.2) cm long; blades sub-orbicular ovate, 8.5–16.5 cm long, 6.6–11 cm wide, apex acute to acuminate, base oblique, cuneate to subcordate, decurrent, winged, with a central groove where numerous trichomes accumulate, margin entire, sinuate, or with some irregular, shallow, acute teeth; flowers solitary on peduncles 1.7–4.5 cm long; calyx 8–11 mm long, 6–8 mm wide at the base of the triangular acuminate to ovate-deltoid lobes; corolla rotate campanulate, yellow, 2.2–2.4 cm in diameter, with dark purple or brown compound maculations; anthers purplish-blue, 2.0–3.5 mm long; fruiting peduncle (1.7) 4–6 cm long, fruiting calyx 5-angled, 3.3–4.0 cm long, 2.0–2.5 cm wide, very large for the berry; mature berry 1–1.2 mm in diameter.

**Distribution and habitat:** It develops in protected places, on slopes or margins of the cloud forest. The populations are small, with only one to three plants found at each location. It grows at an altitude range of 2,100–2,200 m. Jalisco (endemic). Known only from the Sierra de Manantlán.

**Diagnostic characters:** Its prostrate habit and large plant size. The leaves with long, glossy acumens and the long flowering and fruiting peduncles, as well as the wide corollas with compound maculations and the 5-angled calyx in the fruit.

**Common names and uses**: None known.

**Phenology:** It flowers and bears fruit from September to April.

**Conservation status**: LC.

**Representative specimens examined.** MEXICO. **Jalisco**: Cuautitlán de García Barragán, Sierra de Manantlán, 500 m antes de llegar a Tierras Blancas, por el camino a Llanos de San Miguel, *O. Vargas 868* (IBUG)

33. *Physalis mcvaughii* Waterf., Rhodora 69: 104 (1967)

**Type:** Mexico, Jalisco, two to three miles NW of San Miguel de la Sierra, R. McVaugh 22,034 (holotype: MICH barcode 1109903! isotypes LL not located, NY barcode 138876!).

= *Physalis jaliscensis* Waterf., Rhodora 69: 231 (1967). Type: Mexico, Jalisco, steep mountainsides and barrancas one to two miles N of sawmill “La Cumbre” on the divide above the headwaters of Rio Mascoto [Mascota], 25–30 km SE of Talpa de Allende, R. McVaugh 21512 (holotype: MICH barcode 1109906!, isotypes: ENCB!, OKLA not located, US barcode 00027334!).

Shrub up to 2 m tall, glabrous or glabrescent, eglandular trichomes; leaves alternate or geminate, petiole 1.5–5.5 cm long; blades ovate to rhombic-ovate, 7–13 cm long, 4–9 cm wide, the apex gradually attenuated, base oblique, decurrent, margin entire or with some teeth; solitary flower on peduncle 2.5–3 cm long; calyx 8–15 mm long and 7–8 mm wide, deltoid lobes 3–5 mm long; corolla campanulate, yellow, 1.1–1.4 cm long, 2.0–2.5 cm in diameter, compound maculations, brown; anthers violaceous, 2.7–3.5 mm long; fruiting peduncle 4.0–5.5 cm long, fruiting calyx 10-costate, 2.5–4.5 cm long, 2.3–3.5 cm wide; mature berry 1.5–2 cm in diameter.

**Distribution and habitat:** It grows in cloud mountain forest at 1,700–2,000 m. Jalisco (endemic). It is known only from the Sierra de Manantlán and Cacoma (Figure 10F).

**Diagnostic characters:** Large glabrous to glabrescent plant, wide corollas with compound maculations. The length of the peduncles in flower and fruit is a diagnostic character, as is the 10-costate fruiting calyx.

**Common names and uses**: None known.

**Phenology:** Flowers and fruits from November to April.

**Conservation status**: NT.

**Representative specimens examined.** MEXICO. **Jalisco**: Ayutla, headwaters of Rio Mascota [20–25 km, airline, southeast of Talpa de Allende], narrow valley of steep mountain stream ascending to the west from a point 12–13 km above (south of) El Rincón, on the road to Aserradero La Cumbre, *R. McVaugh 23467* (ENCB, MICH).

34. *Physalis melanocystis* (B. L. Rob.) Bitter, Repert. Spec. Nov. Regni Veg. 20: 369 (1924)

**Type:** Mexico, San Luis Potosi, C. G. Pringle 3285 (holotype: GH barcode 00003297!, isotypes: A 00003298!, AC not located, BR not located, COLO not located, E not located, HBG not located, MO barcode 022277, MA not located, MU not located, NDG not located, NY 172306!, OKLA not located, PH PH00029454, RSA not located, US barcode 00027310!, barcode 00027311!). *Withania melanocystis* B. L. Rob., Proc. Amer. Acad. Arts 26:171–172. 1891. (Figure 9H).

= *Physalis melanocystis* var. *melanocystis*.

= *Physalis melanocystis* var. *cernua* (Donn. Sm.) Waterf. Rhodora 69: 99 (1967).

= *Physalis porphyrophysa* Donn. Sm., Bot. Gaz. 61: 377 (1916). Type: Guatemala, Zacapa, H. Pittier 1754 (holotype: US barcode 00027356!; isotypes: F barcode V0077209F!, NY not located).

Erect shrub up to 1.5 m tall, branched, glabrous in appearance, with short, eglandular trichomes; leaves solitary, soon geminate, lanceolate to ovate-lanceolate, 4.0–7.0 cm long, 1.0–3.0 cm wide, apex acute to acuminate, base cuneate, margin entire; flowers fascicled with one to three flowers, peduncle less than 1.0 cm long; calyx with deltoid lobes 2.5–3.0 mm long; corolla campanulate stellate, greenish-yellow 0.8–1.1 cm in diameter, inconspicuous maculations; anthers yellow, 3 mm long; fruiting peduncles 1.0–1.2 cm long, thickened, fruiting calyx 10-costate 1.9–2.5 cm long, 1.5–2.0 cm wide, somewhat leathery, green or purple; mature berry orange, dark green in herbarium specimens, 1.0 cm in diameter with numerous yellow seeds ca. 1.5 mm in diameter.

**FIGURE 11:**
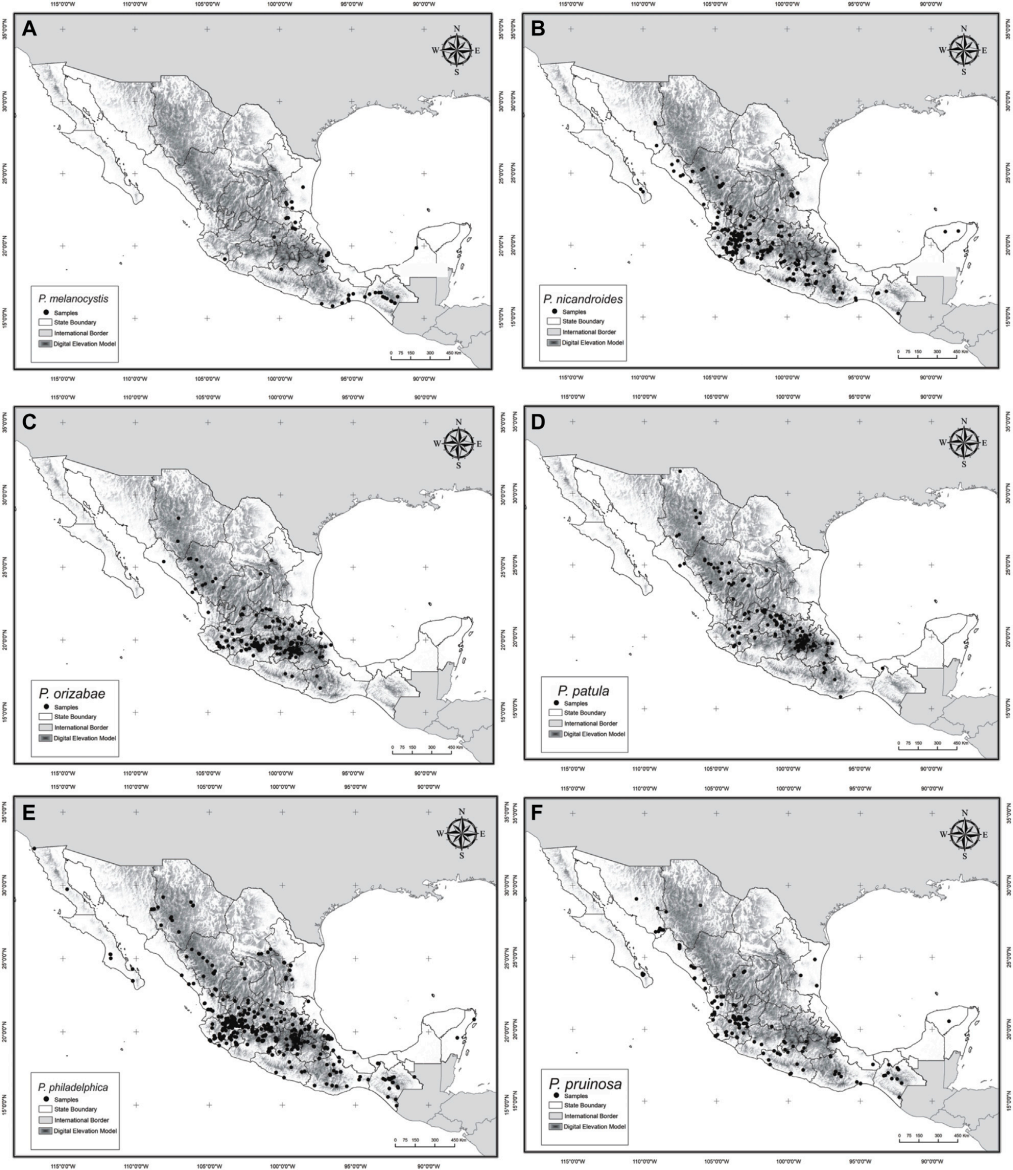
Geographic distribution of *Physalis* spp. **(A)** *P. melanocystis*, **(B)** *P. nicandroides*, **(C)** *P. orizabae*, **(D)** *P. patula*, **(E)** *P. philadelphica*, **(F)** *P. pruinosa.*

**Distribution and habitat:** In tropical deciduous, oak, and cloud forest. It is found at altitudes of 850–1,500 m. Camp., Chis., Col., Gro., Hgo., Jal., Oax., Qro., S.L.P., Tab., Tamps., Ver. (Figure 11A).

**Diagnostic characters:** A shrubby habit, narrow leaves, and aggregated, greenish-yellow flowers. The berries are an intense orange color, and the fruiting calyx is purple to almost black.

**Common names and uses**: None known.

**Phenology:** Flowering and fruiting through the year.

**Conservation status**: LC.

**Representative specimens examined.** MEXICO. **Campeche:** Calakmul, núcleo arqueológico de Calakmul, *C. Gutiérrez 529*1 (MEXU). **Chiapas:** Tzimol, a 24 km aL este de Pujiltic, sobre la carretera Venustiano Carranza-Tzimol, *E. Cabrera 3018* (MEXU). **Colima**:18 km aL SE de Colima, carretera Colima-Manzanillo, *Santana Michel 904* (IBUG). **Guerrero:** Zihuatanejo de Azueta 800 m aL SO del caserío La Vainilla, camino al mirador, *C. Gallardo 663* (MEXU) **Hidalgo:** Chapulhuacán, el Capulín 6 km, del entronque de la brecha a Pisaflores con la carretera Jacala-Tamazunchale, *P. Tenorio 2372* (MEXU). **Jalisco:** Ahualulco de Mercado, Piedras Bolas, *Corona Oceguera s.n.* (IBUG). **Oaxaca:** Salina Cruz a Santa. Clara, en la subida de Cerro Marimba, al NO de Salina Cruz, 22 km aL NO de Morro Mazatán, *R. Torres 8933* (MEXU). **Querétaro:** km 6 de la brecha de Agua Zarca a Neblinas, municipio de Landa de Matamoros, *S. Zamudio 6744* (IEB, QMEX). **San Luis Potosí:** Tamasopo, Puerto Verde, 64 km W of Cd. Valles on Hwy. to Rioverde, *P. Fryxell 3599* (MEXU). **Tabasco:** en el campamento de la escollera y 5 km de Tenosique hacia E. Zapata (ENCB). **Tamaulipas:** Gómez Farías, a 1 km de Alta Cima en el camino a Rancho del Cielo, *M. Martínez 1940* (MEXU). **Veracruz**: Dos Ríos, Rinconada, Ventura 3997 (ENCB, F).

35. *Physalis microcarpa* Urb. & Ekman, Ark. Bot. 21A (5): 59 (1927).

**Type:** Hispaniola, Santo Domingo in Llanura de Vega prope Monte-Cristi, Ekman 5841 (lectotype designated here S 04-2808!). (Figures 1N, 2E, 9I).

Erect, branched annual herb 12–40 cm tall, glabrous or pubescence with few eglandular trichomes; leaves alternate, petioles 0.8–1.0 (−2.5) cm long; blades lanceolate to ovate, 1.4–9.0 cm long, 0.4–1.8 cm wide, apex acute to acuminate, base oblique, margin entire, pubescent; flowers solitary on peduncles 2–4 mm long; calyx, lobes ca. 2 mm long, deltoid; corolla campanulate to tubular, whitish, 1.2–2.0 mm in diameter, immaculate; anthers blue, 1.4–2.1 mm long; fruiting peduncle 4–5 mm long, fruiting calyx 10-costate, 1.1–1.5 cm long, 0.7–1.0 cm wide, glabrous; mature berry ca. 7 mm in diameter, with yellowish seeds.

**Distribution and habitat:** In mangroves, riparian, and pine–oak forests, in secondary vegetation, and near corn fields. It grows at an altitude range of 750–1,400 m. Chis., Edo. Mex., Jal., Son.

**Diagnostic characters:** The species has a fragile appearance and is small; the leaves are narrow, lanceolate. The fruiting calyx is almost transparent. Corollas are tiny, white, immaculate, almost tubular, and not longer than 4 mm.

**Common names and uses:** Miltomate de culebra, tomatillo. No uses known.

**Phenology:** Flowering and fruiting in August, probably until October.

**Conservation status**: LC.

**Representative specimens examined.** MEXICO. **Chiapas:** Motozintla, outwash plain below Motozintla, *D. Breedlove 40,607* (MEXU, DS). **Jalisco**: Cuautitlán de García Barragán, Ayotitlán, Piedra Gorda, 1.5 km por la vereda que va de Las Guayabillas a El Maguey, *O. Vargas 801* (IBUG). **Sonora:** Yécora, mesa del Campanero, *T. Van Devender 96-387A* (QMEX).

36. *Physalis minimaculata* Waterf., Rhodora 69: 219 (1967)

**Type:** Mexico, Michoacán, old lava flows four miles NW of Apatzingán, R. McVaugh 17902 (holotype: MICH barcode 1109901!, isotypes: ENCB!, US barcode 00027343!)

Erect to prostrate annual herb, up to 60 cm long, branched, the entire plant pubescent, trichomes eglandular or glandular; leaves geminate, petiole 0.4–1.7 cm long; blades ovate, 1.4–2.6 cm long, 1.0–1.8 cm wide, apex acute, base oblique, margin sinuate-lobate to toothed; flowers solitary on peduncle 5–6 mm long; calyx with narrow triangular lobes ca. 2 mm long; corolla campanulate rotate, with, yellow, 0.7–1.0 cm in diameter, with conspicuous simple dark purple-brown maculations; anthers deep purple, up to 4 mm long; fruiting peduncle 5–8 mm long, fruiting calyx 10-costate 0.9–1.9 cm long, 0.5–1.5 cm wide; mature berry ca. 1.2 cm in diameter, with numerous brown seeds ca. 1.5 mm in diameter.

**Distribution and habitat:** It grows in secondary vegetation of deciduous tropical scrub, or near rivers, on rocky soils. It develops at an altitude range of 200–350 m. Herbarium specimens of this species are scarce. Mich., endemic. (Figure 10F).

**Diagnostic characters:** A small plant with long anthers of intense purple color, small maculations, and dense glandular pubescence.

**Common names and uses**: None known.

**Phenology:** Flowering and fruiting from October to February.

**Conservation status:** VU.

**Representative specimens examined.** MEXICO. **Michoacán:** Mugica, km 161 carretera Nueva Italia-La Huacana en el puente La Pastora, *M. Martínez 9457* (QMEX).

37. *Physalis minuta* Griggs, Torreya 3: 138 (1903)

**Type:** Mexico: Guerrero, Acapulco, E. Palmer 304 (lectotype designated here: MO barcode 1968849!, isolectotypes: US barcode 00027344!, GH 00077290!). (Figure 9J).

= *Physalis mimulus* Waterf., Rhodora 69:211 (1967). Type: Mexico, Colima, Socorro Island, C. H. T. Townsend s.n. (holotype: US barcode 00027342!)

Perennial herb, slightly erect to prostrate, spreading, branched, up to 60 cm long, pubescent with eglandular, short trichomes; leaves alternate, petioles 0–9–2.6 cm long; blades ovate, slightly succulent, 1.8–4.0 cm long, 0.9–2.1 cm wide, apex obtuse to acute, base oblique, margin entire; flowers solitary on peduncles 4–5 mm long; calyx with deltate lobes 1.0–1.5 mm long; corolla campanulate, yellow, translucent, 4–9 mm diameter, immaculate or with five separate reddish-brown maculations; anthers yellow, 1–2 mm long; fruiting peduncle 7–9 mm long, fruiting calyx 5-angled with a pentagonal base, 1.1–2.2 cm long, 1.1–1.8 cm wide, pubescent; mature berry ellipsoid ca. 8 mm diameter, yellow seeds 1.5–1.8 mm.

**Distribution and habitat:** It grows in coastal dunes and sandy places, protected between rocks near cliffs along the Pacific Ocean coast. It is usually scarce, but it can cover spaces of up to 3 m. It develops at an altitude range of 0–200 m. Col., Chis., Gro., Jal., Nay., Oax. (Figure 8F).

**Diagnostic characters:** A small light-yellow corolla and tiny maculations with reddish tones, 5-angled fruiting calyx, herbaceous, with thin peduncles.

**Common names and uses**: None known.

**Phenology:** Flowering and fruiting from June to April, so probably throughout the year; its presence increases after the rainy season.

**Conservation status**: LC.

**Representative specimens examined.** MEXICO. **Colima:** Manzanillo, along the road between El Ciruelo and Cuyutlán, *Gilly 11* (MICH). **Chiapas:** Tuxtla Gutiérrez, *D. Breedlove 19,991* (MO). **Guerrero:** Agua de Obispo, Mochitlán, *H. Kruse 813* (MEXU). **Jalisco**: Puerto Vallarta: a lo largo de la playa entre Villa Varadero y la desembocadura del rio Ameca, *Cházaro 6331* (IEB, WIS). **Nayarit:** Bahía de Banderas, cerro de Punta Mita, *Ramírez 4024* (IBUG). **Oaxaca:** Santo Domingo Tehuantepec, Distrito de Tehuantepec, Buenos Aires, 15 km al W de Tehuantepec, *C. Martínez 671* (MEXU).

38 *Physalis nicandroides* Schltdl., Linnaea 19: 311 (1846)

**Type:** Mexico, Hidalgo, Atotonilco el Grande, Ehrenberg 760 (lectotype designated by Martínez (1998): HAL 0042230!). (Figures 1O, 2G, 2H, 3J, 9K).

Erect annual herb up to 1 m tall, branched, glutinous, fetid, with eglandular trichomes, some glandular; leaves alternate, petioles 1.0–9.0 cm long, blades ovate, broadly ovate to cordate, 3.5–18.4 cm long, 2.5–16.0 cm wide, apex acute to slightly acuminate, base oblique, truncate to subcordate, margin entire to serrated; flowers solitary on peduncle 6–8 mm long; calyx lobes acuminate 1.5–4.5 mm long; corolla campanulate, creamy whitish, 4–6 mm in diameter, with five simple greenish maculations; anthers blue or blue-green, 2–3 mm long; fruiting peduncle 1.2–1.4 cm long, thickened, fruiting calyx 5-angled, 3.5–4.3 cm long, 2.5–2.6 cm wide, strongly cordate, caudate lobes subulate, up to 1.5 cm long, golden brown at maturity and coriaceous after drying, mature berry dark brown, up to 1.5 cm in diameter, containing numerous dark brown seeds ca. 2.5 mm in diameter.

**Distribution and habitat:** A common weed along roads or near crop fields. It develops at an altitude range of 120–2,000 m. Ags., B.C.S., Chis., Col., Dgo., Edo. Mex., Gto., Gro., Hgo., Jal., Mich., N.L., Mor., Nay., Oax., Pue., Qro., S.L.P., Sin., Son., Tamps., Ver., Yuc., Zac. (Figure 11B).

**Diagnostic characters:** The fruiting calyx is strongly 5-angled, cordate, and leathery; corollas are cream-white with pale maculations, short peduncles, and subulate caudate calyx lobes.

**Common names and uses:** Costomate, miltomate, tomatillo, matapulgas, tomate zope, tomate de culebra, tomate de perro, tomate loco, tomatillo loco, tomatón, vejiga de perro, yucu-quise. Reported as medicinal in Morelos; the species is left in the fields as an insect trap in Central Mexico. The fruits are occasionally eaten in Morelos.

**Phenology:** It flowers and bears fruit from June to April.

**Conservation status**: LC.

**Representative specimens examined.** MEXICO. **Aguascalientes:** San Francisco de los Romo, 1.8 km al ESE de la colonia Macario *J. Gómez, 823b* (MEXU). **Baja California Sur:** a 10 km de la desviación a San Antonio, cerca de Las Termópilas, *M. Martinez 9519* (QMEX). **Chiapas**: municipio de Berriozábal, flats near Berriozábal, *D. Breedlove 52,384* (CAS, MEXU). **Colima:** Comala, rancho El Jabalí, 20 km (airline), NNW of Colima in the SW foothills of the Volcan de Colima, ridgetop in hills near a shrine 4.9 km SE of Hacienda San Antonio on the road to Comala, near 19° 26.2′ N, 103° 41.8′ W, *J. Sanders 10,277* (MEXU, MICH). **Durango**: Canelas, proximidades a Canelas, terrenos de la UAF Topia, *Montemojino 2370* (CIIDIR-DGO). **Estado de México:** 15 km aL SW de Tejupilco, *E. Guizar 205* (MEXU). **Guanajuato:** Mesas del Tigre, municipio de Victoria*, E. Ventura 8560* (IEB, QMEX). **Guerrero:** municipio de Tlapa, a 8 km al N de Tlapa, *E. Martínez 2665* (MEXU, TEX). **Hidalgo:** Ajacuba, “La mesa chata,” cerro al N W del poblado Santiago Tezontlale, sierra del “Mexe,” ejido Santiago Tezontlale, *I. Díaz 1206* (MEXU). **Jalisco:** Acatlán De Juárez, Cuesta de san Marcos, above and to the east of Laguna de san Marcos, *ca.* 15 km. south-southeast of Acatlán de Juárez, *R. McVaugh 342* (ENCB, MICH). **Michoacán:** Ario de Rosales, en san José de Las Cañas, 20 km aL SW de Ario de Rosales, carretera a la Huacana, *Soto Núñez 3464* (ENCB, MEXU). **Morelos:** 3 km aL SE de La Joya, entre Cuernavaca y Yautepec, *L. Hernández 2488* (TEX). **Nayarit**: Ahuacatlán, a 4 km al S de Ahuacatlán, por el camino a Amatlán de Cañas, 21° 01′ N, 104° 30′ W, *O*. *Téllez 9947* (MEXU). **Nuevo León:** 3 km aL NE del poblado El Potosí, hacia San José de la Joya*, M. Martínez 9785* (QMEX). **Oaxaca:** Valle del Etla, *L. Smith 720* (MEXU). Puebla: municipio de Huehuetlán el Chico, ejido Tzicatlán, *Arreola TZ 570* (TEX). **Querétaro:** El Chacal, 1 km aL poniente de la Mesa de Fortín, municipio de Landa de Matamoros, *H. Rubio 156* (IEB, QMEX). **San Luis Potosí:** 64 mi NE of San Luis Potosí, *U. Waterfall 15,712* (F, NY). **Sinaloa:** municipio de Culiacán, Santa María, *González 669*4 (CAS, F, US). **Sonora:** Yécora, Rancho Los Jacales, ca. 6.2 km (by air) northwest of Santa Rosa, *T. Van Devender 2008–545* (MEXU). **Tamaulipas:** Miquihuana, *M. Martínez 9462* (QMEX). **Veracruz**: municipio de Acultzingo, 5 km ENE of center of Acultzingo, *M. Nee 33,132* (NY). **Yucatán:** Izamal, *Gaumer 1446* (F, US). **Zacatecas:** cerro La Cantarilla, 8 km al S de Moyohaua, *E. Enriquez 1412* (MEXU).

39. *Physalis orizabae* Dunal, Prodr. [A. P. de Candolle] 13 (1): 452 (1852)

**Type:** Mexico, Veracruz, Mount Orizaba, C. J. W. Schiede 607 (holotype: HAL 0042231!). (Figure 9L).

= *Physalis subintegra* Fernald, Proc. Amer. Acad. Arts 35: 567 (1900). Type: Mexico, Edo. de Mexico, Sierra de Las Cruces, C. G. Pringle 8225 (holotype: GH 00077373!, isotypes: BR not located, F V0073011F!, OKLA not located, UC 104036, US barcode 00027366!, barcode 01014183! VT not located).

Erect or spreading perennial herb, the rhizome about 30 cm long, 2.0 cm thick, branching from the base, pubescence highly variable in length and density, the trichomes eglandular, septate, white; alternate or geminate leaves, petiole 0.8–4.0 cm long; blades ovate to lanceolate 1.7–10.0 cm long, 1.2–5.6 cm wide, attenuated toward the acute or obtuse apex, base truncate, oblique, shortly decurrent, margin entire or with two to three teeth per side; flowers solitary on peduncles 0.8–2.0 cm long; calyx with ovate-to-attenuate lobes 4–7 mm long, corolla yellow, 1.5–2.6 cm in diameter, rotate campanulate, with simple-to-compound, reddish-brown to purple maculations; anthers 2–4 mm long, purple or blue; fruiting peduncle 0.8–2.1 mm long, fruiting calyx 10-costate with five more conspicuous angles or nearly round, 2.3–3.7 cm long, 1.5–3.0 cm wide; mature berry 1.0–1.2 cm in diameter with numerous yellow seeds 2–2.5 mm in diameter.

**Distribution and habitat:** On slopes and margins of disturbed oak forests and adjacent clearings of pine–oak forests, secondary grasslands, and subtropical scrub. From 1,850 to 2,700 m. Ags., Cd. Mex., Chih., Dgo., Edo. Mex., Gto., Gro., Hgo., Jal., Mich., Mor., Nay., N.L., Oax., Pue., Qro., S.L.P., Ver., Zac. (Figure 11C).

**Diagnostic characters:** The plant has entire leaves, large corollas with strongly contrasting maculations and purple or blue anthers, a large fruiting calyx, and peduncles. The species can be confused with *P. chenopodiifolia*; however, the latter is easily distinguished by its cinereous appearance and different leaf form.

**Common names and uses:** Vonsummate, tomate cimarrón, tomate de mata, tomatillo, xapindikua. The plant is used medicinally, and the fruit is eaten.

**Phenology:** It flowers and bears fruit from June to September.

**Conservation status**: LC.

**Representative specimens examined.** MEXICO. **Aguascalientes**: Calvillo: Sierra del Laurel, near the Jalisco–Aguascalientes border, *ca.* 10 miles southeast of Calvillo (3 h by horse from rancho de Los Adobes), *McVaugh 18,439* (ENCB). **Ciudad de México:** Tlalpan, Área Natural Protegida: Volcán Pelado, *J. Rivera 3783* (MEXU). **Chihuahua:** Guachochi, alrededores de Guachochi, *R. Hernández 8625* (MEXU). **Durango:** Pueblo Nuevo, 35 km al W de El Salto, *Hernández 7768* (MEXU). **Estado de México:** National Park Lagunas de Zempoala, *R. McAdams 52* (MEXU). **Guanajuato:** más o menos 6 km aL SW de San franco, municipio de San Diego de la Unión, *E. Carranza 5060* IEB, QMEX). **Guerrero:** 15 km W-SW de Chichihualco, *M. Mayfield 998* (MEXU). **Hidalgo:** roadside near Real de Monte above Pachuca, *A. Sharp 4457 (*MEXU). **Jalisco**: Acatic, 6 km al suroeste de Acatic, *Reynoso Dueñas 84* (IBUG). **Michoacán:** Cerro La Alberca, cerca de Villa Jiménez, municipio de Villa Jiménez, *J. Rzedowski 40208, 40192* (IEB). **Morelos**: km 47 carretera de cuota México-Cuernavaca, límites entre DF y Morelos, A. Rodríguez 2782 (MEXU). **Nayarit:** Acaponeta, La Ciénega on ridge approximately 10 miles northwest of mesa del Nayar*, Norris 14,575* (MICH). **Nuevo León**: General Zaragoza, Sierra El Soldado, camino a Puerto Pinos J. Villarreal 4989 (MEXU). **Oaxaca**: Santiago Textitlán, distrito: Sola de Vega, Paraje Tierra de Yunta, A. Sánchez 1692 (MEXU). **Puebla:** 5 km al E de Xalitzintla*, P. Tenorio 15,931* (MEXU). **Querétaro:** 4–5 km al S de El Parador Santa Martha, municipio de Landa de Matamoros, *E. Carranza 1799* (IEB). **San Luis Potosí:** 21 mi al E de San Luis Potosí, *L. Dorr 1984* (MEXU). **Veracruz**: El Limón, Ramos 233 (MEXU). **Zacatecas**: Valparaiso, road to Huejuquilla el Alto, Jal., one mile west of the road junction 18 miles south of Valparaiso on the road to Mezquitic, Jal, 22° 38′ N, 103° 48′ W, *R. McVaugh 17763* (MICH).

40. *Physalis parvianthera* Waterf., Rhodora 69: 204 (1967)

**Type:** Morelos: Sierra de Chalchi, cerca de Tepoztlán, Paray 1614 (holotype: ENCB!).

Erect herb, 25–40 cm tall; stems villous, with articulated more or less viscid trichomes; petioles 10–14 mm long; leaves deltoid-ovate, ovate or lanceolate, more-or-less villous, 25–30 mm long, 20–30 mm wide; apex obtuse to acute, base cuneate to truncate, decurrent, margin entire; flowers solitary on peduncles 3–5 mm long; calyx lobes lanceolate 1–1.5 mm long; corolla campanulate, yellow, immaculate, 15–20 mm in diameter; anthers violet 1.5–1.8 mm long; fruiting peduncles 6–8 mm long, fruiting calyx 10-costate, 8–9 mm long, 5–7 mm wide.

**Distribution and habitat:** Probably in pine–oak forest, such as the forest in Tepoztlán, at 1,700 m. Mor. (Figure 10B).

**Diagnostic characters:** The small, nearly 10-costate fruiting-calyx, immaculate yellow corollas, small violet anthers, and soft vestiture. The deltoid-ovate leaves are uncommon in *Physalis.*

**Common names and uses**: None known.

**Phenology:** Flowering in August.

**Conservation status**: DD.

**Representative specimens examined. MEXICO. Morelos:** Tepoztlán, Sierra de Chalchi, cerca de Tepoztlán, *Paray 1614* (ENCB).

41. *Physalis patula* Mill., Gard. Dict., ed. 8. n. 12 (1768)

**FIGURE 12:**
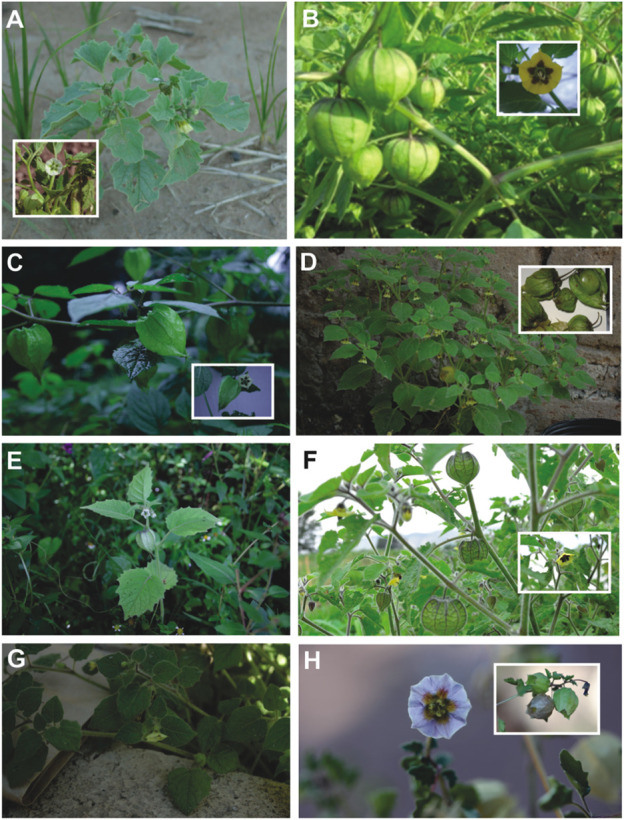
Details of flowers and fruits of *Physalis* spp. **(A)** *P. patula*, **(B)** *P. philadelphica*, **(C)** *P. porrecta*, **(D)** *P. pringlei*, **(E)** *P. pruinosa*, **(F)** *P. pubescens*, **(G)** *P. queretaroensis*, **(H)** *P. purpurea.*

**Type:** Mexico, Veracruz, 1731, Houston s.n. (holotype: BM 000775431!). (Figures 1P, 12A).

= *Physalis foetens* Poir. Encycl. (J. Lamarck and al.) Suppl. 2. 348 (1811). Type: Mexico, A. Bonpland s.n.; 1803–1804 but reportedly from “Peru” without further locality (FI not located).

= *Physalis foetens* var. *neomexicana* (Rydb.) Waterf., Rhodora 60 (714): 168 (1958). Type: United States, New Mexico, A. Fendler 678 (lectotype, designated by Waterfall 1958 GH 00003288!).

= *Physalis subulata* Rydb., Bull. Torrey Bot. Club 22 (7): 306 (1895). Type: Mexico, Chihuahua, waste grounds Guerrero, C. G. Pringle 1344 (holotype: GH 00003294!, isotypes: F barcode V00073013F!, MEXU not located, NY barcode 138890!, OKL not located).

Erect annual herb up to 50 cm tall; stem branched, glutinous, fetid, densely pubescent, with eglandular and glandular trichomes; leaves alternate, petioles 1.0–3.5 cm long; blades ovate, 3.5–7.0 cm long, 2.0–4.6 cm wide, apex acute, base oblique to cuneate decurrent, margin sinuate or serrated; flowers solitary on peduncles 3–7 mm long; calyx with subulate lobes 1.5–3.5 mm long, corolla campanulate, pale yellow, 1.0–2.5 cm in diameter, with five simple yellowish-brown to olive-green diffuse maculations; anthers yellow or blue when dry, 1.5–4.0 mm long; fruiting peduncle 0.4–1.2 cm long, fruiting calyx 5-angled, 1.5–3.0 cm wide, 1.8–2.2 cm long; mature berry brown, 1.0–1.2 cm in diameter with numerous dark brown seeds ca. 2 mm in diameter.

**Distribution and habitat:** Dry or semi-arid areas, in thorny or subtropical thickets, grasslands or hillsides near pine or oak forests, or as weeds in plots within the forest. It grows at an altitude range of 900–2,700 m. Ags., Chih., Col., Cd. Mex., Dgo., Edo. Mex., Gto., Hgo., Jal., Mich., Oax., Pue., Qro., Sin., Tlax., Ver., Zac. (Figure 11D).

**Diagnostic characters:** A fetid annual plant with dense and glutinous pubescence, toothed leaves, 5-angled fruiting calyx, and wide corollas with clear or diffuse maculations.

**Common names and uses:** Tomate, tomate de burro, tomatillo, hierba del zopilote, jaltomate hediondo. No uses known.

**Phenology:** It flowers and bears fruit from July to November and bears fruit until March.

**Conservation status**: LC.

**Representative specimens examined.** MEXICO. **Aguascalientes:** Aguascalientes, highway to Ojuelos, Jal., nine miles east of Aguascalientes, *R. McVaugh 16,672* (MICH). **Chihuahua**: en el camino a Cumbres de Majalca, municipio de Chihuahua, *M. Martínez 9655* (QMEX). **Colima:** Comala, rancho El Jabalí, 20 km (airline), N of Colima in the SW foothills of the Volcan de Colima, El Agostadero, ridgetop in hills above the shrine 4.9 km SE of Hacienda San Antonio on the road to Comala, near 19° 26.2′ N, 103 \° 41.8′ W, *J. Sanders 11,806* (MEXU, MICH). **Ciudad de México:** Tláhuac, vertiente E del cerro de Santa Catarina, *J. Rzedowski 26,037* (CAS, MEXU, NY). **Durango:** Nombre de Dios, al SW de La Parrilla, *S. González 165* (CIIDIR-DGO). **Estado de México:** Huixquilucan, 2 km al W de Río Hondo, *J. Rzedowski 29,569* (MEXU). **Guanajuato**: 5 km al S de San Felipe, sobre la carretera a Dolores Hidalgo, municipio de San Felipe, *J. Rzedowski 47,319* (IEB, QMEX). **Hidalgo:** Ajacuba Jagüey “El Palo Seco,” planicie de Tulancalco, aprox. 4 km sobre el camino de terracería que se inicia al S de Emiliano Zapata, ejido Tecomatlán, *I. Díaz 1200* (MEXU). **Jalisco:** Ojuelos, potrero La Colorada, 5 km al W de la carretera Ojuelos-Lagos de Moreno, *M. Alcocer 62* (MEXU). **Michoacán:** Zamora, halfway between Jacona and Zamora, along Mexico highway 15, 19° 55′ N, 102° 18′ W, *Iltis 648* (ENCB, WIS). **Oaxaca:** La Loma Pachona, 6 km al NE de Gpe. Cuautepec o 1 km al E del entronque carr. Huajuapan de León-Tehuacán y la brecha de Cuautepec, A. Salinas 3680 (MEXU). **Puebla:** Xochitecatl 2300 cerca de Texmelucan, *W. Boege 154* (MEXU). **Querétaro:** Santa Rosa, casa de Juan Antonio Isla, municipio de Querétaro, *E. Argüelles 1410* (ENCB, MEXU). **Sinaloa:** Culiacán a 20 km al N de Culiacán, carretera Culiacán a Guamúchil, ladera NE del cerro La Chiva, a 1 km aL O de Mirasoles, *R. Vega 6263* (UAS). **Tlaxcala:** Tzompantepec, San Juan Quetzalcuapan, *H. Vibrans 184* (MEXU). **Veracruz:** Puerto del Aire, carretera Orizaba-Tehuacán, *Rosas 808* (MEXU). **Zacatecas:** Jalpa, pastured hills five miles southwest of Jalpa, *R. McVaugh 18508A* (ENCB, MICH).

42. *Physalis pennellii* Waterf., Rhodora 69: 116 (1967)

**Type:** Mexico San Luis Potosí rocky limestone west of Santa Ana, above Potrero, Sierra de Catorce F. W. Pennell 17523 (holotype: US barcode 00027351!, isotypes: NY barcode 138880!, PH not located).

Perennial herb, 5–20 cm tall; articulated trichomes, long and short to 1.5 mm long, partly glandular; petioles 5–11 mm long, blades ovate or broad-ovate 13–32 mm long, 12–25 mm wide, apex obtuse to acute, base cordate, margin undulate or entire; solitary flowers on peduncles 3–6 mm long; calyx with deltoid or ovate-deltoid lobes densely pubescent; corolla campanulate, yellow, 7–13 mm long, 10–14 mm wide, maculations dark brown; anthers violet, ca. 2.5 mm long; fruiting peduncles 9–15 mm long; fruiting calyx 10-costate, 15–20 m long, 11–13 mm wide; mature berry 8–12 mm wide with numerous yellow seeds ca. 1.5 mm in diameter.

**Distribution and habitat:** rocky limestones, S.L.P. (Figure 8C).

**Diagnostic characters:** *Physalis pennellii* is most similar to *P. sordida* Fern. but is a smaller plant, with a mixture of long trichomes on the leaves and a more densely long-hairy flowering calyx.

**Common names and uses**: None known.

**Phenology:** Flowering in July.

**Conservation status**: NT.

**Representative specimens examined.** MEXICO. **San Luis Potosí:** 3 km pasando Alamillos, al S de Real de Catorce, *M. Martínez 9560* (QMEX).

43. *Physalis philadelphica* Lam., Encycl. Méth. Bot. 2: 101, 1786. Lam. herb. P, IDC 6207. 471: I.5)

**Type:** Cultivé en 1784 au Jardin du Roi; nous le croyons originaire de l’Amérique septentrionale (lectotype designated by Waterfall Rhodora 69 (778): 213. 1967 P barcode P00357705!). (Figure 12B).

= *Physalis chenopodifolia* Willd., sp. Pl., ed. 4 [Willdenow] 1https://www.ipni.org/n/77093275-12): 1023 (1798).

= *Physalis philadelphica* var. *immaculata* Waterf., Rhodora 69: 215 (1967). Type: Mexico, Chihuahua, silty flat in desert 14 miles NE of Parral, U.T. Waterfall 16,115 (holotype: OKLA not located, isotypes: GH not located, United States not located).

= *Physalis philadelphica* subsp. *ixocarpa* (Brot. ex Hornem.) Sobr.-Vesp. and Sanz-Elorza, Acta Bot. Malac. 32: 233 (2007).

*= Physalis philadelphica* var. *minor* Dunal, Prodr. [A. P. de Candolle] 13 (1): 450 (1852). Type: Mexico, Tamaulipas, Tampico, J. L. Berlandier 20 (lectotype designated by Pretz and Deanna 2020 G barcode G00343158, isolectotype: BM barcode BM000775484).

= *Physalis philadelphica* var. *parviflora* Waterf., Rhodora 69: 215 (1967). Type: Mexico, Nayarit, Cerro Sanganguey 12 miles SE of Tepic, Feddema 582 (holotype: MICH barcode 1109899!, isotype: OKLA not located).

= *Physalis philadelphica* f. *pilosa* Waterf., Rhodora 69 (no. 778): 214 (1967). Type: Guatemala, Finca La Alameda near Chimaltenango, P. C. Stanley 79899 (holotype: F barcode V0077208F!).

= *Physalis laevigata* M. Martens and Galeotti in Bull. Acad. Brux. xii. I. 131 (1845). Type: Mexico, presa de Morelia de Michoacán, H. G. Galeotti 1188 (lectotype designated by Pretz and Deanna 2020 BR barcode 000005529483, isolectotypes: BR barcode 000005528332, P barcode P00409133).

= *Physalis cavaleriei* H.Lév., Repert. Spec. Nov. Regni Veg. 11: 295 (1912). Type: China, Guizhou, Kouy-Tehéou, Kouy-Yang sous les rochers, J. Cavalerie 3800 (lectotype designated by Pretz and Deanna (2020) E barcode E00284483, isolectotypes: E barcode 00284484, G barcode G00343185, K barcode K000759437, P barcode P000061015).

Erect annual herb, 3–100 cm high, stem with adpressed trichomes 0.5 mm long; leaves alternate, petioles 1–2.5 cm long, ovate to lanceolate 2–9 cm long, 1–5 cm wide, apex acute, base attenuate, sometimes oblique up to 1.5 cm, margin dentate with 8–10 teeth per side, and almost glabrous; flowers solitary on peduncles up to 5 mm long; calyx with triangular lobes ca. 2 mm long, marginally pubescent; corolla rotate, yellow, 0.8–2.5 cm in diameter with five purple diffuse maculations, corolla throat densely pubescent; anthers blue, 2–5 mm long, convolute after anthesis; fruiting peduncles 7–10 mm long, fruiting calyx 10-costate, 1–3 cm long, 1.8–2.0 cm wide; mature berry spherical, 1–5 cm in diameter, green, yellow, or purple-tinged at maturity, with numerous yellow seeds ca. 2 mm in diameter. In large-fruited specimens, the berry tears the fruiting calyx.

**Distribution and habitat:** A common weed of cities, roads, and agricultural fields; it grows up to 2,500 m asl. Present in all the Mexican states except Camp., Tlax., and Yuc. (Figure 11E).

**Diagnostic characters:** Due to its weedy tendency and economic importance, *Physalis philadelphica* is the most widely distributed species in the country. It is also cultivated in some regions of Mexico for its edible acidic fruit. The species is characterized by the large globose calyx in the fruit, corollas with simple maculations, convolute anthers, and the margin of the leaves with short teeth.

**Common names and uses:** Tomatillo, tomate, tomate verde, tomate de hoja, tomatillo de cáscara, tomate cuarentano, tomate de bolsa, yashal tumat, quelite, miltomate, tomate fresadilla, fresada. The fruit is commonly used in the preparation of sauces, either raw, fried, or boiled.

**Phenology:** Flowers and fruits mainly after the rainy season; however, specimens with flowers were examined in March.

**Conservation status**: LC.

**Representative specimens examined.** MEXICO. **Aguascalientes:** San José de Gracia, 200 m adelante de El Milagro, en la desviación a Santiago, *O. Vargas 779* (CUCBA, MEXU). **Baja California:** San Quintín, productora de plantas “Baja Plants,” *M. Martínez s/n* (QMEX). **Baja California Sur:** Todos Santos antes del pueblo cerca del río, *M. Martínez 9523* (QMEX). **Chiapas:** arroyo de San Nicolás, está al Este de San Cristóbal de las Casas, *A. Méndez 84*73 (MEXU). **Chihuahua:** Cuauhtemoc, Napavechia Valley, *K. Adams 30* (ARIZ). **Ciudad de México:** Tláhuac, Mixquic, *V. Popper 105* (MEXU). **Coahuila:** pasando Ramos Arizpe, ca. 3 km al E hacia Monterrey, *M. Martínez 9793* (QMEX). **Colima:** Manzanillo**,** cerro de La Cruz, *Campos 7* (IBUG). **Durango**: Santiago Papasquiaro, Escuela Jose Ramon Valdez, *R. Worthington 11,315* (UTEP). **Estado de México:** Naucalpan de Juárez, Barranca cercana a El Huizachal, *J. Rzedowski 25109* (MEXU). **Guanajuato:** 2 km al S de San José de Tránsito, municipio de Silao, cerca del aeropuerto, *J. Rzedowski 49,832* (IEB). **Guerrero:** La Unión de Isidoro Montes de Oca, Vallecitos, *G. Hinton 11,44*9 (MEXU). **Hidalgo:** Mineral de El Chico, calles aledañas al poblado, *A. Rodríguez 2773* (CUCBA, MEXU). **Jalisco**: Acatic, brecha crucero viejo de Acatic, *Reynoso 66* (IBUG). **Michoacán:** Crucero a Curimeo, carretera Zacapu-Purándiro, municipio Panindícuaro, *E. Pérez 1839* (IEB). **Morelos:** Curva La Pera, carretera de cuota México-Cuernavaca, *A. Rodríguez 2786* (CUCBA, MEXU). **Nayarit**: Santa Teresa, camino a La Mesa del Nayar, a 1 km aL SW, 22° 30′ N, 104° 48′ W, *Flores 1381* (MEXU). **Nuevo León:** Iturbide, Loma La Bandera, NW of Ejido Santa Rosa, 4.1 mi S of Iturbide, *J. Sullivan 2578* (TEX)**. Oaxaca:** poblado de Santa María Ixcatlán (UTM 1974844, 14Q 691,794) E. Rivera 36 (MEXU). **Puebla:** San Andrés Cholula, a 1.2 km aL sur de San Antonio Cacalotepec, *Y. Guarneros 348* (MEXU). **Querétaro:** Municipio de Landa de Matamoros, Misión de Tilaco, a 2.5 km de Landa de Matamoros, *M. Martínez 2788* (QMEX). **Quintana Roo**: 1 km NW of Puerto Morelos, Campo Agropecuario del Centro de Investigaciones de Quintana Roo, *G. Davidse 20,028* (MEXU). **San Luis Potosí:** Rioverde, between Rio Verde and Plazuelo (km 89.9) MEX 69 km 93 *Robert Bye 18944* (MEXU). **Sinaloa:** Concordia, Santuario El Palmito (La Chara Pinta), La Liebre, *J. Rubio 107* (CIIDIR). **Sonora:** Sirebampo vicinity. 9.5 km south on Mexico 15 from Las Bocas Road turnoff, 3.5 km west on Sirebampo Rd., 11.5 km south (by air) San Jose de Masiaca, *S. Friedman 094–9*5 (ASU). **Tabasco:** Huimanguillo, hacia la Laguna de los Limones y 5 km de la desviación a Fco. Rueda, *M. Magaña 73*8 (MEXU). **Tamaulipas:** Miquihuana, en el vado que atraviesa la carretera, *M. Martínez 9469* (QMEX). **Veracruz:** Chacaltianguis, Benito Juárez, *M. Nee 29,270* (XAL). **Zacatecas:** Jalpa camino a la colonia Morelos, 3 km a la salida a Apozol *E. Enríquez 634 (*MEXU).

44. *Physalis porrecta* Waterf., Rhodora 69: 237 (1967)

**Type:** Costa Rica, Prov. San Jose, vicinity of El General, A. F. Skutch 2931 (holotype: US barcode 00027357!, isotypes: GH barcode 00077361!, NY barcode 138883!) (Figures 3L, 12C).

Erect perennial herb, 0.4–1.3 m tall; stem branched with puberulent trichomes; petioles (2–) 3–7 cm long; blades ovate, 5–11 cm long, 4–7 cm wide, apex acute, base decurrent, margin unevenly dentate or entire; flowers solitary on peduncles 4–5 mm long; calyx with narrowly tapered lobes 1.5–4.0 mm long, corolla rotate campanulate, yellow, 12–15 mm in diameter, with diffuse not strongly contrasting maculations; anthers yellow or violet, 1.8–2.3 mm long; fruiting peduncles 6–15 mm long, fruiting calyx 5-angled, 3–5 cm long, 2.2–3.0 cm wide, glabrous; mature berry 12–15 mm in diameter with numerous yellow seeds ca. 1 mm in diameter.

**Distribution and habitat:** Mountain cloud forest at 1,200–1,500 m asl. Chis., Gro., Oax. (Figure 10C).

**Diagnostic characters:** According to Waterfall (1967), the species is characterized by its usually abruptly beaked fruiting calyx, yellowish corollas varying from immaculate to yellowish maculations, and diffuse margin. The stem, branches, and petioles present a variable density of pubescence.

**Common names and uses:** Moench’o, miltomate, tomatillo. No uses known.

**Phenology:** Flowering and fruiting from July to February.

**Conservation status**: DD.

**Representative specimens examined.** MEXICO. **Chiapas:** Unión Juárez, En el camino Talquian-Cima del Volcan Tacana, mitad del camino hacia La línea, frontera con Guatemala Pilar Zamora-Tavares et al. 273 (IBUG). **Guerrero:** Chilpancingo de los Bravo, Omiltemi, La Frutilla, camino a 3 cruces *A. Méndez 302* (MEXU). **Oaxaca:** vicinity of Cafetal Concordia, 1 April 1933, *Morton and Makrinius 2556* (US).

45. *Physalis pringlei* Greenm., Proc. Amer. Acad. Arts 35: 311 (1900)

**Type:** Mexico, Oaxaca, Sierra de Clavellinas, C.G. Pringle 6001 (lectotype selected by Waterfall (1967) GH 00077362!, isolectotypes: MEXU barcodes 27677!, 28217!, 27675!, BM not located, BR not located, NY 138884!, UC 104028, US barcode 00027358!, VT not located). (Figure 12D).

= *Physalis pringlei* var. *curtiloba* Waterf., Rhodora 69 (778): 228 (1967). Type: Mexico, Durango, San Ramón, E. Palmer 114 (holotype: US barcode 00027359!).

Erect perennial herb 1.0–1.7 m tall; stem densely covered with glutinous jointed spreading trichomes of varying lengths; leaves geminate throughout, petioles 1–3 cm long; blades ovate, 4–8 cm long, apex acute to acuminate, base attenuate, margin with one to three irregular teeth or small lobes, or entire, sparsely appressed-hairy, often with more abundant trichomes along the veins; flowers solitary on peduncles 0.9–1.5 cm long; calyx lobes ovate to lanceolate 7–10 mm long; corolla rotate campanulate, yellow, 18–22 mm wide with five dark brown maculations, corolla throat pubescent; anthers bluish, 2.2–3.0 mm long; fruiting peduncles 1.5–2.0 cm long, fruiting calyx 5-angled 2.5–4.0 cm long, 1.0–1.5 cm wide; mature berry 1.0–1.4 cm in diameter, dark yellow seeds 1.8–2 mm.

**Distribution and habitat:** In moist *Abies* and pine–oak forests. Alt. 2,750–3,100 m. Cd. Mex., Dgo., Oax. (Figure 10F).

**Diagnostic characters:** A rotate-pentangular corolla with dark maculations, fruiting calyx 5-angulated, dense pubescence with some glandular trichomes.

**Common names and uses**: None known.

**Phenology:** Flowering and fruiting in September and October.

**Conservation status**: LC.

**Representative specimens examined.** MEXICO. **Cd. Mex**: Sierra del Ajusco *C. G. Pringle 6216* (MEXU). **Durango:** San Ramón, *E. Palmer 114* (IBUG). **Oaxaca:** Sierra de Clavellinas *C. G. Pringle 6001* (IBUG).

46. *Physalis pruinosa* L., sp. Pl. 1: 184 (1753)

**Type:** Habitat in America (holotype: LINN 247.13!). (Figures 1Q, 12E).

= *P. maxima* Mill., Gard. Dict., ed. 8. n. 15 (1768). Type: Mexico, Veracruz without further locality, Houston s.n. (holotype: BM 000775441!, Sloane collection, photoholotype BH!, OKLA!).

= *Physalis cordifolia* Dunal, Prodr. [A. P. de Candolle] 13 (1): 441 (1852). Type: from a cultivated plant in the Lugduno-Baravo Gardens, De la Roche s.n. (holotype: G-DC SIB 160733/1!)

Erect annual herb 1.0–1.5 m tall; stem branched, glutinous, fetid, pubescent with a mixture of eglandular short and long trichomes, some glandular; petiole 0.8–4.7 cm long; blades broadly ovate to cordate, 3.6–13.0 cm long, 2.1–6.0 cm wide, apex acuminate, base oblique, truncate to subcordate, margin usually serrate; solitary flowers on peduncles 1.2–4.3 cm long; calyx with subulate caudate lobes up to 8 mm long; corolla campanulate, spreading to reflected, yellow, 0.8–1.3 cm in diameter, maculations diffuse, light pinkish-brown; anthers yellow, 2.5–3.0 mm long; fruiting peduncle 2.8–5.5 cm long, fruiting calyx 5-angled, 2.3–6.0 cm long, 1.1–4.0 cm wide, initially green, becoming golden brown; mature berry brown, 1.5 cm in diameter with numerous brown seeds ca. 2 mm in diameter.

**Distribution and habitat:** A ruderal weed or in ravines, margins of oak or tropical deciduous forest or their ecotones at 120–1,850 m. Chis., Chih., Edo. Mex., Col., Gro., Jal., Mor., Nay., Oax., Qro., Sin., Son., Tamps., Ver., Yuc., Zac. (Figure 11F).

**Diagnostic characters:** A long flower peduncle, pinkish-brown maculations, yellow anthers. The species can be confused with *P. nicandroides* because both are pubescent, glutinous, and very similar in plant habit and appearance; both have a strongly 5-angled fruiting calyx. However, *P. pruinosa* has a mixture of short and long trichomes in the stem and a thin fruiting peduncle.

**Common names and uses:** Miltomate, vejiga de perro, farolito, chipin sox, tomate de culebra, tomate de hoja, tomate de bolsa, tomate, tomatillo, tomate grande, papay-zeal, tumat’cho, tumal, tumat. The fruit is eaten.

**Phenology:** Flowering and fruiting from June to November.

**Conservation status**: LC.

**Representative specimens examined.** MEXICO. **Chiapas:** Comitán de Domínguez, 1 km al SE del enronque Tzimol-Uninajab, camino a Uninajab, *A. Reyes 1098* (MEXU). **Chihuahua:** La Cienegita, Rio Mayo, *H. Gentry 2632* (F, MEXU). **Colima:** Comala, cerro Caleras, 4 km antes de Tecolapa, autopista Colima-Guadalajara, *O. Vargas 814* (IBUG). **Estado de México:** Luvianos, Temascaltepec, *G. Hinton 4592* (MEXU). **Guerrero:** Coyuca de Benítez, Santa Bárbara, *G. Hinton* 8503 (MEXU). **Jalisco:** La Huerta: arroyo Colorado, cerca de Los Pozos, *E. Lott 528* (MEXU). **Michoacán:** Apatzingán, barranca of rio Cancita at the bridge nine miles southeast of Apatzingán, *R. McVaugh 17,981* (ENCB, MICH). **Morelos:** 2.5 km al N y 4 km al W de Huautla, *R. Ayala 198* (MEXU). **Nayarit:** Bahía de Banderas, seaward-facing mountainsides 1–1.5 miles above La Cucaracha, 12–13 miles south of Las Varas, *R*. *McVaugh 19,226* (ENCB, MICH). **Oaxaca:** 5 km al NW de Totolapan, *M. Martínez 1904* (MEXU). **Querétaro:** hacia Casa de Máquinas, Cadereyta, *A. Herrera 200* (QMEX). **Sinaloa:** Escuinapa, 18 km al E de Escuinapa. Brecha Escuinapa-Corral de Piedra, *P. Tenorio 2913* (MEXU). **Sonora:** Rancho Cuadritos, ca. 7 km al E de Mesa del Seri hacia Sahuaripa *M. Martínez 9838* (QMEX)**. Tamaulipas:** 19 km al E de la carr Zaragoza-Gonzalez, arroyo de Minas hacia ejido El Cabrito, *C. González 133* (MEXU, UAT). **Veracruz:** Totutla, Encinal, *F. Ventura 7046* (ENCB, MEXU) **Yucatán:** sin más localidad, *G. Gaumer 24,310* (F). **Zacatecas**: Jalpa, pastured hills five miles southwest of Jalpa, *McVaugh 18508A* (ENCB, MICH).

47. *Physalis pubescens* L., Sp. Pl. [Linnaeus] 1: 183 (1753)

**Type:** “In India utraque,” without further locality, date, or collector (lectotype selected by Waterfall 1958: LINN 247.11!). (Figures 3K, 12F).

Synonyms in Martínez (1998) and Pretz and Deanna (2020), except for *P. latiphysa* Waterf., which we consider a distinct species.

*= Physalis angustiloba* Waterf., Rhodora 69: 320 (1967). Type: Mexico, Jalisco [Talpa de. Allende], hills two miles north of La Cuesta, road to Talpa de Allende, R. McVaugh 21141 (holotype: MICH 1109911!, isotypes: OKLA not located, LL!, US barcode 00027312!)

Erect annual to biennial herb 10–150 cm tall; pubescent, and pilose to glandular-pilose with multicellular trichomes up to 3 mm long; petioles 1.5–7.0 cm long, blades ovate to deltoid, 2–12 cm long, 1–7 cm wide, apex acute to acuminate, base obtuse to truncate, sometimes oblique up to 3 mm, margin irregularly dentate to almost entire, villous especially on the lower surface; flowers solitary on peduncles 2–7 mm long; calyx villose with triangular lobes 1.5–3.0 mm long, corolla rotate, yellow, 0.9–2 cm in diameter, with five strongly contrasting obtuse maculations, corolla throat densely pubescent; anthers purple or blue, seldom yellow, ca. 2 mm long; fruiting peduncle 5–10 mm long, fruiting calyx strongly 5-angled, 1.2–3.0 cm long, 1.8–2.5 cm wide, always longer than wide, and villous throughout with multicellular trichomes; mature berry green tinged with purple or yellow, ca. 1 cm in diameter with many brown seeds ca. 1.5 mm in diameter.

**FIGURE 13:**
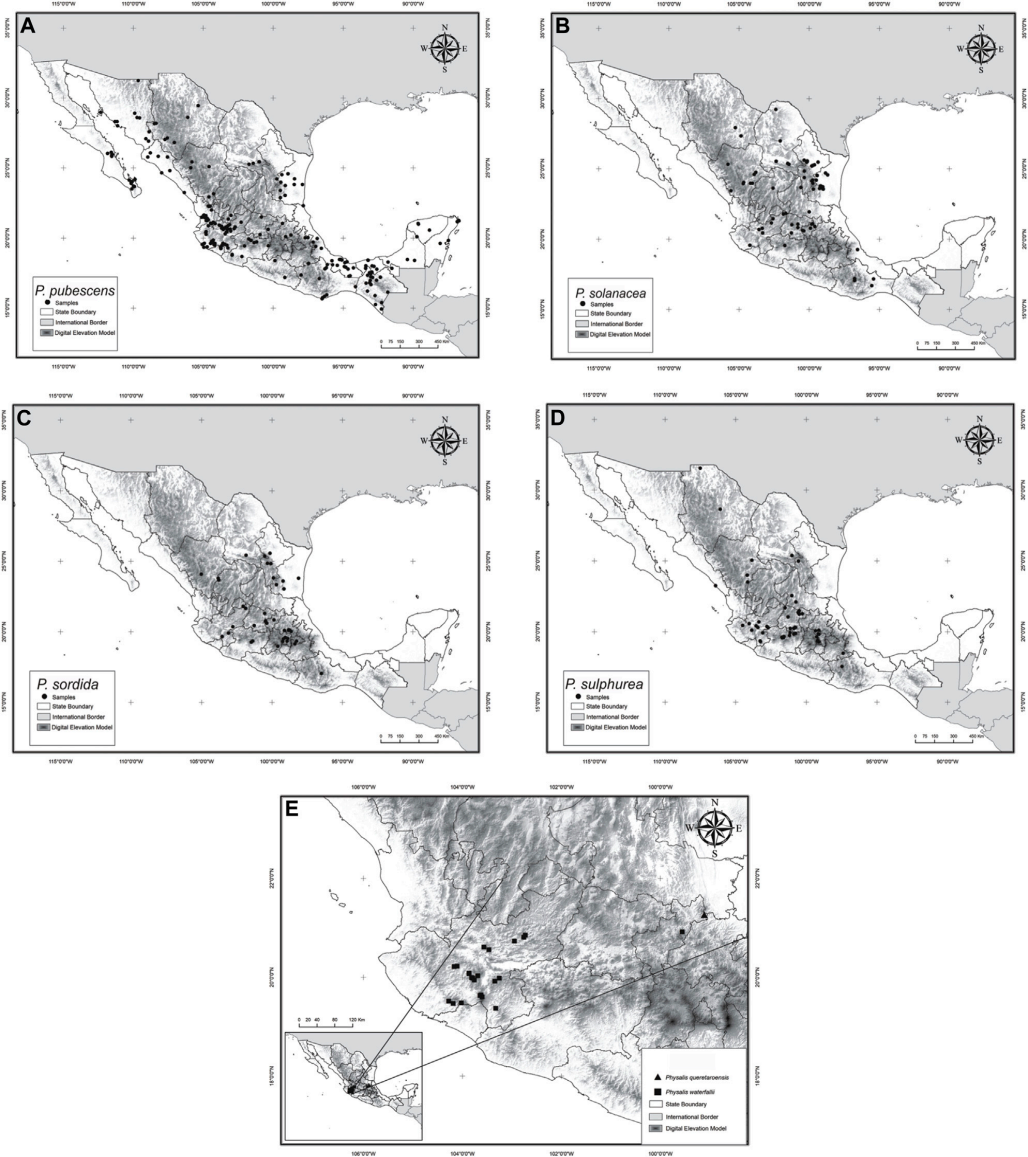
Geographic distribution of *Physalis spp*. **(A)** *P. pubescens*, **(B)** *P. solanacea*, **(C)** *P. sordida*, **(D)** *P. sulphurea*, **(E)** *P. queretaroensis* and *P. waterfallii*.

**Distribution and habitat:** The plant grows on disturbed areas of streams, grasslands, and pine–oak forest, also associated with crop fields, from sea level up to 2,600 m asl. B.C.S., Camp., Chis., Chih., Coah., Col., Dgo., Gro., Hgo., Jal., Mich., Nay., NL., Oax., Pue., Qro., Q.R., Sin., Son., Tab., Tamps., Ver., Yuc. (Figure 13A).

**Diagnostic characters:** *Physalis pubescens* can be recognized by the pubescent stem, blue anthers, simple corolla maculations, and five-angled fruit calyx.

**Common names and uses:** Cocostomat, tomatillo, miltomate, tomate de culebra, chichol antivo, tumat’cho. The fruit of weedy plants is used in the preparation of sauces or eaten raw as fresh fruit in several areas (Martínez, 1998).

**Phenology:** It flowers and bears fruit mainly from June to October, but there are some specimens collected in February that bear fruit.

**Conservation status**: LC.

**Representative specimens examined.** MEXICO. **Baja California Sur:** Isla Cerralvo, en el arroyo Las Delicias, *M. Martínez 9500* (QMEX). **Campeche:** Carmen, 2 km antes de llegar al límite del estado de Campeche con Tabasco, *C. Chan 6137* (MEXU). **Chiapas:** Angel Albino Corzo steep, heavily wooded slope near the Rancho Viejo of the Finca Prusia, *A. Méndez 3607* (MEXU). **Chihuahua:** Guachochi, Cabórachi, 20 km al E. de Guachochi, *R. Hernández 8562* (MEXU). **Coahuila:** Tanque Nuevo, *M. Martínez 9606* (QMEX). **Colima:** Comala, rancho el Jabalí, 22 km (airline), N of Colima in the SW foothills of the Volcan de Colima, Colima/Jalisco line passes through the ranch, near 103° 1.8′ W, 19° 26′ N, El Agostadero, ca. 5 km S of San Antonio, *J. Sanders 11,330* (MEXU, MICH). **Durango**: Mezquital, 1 km aL NE de santa María de Ocotán, *S. González 1455* (CIIDIR-DGO). **Guerrero:** Temixco, second barranca E of Temixco, *Y Mexia 8760* (CAS, F, NY, US). **Hidalgo:** Eloxochitlán, 6.5 km al E, *O. Alcántara 2172* (MEXU). **Jalisco:** Autlán de Navarro, south- and west-facing slopes, precipitous rocky mountain sides 11–12 miles southwest of Autlán (approximately 2 miles below the pass), *McVaugh 857* (ENCB). **Michoacán:** Aguililla, an area locally referred to as Cerritos de Agua, *ca.* three miles below lumber camp at Dos Aguas, nearly west of Aguililla, Lat. approx. 18° 45′ N, long. 102° 56′ W, *McVaugh 17,865* (ENCB, MICH). **Nayarit:** Ixtlán, km 7–10 terracería a Cacalotán, que empieza a 500 m al S del límite de los estados Nayarit-Jalisco, 21° 07′ 00″ N, 104° 17′ 00″ W, *Téllez 11,066* (MEXU). **Nuevo León:** Horsetail Falls, 30 mi SE of Monterrey, *Thompson 294* (TEX). **Oaxaca:** Santa María Jacatepec en el poblado La Joya del ejido Corriente Ancha, *J. Calzada 15,426* (MEXU). **Puebla:** Zapotitlán de Méndez, *G. Villalobos 23* (MEXU). **Quintana Roo:** Felipe Carrillo Puerto, 100 m al E del Ramonal, *R. Durán 756* (MEXU). **Querétaro:** 1–2 km aL SW de Barricales, municipio de Jalpan, *E. Carranza 3335* (IEB, QMEX). **Michoacán:** Cerro La Alberca, cerca de Villa Jiménez, municipio de Villa Jiménez, *J. Rzedowski 40,204* (IEB). **Sinaloa:** Culiacán, poblado de Jesús María y cercanías de la presa Adolfo López Mateos (El Verjonal), *R. Vega 3305* (QMEX). **Sonora:** La Huerta, approximately 2 km inland from Ensenada La Manga, *R. Felger 85-346B* (MEXU). **Tabasco:** Paraíso dentro del rancho La Noria a 1 km de Paraíso hacia la Ría Moctezuma, *M. Magaña 1007* (MEXU). Tamaulipas: Rio Corona, 2 km al E de la Hacienda Santa Engracia, *M. Martínez 1945* (MEXU, UAT). **Veracruz**: Hidalgotitlán, Rio Soloxuchil, campamento *Hnos. Cedillo, Vázquez et al. 145 (MEXU, XAL).* **Yucatán:** Yaxcabá, Tixcacaltuyub, *V. Rico 709* (MEXU).

48. *Physalis purpurea* Wiggins Contr. Dudley Herb. 3: 74, tab. 19 (1940)

**Type:** Mexico, Sonora low hills and flats near the tannery east of Guaymas. Ira L. Wiggins 6352 (holotype: CAS 0004035!). (Figures 1R, 12H).

Erect perennial herb, branched, 25–75 cm tall, spreading-puberulent, especially on the stems, petioles, and peduncles; petioles 2–5 cm long, blades ovate to ovate-deltoid, 2.5–5.0 cm long, 2–5 cm wide, apex acuminate, base truncate to cordate, margins coarsely and irregularly dentate to sinuate dentate; flowers solitary on peduncles 17–25 mm long; calyx with broadly lanceolate to deltoid lobes 1–2 mm long; corolla violet or purplish, 9–13 mm wide; anthers yellow, 1.5–3.0 mm long; fruiting peduncles 2–4 cm long, fruiting calyx 10-costate, 23–30 mm long, 17–24 mm wide; mature berry green or yellow, nearly spherical, 10–18 mm wide with numerous yellow seeds ca. 2 mm in diameter.

**Distribution and habitat:** Endemic to the humid canyons around Guaymas, near creeks or waterfalls, from sea level to 160 m elevation. Son. (Figure 10C).

**Diagnostic characters:** A rare perennial plant with purple flowers and dentate leaves; the fruiting calyx is open at the apex.

**Common names and uses**: None known.

**Phenology:** Flowering and fruiting from October to March.

**Conservation status**: DD.

**Representative specimens examined.** MEXICO. **Sonora:** Cerro Algodones, 5 km al W de San Carlos, *M. Martínez 9844* (QMEX).

49. *Physalis queretaroensis* M. Martínez and L. Hern., Acta Bot. Mex. 46: 73 (1999)

**Type:** Mexico, Querétaro, 2–5 km al S del Parador Santa Martha, municipio de Landa de Matamoros, bosque de encino en ladera de cerro, E. Carranza 2585 (holotype: IEB!). (Figure 12G).

Decumbent perennial herb 70 cm long, sparsely hairy, with a woody base; stems creeping, rooting at the nodes, internodes very long, 6.5–10 cm long; leaves geminate throughout, petioles 1.5–2.3 cm long; blades ovate to lanceolate, 3.0–5.5 cm long, 1.5–3.0 cm wide, apex acuminate, base truncate to cordate, margin entire or with 2–3 teeth per side, with a few eglandular trichomes on the upper and lower sides; solitary flowers on peduncles 1.5–2.5 cm long; calyx with acuminate lobes 0.5 mm long; corolla rotate, yellow, 1–2 cm in diameter, with five purple maculations, slightly pubescent corolla tube; anthers blue or yellow with blue lines 3 mm long; fruiting peduncles 1.3–2.0 cm long, fruiting calyx 5-angled, 1.1–1.9 cm long, 0.8–1.0 cm wide with a few trichomes, berry 0.5 cm in diameter with numerous yellow seeds 1.5 mm in diameter.

**Distribution and habitat:** Endemic to Qro.; it grows in pine, oak, and cloud forests at 1,700–1,850 m asl. (Figure 13F).

**Diagnostic characters:** A perennial trailing herb that roots at the nodes, with few eglandular trichomes.

**Common names and uses**: None known.

**Phenology:** Flowering and fruiting from July to December.

**Conservation status**: LC.

**Representative specimens examined.** MEXICO. **Querétaro:** El Parador, 2 km al NW de El Madroño, Landa de Matamoros, *E. González 969* (IEB).

50 *Physalis rydbergii* Fernald, Proc. Amer. Acad. Arts 35: 569 (1900)

**Type:** Mexico, Sinaloa, Imala, E. Palmer 1713 (holotype: GH 00077369!, isotypes: GH 00077370!, NY barcode 138888!, US barcode 00027363!).

Erect perennial herb, 20–60 cm tall; stem branched, glandular-pubescent with trichomes 2–3 mm long; basal leaves deciduous, the upper ones geminate, petioles 1.0–3.5 cm long; blades ovate-lanceolate to lanceolate or trulate, 2–7 cm long, 1.5–4.0 cm wide, apex acute, base rounded to attenuate, margin toothed with 2–5 teeth per side, glandular-pubescent on both surfaces, but especially on margin and veins; flowers solitary on peduncles 2–3 mm long; calyx with deltoid lobes ca. 3 mm long, corolla rotate, yellow, ca. 1 cm in diameter with five strongly contrasting purple maculations; anthers blue, ca. 3 mm long; fruiting calyx 10-costate, slightly invaginated, 1.5–2.0 cm long, 1.0–1.5 cm wide, glandular-pubescent; berry ca. 1 cm in diameter with numerous yellow seeds ca. 2 mm diameter.

**Distribution and habitat:** It grows in xerophytic scrub and low deciduous forest, 80–2,100 m. Gto., Mich, Qro., Sin. (Figure 5E).

**Diagnostic characters:** The species is distinguished by its glandular trichomes, perennial habit, and small flowers and fruits. Our description differs from that of Waterfall (1967) in the color of the anthers, which he describes as yellow, and the blade size, but he was only familiar with the material of the type.

**Common names and uses**: None known.

**Phenology:** Flowering and fruiting from October to November.

**Conservation status**: DD.

**Representative specimens examined.** MEXICO. **Guanajuato:** 4 km al E de San Diego de la Unión, sobre el camino a La Jaula, municipio de San Diego, *J. Rzedowski 52,099* (IEB, QMEX). **Michoacán:** cerca de San José Itzícuaro, municipio de Morelia, *J. Rzedowski 45,382* (IEB, QMEX). **Querétaro:** fondo del Sótano del Barro, Santa María de Cocos, municipio de arroyo Seco, *G. Cifuentes 69* (QMEX). **Sinaloa:** Imala, *E. Palmer 1713* (GH).

51. *Physalis sancti-josephi* Dunal Prodr. [A. P. de Candolle] 13 (1): 451 (1852)

**Type:** Mexico, Hidalgo, prope San José del Oro, reg. frig. C. J. W. Schiede (holotype: HAL 42233!).

Erect perennial herb 60–100 cm tall; stem with few branches, pubescent with glandular multicellular trichomes, glabrescent; basal leaves alternate, upper leaves geminate, petioles 2.5–4 cm long, blades ovate, 4–8 cm long, 2.5–4.5 cm wide, apex acuminate, base rounded, margin entire or with one to three teeth per side, pubescent with multicellular glandular trichomes on both surfaces; flowers solitary on peduncles 1.0–1.4 cm long; calyx with triangular lobes 4–5 mm long; corolla rotate, yellow, 1.2–2.0 cm in diameter with strongly contrasting solid or compound blue maculations, corolla throat densely pubescent; anthers yellow or yellow with blue lines, ca. 3 mm long; fruiting peduncle 1.0–1.2 cm long, fruiting calyx 10-costate, slightly invaginated, 2.5–3.0 cm long, 1.5–2.5 cm wide, glandular-pubescent; fruit 0.7–1.1 cm in diameter with numerous yellow seeds ca. 1.5 mm in diameter.

**Distribution and habitat:** It develops in pine, oak, and cloud forests at 1,600–1,800 m. Hgo., Jal, Nay., Qro., S.L.P. (Figure 13E).

**Diagnostic characters:** The species is poorly characterized. It resembles underdeveloped specimens of *P*. *coztomatl*, with which it shares habitat and distribution. They differ in that *P. coztomatl* has a stem densely covered by trichomes up to 3 mm, whereas *P. sancti-josephi* has much smaller trichomes and corolla, and the maculations can be solid.

**Common names and uses**: None known.

**Phenology:** It blooms all year round, but it has been collected with flowers and fruits from February to March and with fruit only in May.

**Conservation status**: DD.

**Representative specimens examined.** MEXICO. **Hidalgo:** prope San José del Oro, reg. frig. C. J. *W. Schiede* (HAL). **Jalisco**: Autlán de Navarro, arroyo de Las Juntas, 18–19 km aL SE de Autlán, 3–4 km al N de El Zarzamoro, 19° 37′ 16″ N, 104° 16′ 36″ W, *Guzmán 984* (ZEA). **Nayarit:** Tepic, 10 km al W de El Izote, brecha al Cuarenteño, 21° 30′ N, 104° 58′ W, *P. Tenorio 15,684* (ENCB, MEXU, MICH)**. Querétaro:** 11 km de el Cañón, camino a la Parada, municipio de Jalpan, *E. Carranza 1425* (IEB, QMEX). **San Luis Potosi:** Alvarez *E. Palmer 220* (F, GH, MICH, NY, UC, US)

52. *Physalis solanacea* (Schltdl.) Axelius, Phytologia 79: 11 (1995)

**FIGURE 14:**
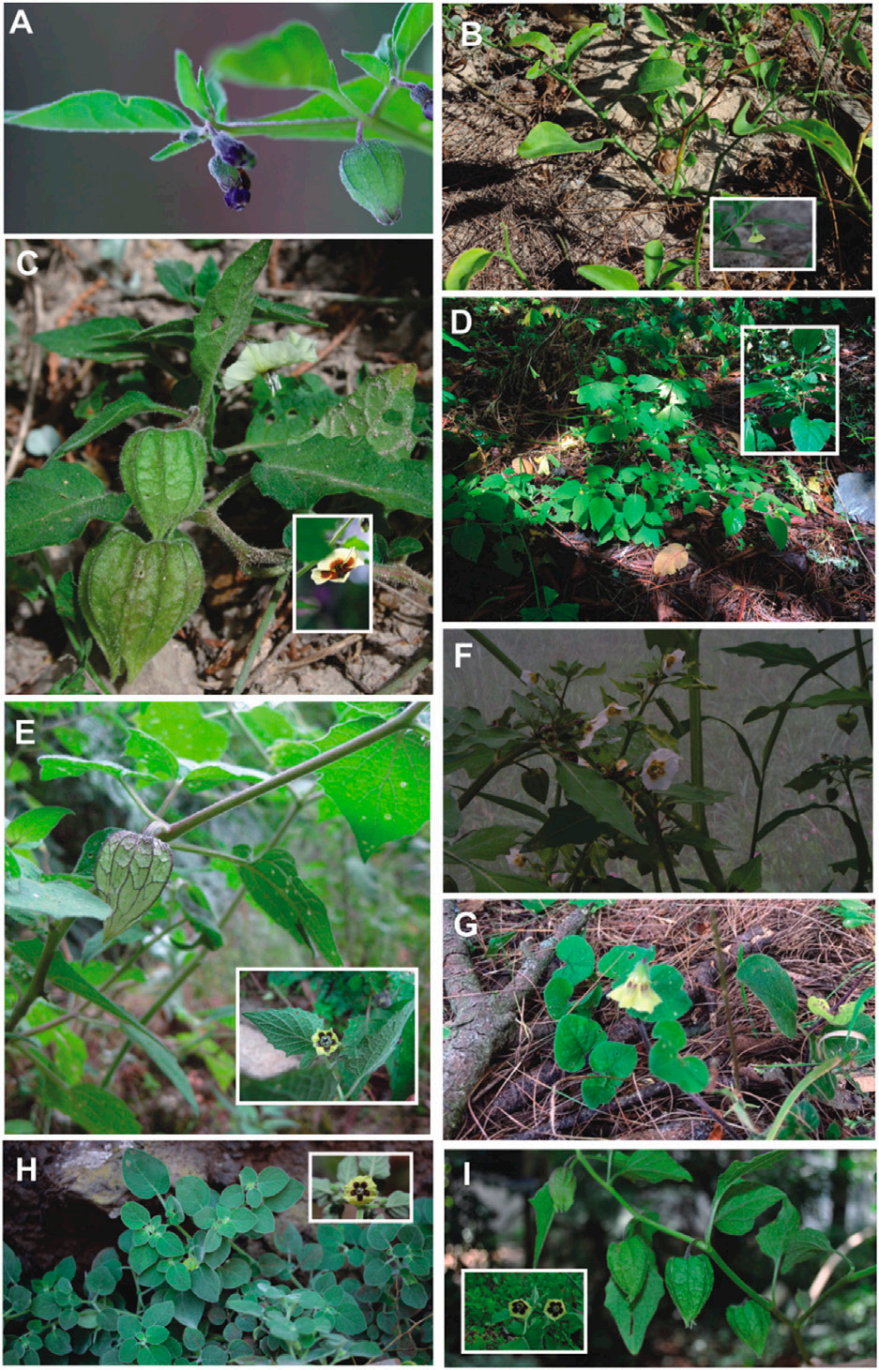
Details of flowers and fruits of *Physalis* spp. **(A)** *P. solanacea*, **(B)** *P. spathulifolia*, **(C)** *P. sordida*, **(D)** *P. subrepens*, **(E)** *P. tamayoi*, **(F)** *P. sulphurea*, **(G)** *P. volubilis*, **(H)** *P. vestita*, **(I)** *P. waterfallii.*

**Type:** Cult. in Horto Botánico Halensis 1838, “e seminis in Mexico locis calidioribus coll. C. Ehrenberg” (holotype: HAL 0033693!). *Margaranthus solanaceus* Schltdl., Index Seminum Hort. Hal. 1838: 8 (1838). (Figures 1S, 14A).

Erect annual plant 6–100 cm tall; stem with few branches, glabrous at the base and with a few eglandular multicellular trichomes on the upper part; petioles 0.5–2.5 cm long, the upper leaves almost sessile; blades ovate to lanceolate, 1.5–7.0 cm long, 0.5–3.0 cm wide, acute apex, base acute, sometimes oblique up to 5 mm, margin entire, sometimes slightly wavy or with three to four teeth per side, glabrous or with a few eglandular trichomes on the margin and veins; flowers solitary, peduncles absent or up to 4 mm long; calyx ca. 2 mm long with triangular, pubescent lobes, corolla urceolate, purple to greenish-yellow, 2–4 mm long; anthers blue, ca. 1 mm long; fruiting peduncle ca. 4 mm long, fruiting calyx 10-costate, 0.6–1.5 cm long, 0.6–1.1 cm wide, pubescent with eglandular trichomes; mature berry purple, 4–6 mm in diameter with few (4–10) yellow seeds ca. 2 mm in diameter.

**Distribution and habitat:** The species grows in desert scrubs, tropical deciduous forest, grasslands, roadsides, streams, and farmland. It blooms from August to December at 630–2,400 m. Chih., Coah., Dgo., Gto., Hgo., Jal., Mich., N.L., Oax., Pue., Qro., S.L.P., Tamps., Ver., Zac. (Figure 13B).

**Diagnostic characters:** A purple, urceolate corolla, small fruits, 10-costate fruiting calyx. It is distinguished from *P. lagascae* and *P. ampla*, both of which bear small fruits, by its glabrous calyx.

**Common names and uses:** Tomatillo, mata pulgas. No uses known.

**Phenology:** Flowering and fruiting from July to December.

**Conservation status**: LC.

**Representative specimens examined.** MEXICO. **Chihuahua:** km 98 de la carretera Camargo-Delicias, municipio de Saucillo, *M. Martínez 9680* (QMEX). Coahuila: 5 km antes de General Cepeda, *M. Martínez 9594* (QMEX). **Durango:** Mezquital, 3 km de Temohaya por el camino a Mezquital, *González* 1601 (MEXU). **Guanajuato:** 15 km al W de Salvatierra sobre la carretera a Yuriria, *J. Rzedowski 38793* (IEB, MEXU). **Hidalgo:** Cardonal, Tolantongo, *J. Rzedowski 113347* (IEB). **Jalisco**: Tala, a lo largo del arroyo Caliente y Los Letreros, Bosque Escuela La Primavera, *A. Rodríguez 1436* (IBUG). **Michoacán:** estación del tren, municipio de Charo, *J. Escobedo 2125* (IEB). **Nuevo León:** Montemorelos, en la salida hacia General terán, ca. 3 km del centro del pueblo, *M. Martínez 9808* (QMEX). **Oaxaca:** Heroica Ciudad de Huajuapan de León, a 3 km del límite Puebla-Oaxaca, a un costado de la carretera 125 Tehuacán-Huajuapan de León. Distrito Huajuapan, *L. Pérez 4* (MEXU). **Puebla:** Caltepec, La Laguna, Cerro El Gavilán, E de Caltepec, *P. Tenorio 21082* (MEXU). **Querétaro:** Misión de Tilaco, municipio de Landa, *M. Martínez 2789* (QMEX). **San Luis Potosí:** Guadalcázar, Los Aguajitos, 11 km aL NE de Guadalcázar, hacia Pozo de Acuña, *R. Torres 17,151* (MEXU). **Tamaulipas:** carretera Victoria-Llera, en el km 203 en la torre de telecomunicaciones, municipio de Llera, *M. Martínez 9894* (QMEX). **Veracruz:** Pánuco, 4 km al S del límite del estado de Veracruz y Tamaulipas, 10 km aL SW de la carretera Tampico-Pánuco, *L. Nevling 37*1 (MEXU). **Zacatecas:** 15 miles NE of Estación Camacho, in wash one mile NW of Pico de Teyra, in Arroyo de Borrego, S of Cerro de Borrego *J. Henrickson 13515* (TEX).

53. *Physalis sordida* Fernald, Proc. Amer. Acad. Arts 35: 568 (1900)

**Type:** Mexico, Oaxaca, Boca de León, Telixtlahuaca, A. L. Smith 637 (holotype: GH 00077372!). (Figure 14C).

Erect or prostrate perennial herb 20–60 cm tall; stem highly branched, densely covered with glandular trichomes; basal leaves alternate, the upper ones geminate, petioles 0.7–2.0 cm long; blades ovate to trulate, 1.5–4.5 cm long, 1–2.5 cm wide, apex acute, base attenuate to rounded, margin toothed with four–six teeth per side, sometimes almost entire, densely glandular-pubescent on both surfaces; flowers solitary on peduncles 5–7 mm long, glandular-pubescent; calyx with triangular lobes 3–4 mm long, glandular-pubescent; corolla rotate, yellow, 1.5–2.0 (2.5) cm in diameter with strongly contrasting blue maculations, sometimes glandular-pubescent outside; anthers blue or yellow with blue lines, ca. 3 mm long; fruiting peduncles 6–7 mm long, fruiting calyx 10-costate, slightly invaginated, 1.5–2.5 cm long, 1–2 cm wide, glandular-pubescent; mature berry purple ca. 1.5 cm in diameter, glutinous with numerous yellow seeds ca. 2 mm diameter.

**Distribution and habitat:** It grows on roadsides or secondary vegetation of pine, oak, and xerophytic scrub, 1,800–2,000 m. Coah., Dgo., Edo. Mex., Gto., Hgo., Jal., Mich., NL., Oax., Pue., Qro., S.L.P., Tam., Zac. (Figure 13C).

**Diagnostic characters:** A dense glandular pubescent plant with blue anthers and a slightly leathery 10-costate fruiting calyx. The plant is commonly covered with dirt.

**Common names and uses**: None known.

**Phenology:** Flowering and fruiting from March to November.

**Conservation status**: LC.

**Representative specimens examined.** MEXICO. **Coahuila:** camino entre Sierra el Coahuilón y Sierra La Marta*, M. Yañez 196* (MEXU). **Durango:** Along Hwy 40, 4 km NE of Yerbaniz, 21 km SW of Cuencamé, *G. Diggs* (XAL). **Estado de México:** Texcoco, carr. Mex.-D.F. km 25, *L. Scheinvar 526* (MEXU). **Guanajuato:** San Luis de la Paz *R. Hernández 134a* (MEXU). **Hidalgo:** Ajacuba La Mesa Chata, cerro al NW del poblado Santiago Tezontlale, sierra del Mexe, ejido Santiago Tezontlale *I.Díaz 1221* (MEXU). **Jalisco:** Ojuelos, Paso de La Troje, near km 36, southwest of Ojuelos on the road to Aguascalientes, rocky slopes on and near Cerro La Campana, *McVaugh 16836* (MICH). **Michoacan:** A 13 km aL SE de Villa Madero, Carretera a Nocupetaro (IBUG). **Nuevo León:** road from San Rafael to 18 de Marzo and Galeana, ca. eight miles E of San Rafael, 4.4 miles E of La Boca *L. J. Dorr* (TEX)**. Oaxaca:** Teotitlán a 6 km SE, on highway between Tehuacán and Telixtlahuaca, *S. Douglas* (MEXU)**. Puebla:** 3 km al N de Cuicatlán, *O. Téllez* (QMEX). **Querétaro:** Cerro El Azteca (Los Cajones) L. Hernández 4855 (MEXU). **San Luis Potosí:** Chiefly in the region of San Luis Potosí, *C. C. Parry* (US). **Tamaulipas:** Sierra de Guatemala. Barbara’s Patch. Halfway down Mine road toward Mine lookout *J. Sullivan s. n.* (ENCB). **Zacatecas:** S slope of La Bufa *R. Dressler* (MO).

54. *Physalis spathulifolia* (Torr.) B. L. Turner, Phytologia 93 (2): 264 (2011)

**Type:** United States, Texas, Rio Bravo, sea beach, A.C.V. Schott 30. (holotype: NY 402103!, isotype: F barcode V0072990F!). *Physalis lanceolata* var. *spathulifolia* Torr. in Emory, Rep. U.S. Mex. Bound., Bot. [Emory] 153 (1858). (Figure 14B).

= *Physalis cinerascens* var. *spathulifolia* (Torr.) J.R. Sullivan, Syst. Bot. 10 (4): 444 (1985).

= *Physalis viscosa* var. *spathulifolia* (Torr.) A. Gray Proc. Amer. Acad. Arts 10: 67 (1874).

Decumbent perennial herb, 30–40 cm tall, with a long horizontal rhizome covered with branched trichomes throughout, mixed with glandular trichomes in young parts; leaves geminate, petioles 5–13 mm long or absent; blades spathulate, rarely ovate, 5.5–8.0 cm long, 2.5–4.5 cm wide, apex obtuse, base attenuate, margin entire; flowers solitary on peduncles 2.2–3.5 cm long; calyx with triangular lobes ca. 2 mm long; corolla rotate-campanulate, yellow, 1.3–1.5 cm in diameter with five solid purple strongly contrasting maculations; anthers yellow, 3 mm long; fruiting peduncle 3.0–3.5 cm long, fruiting calyx 10-costate, not invaginated at the base, 3.5–4.0 cm long, 2.5–3.0 cm wide, mature berry yellow, 2 cm in diameter, containing numerous yellow seeds ca. 2 mm in diameter.

**Distribution and habitat:** Restricted to the sandy shores of Tamaulipas, at sea level. Tamps. (Figure 5B).

**Diagnostic characters:** A perennial with branching trichomes restricted to sandy shores.

**Common names and uses**: None known.

**Phenology:** Flowering and fruiting from September to March.

**Conservation status**: LC.

**Representative specimens examined.** MEXICO. **Tamaulipas:** Playa de La Pesca, municipio de Soto la Marina, *M. Martínez 94*82 (QMEX).

55. *Physalis subrepens* Waterf., Rhodora 69: 229 (1967)

**Type:** Mexico, Hidalgo, Trinidad Iron Works, C. G. Pringle 13,591 (holotype: VT not located; isotypes: F V0073012F!, GH 00077374!, MICH 1109896!, OKLA not located, TEX!, UC not located, US barcode 00027367!). (Figure 14D).

Erect perennial herb up to 1.5 m tall, with a rhizome 20 cm long, pubescent with eglandular trichomes; leaves alternate to geminate almost from the base, petioles 1.3–2.5 cm long; blades ovate to broadly ovate or suborbicular, 2.6–7.0 cm long, 2.2–4.3 cm wide, apex acute or slightly attenuate, base oblique, cuneate, somewhat decurrent to subcordate, margin entire or with few teeth, hairy with scattered trichomes on veins and margins or glabrous; flowers solitary on peduncle 0.8–2.3 cm long; calyx with ovate to triangular lobes 3–4 mm long, corolla rotate campanulate, pale yellow, 1.3–1.6 cm in diameter, with simple brownish maculations; anthers blue, 2–3 mm long; fruiting peduncle 1.3–2.5 cm long, fruiting calyx 5-angled, 1.6–3.3 cm long, 1.2–1.8 cm wide; mature berry 0.6–1.0 cm in diameter.

**Distribution and habitat:** It develops in pine–oak or cloud forests, on slopes, or in open places. It grows at altitudes close to 2,100 m. Edo. Mex., Hgo., Jal., Ver.

**Diagnostic characters:** The species is hairy, with roots at the basal nodes, geminate leaves, long peduncles in flower and fruit, and a 5-angled fruiting calyx. *Physalis subrepens* can be confused with *P. gracilis* because of the geminate leaves, prostrate habit, and long flower and fruit peduncles, but *P. gracilis* has a 10-costate fruiting calyx.

**Common names and uses:** Tomatillo; no uses known**.**

**Phenology:** It bears flowers and fruit from April to August, but its phenology is probably longer.

**Conservation status:** LC.

**Representative specimens examined.** MEXICO. **Estado de México:** Contreras *Galvan s.n.* (MEXU). **Hidalgo**: Barranca near Honey Station, *Pringle 13,440* (GH, MICH, US, VT). **Jalisco:** Autlán de Navarro, ladera S en la falda del cuamil Zermeño, Las Joyas, *Vázquez García 3266* (MEXU, ZEA). **Veracruz**: Huayacocotla, 1 km SW of Ixtatetla, *Nee* and *Taylor 26845* (XAL).

56. *Physalis sulphurea* (Fern.) Waterf., Rhodora 69: 224 (1967)

**Type:** Mexico, Distrito Federal, the valley of Mexico, C. G. Pringle 8215 (lectotype selected by Waterfall (1967): GH 77375!) *Margaranthus sulphureus* Fernald Proc. Amer. Acad. Arts 35:566–567. 1900. (Figures 1T, 14F).

Erect or creeping annual herb 8–70 cm tall; stems with few branches, angled, hollow, glabrous at the base, and puberulent with eglandular trichomes on younger parts; basal leaves alternate, the upper ones geminate, petioles 0.5–4.0 cm long; blades ovate to lanceolate, 1.5–5.5 cm long, 0.8–3.0 cm wide, apex acute, base attenuate, decurrent, sometimes oblique up to 1 cm, margin entire to toothed, glabrous to sparsely puberulent with multicellular trichomes on the margin and veins; flowers solitary on peduncles 0.8–2.0 cm long; calyx with triangular lobes ca. 1 mm long; corolla rotate, whitish or pale yellow, 1.0–1.5 cm in diameter with slightly contrasting brown maculations, corolla throat glabrous; anthers blue, ca. 2 mm long; fruiting peduncles 0.8–1.5 cm long, fruiting calyx 10-costate, 0.8–1.4 cm long, 0.5–1.2 cm wide; mature berry green ca. 0.5 cm in diameter with numerous yellow seeds ca. 3 mm in diameter.

**Distribution and habitat:** In wet places around lakes, dams, and stream shores, at 1,700–2,000 m, Cd. Mex., Dgo., Gto., Jal., Mich., Qro. (Figure 13D).

**Diagnostic characters:** The whitish or pale-yellow corolla and hollowed stem; it is the only aquatic species of *Physalis*.

**Common names and uses:** Jitomate, tomatillo. No uses known.

**Phenology:** Blooms from April to December.

**Conservation status:** LC.

**Representative specimens examined.** MEXICO. **Ciudad de Mexico:** Xochimilco, Cienega Grande, *E. Martínez 39,817* (MEXU). **Durango:** Villa Unión, al E del 18 de Agosto, *González 1234* (CHAPA, CIIDIR-DGO, ENCB). **Guanajuato:** Salvatierra, 5 km aL NE de Salvatierra, sobre la carretera a Celaya, *Rzedowski 38572* (ENCB, MEXU). **Jalisco:** Chapala, University of Guadalajara Country Club, Chapala, on Laguna de Chapala, 21° 17′ N, 103° 4′ W, *Iltis 29,162* (IBUG, WIS). **Michoacán:** alrededores de Copándaro, municipio de Villa Jiménez, *J. Rzedowski 46491* (IEB). **Querétaro**: presa de Santa Catarina, *A. Herrera 198* (QMEX).

57. *Physalis tamayoi* O. Vargas, M. Martínez & Dávila, Brittonia 53 (4): 507–509 (2001)

**Type:** Mexico, Jalisco: Tapalpa, dirt road from Tapalpa to Chiquistlán, 2002′02´´N, 10,350′23´´W, O. Vargas 876 (holotype: IBUG!; isotypes: MEXU!, NY!). (Figure 1U).

Erect shrub up to 1.5 m tall, stems with eglandular trichomes mixed with glandular trichomes except in young parts; leaves alternate at the base, soon geminate, petioles 1.0–3.8 cm long; blades ovate, 3.4–9.1 cm long, 2.3–6.7 cm wide, apex acuminate, base oblique to truncate, margin serrate; flowers solitary on peduncles 0.5–1.0 cm long; calyx with acuminate lobes, 1.5–2.5 mm long, corolla campanulate-rotate, yellow, 1.0–1.8 cm in diameter, with dark brown simple-to-compound maculations; anthers purple or blue, 2.5–3.0 mm long; fruiting peduncle 0.9–1.4 cm long, densely pubescent, fruiting calyx 10-costate, 2.4–3.5 cm long, 1.6–2.5 cm wide, velutinous, reticulate with purple veins; mature berry ca. 1.1 cm in diameter, yellow seeds, 1.5–1.8 mm in diameter.

**Distribution and habitat:** Marginal vegetation of pine–oak forest, under tejocote trees (*Crataegus mexicana*), or in ravines within the forest, at an altitude range of 1,910–2,150 m. Endemic, Jal. (Figure 10E).

**Diagnostic characters:** A bush or suffrutex with glandular pubescence, small flowering calyx, and 10-ribbed fruiting calyx. It shares with *P. lignescens* the small flowering calyxes and reflex lobes.

**Common names and uses:** None known.

**Phenology:** Flowering and fruiting from July to November.

**Conservation status:** NT.

**Representative specimens examined.** MEXICO. **Jalisco**: Tapalpa, dirt road from Tapalpa to Chiquistlán, *P. Zamora 207* (IBUG).

58. *Physalis tehuacanensis* Waterf., Rhodora 69: 203 (1967)

**Type:** Mexico, Puebla Tehuacán city dump, C. E. Smith Jr, Peterson and Tejeda 3992 (holotype: F barcode V0073015F!, isotype: US barcode 00027369!)

Perennial herb 22–27 cm tall with articulated capitate-glandular trichomes up to 1.5–2.0 mm long; leaves geminate; blades ovate or deltoid, 12–35 mm long, 12–35 mm wide, apex acute, base truncate, margin unevenly coarsely dentate or sinuate-dentate; flowers solitary on peduncles 4–6 mm long; calyx with lanceolate or ovate-lanceolate lobes 2.0–2.5 mm long, corolla rotate, yellow, immaculate, 10–11 mm wide; anthers yellow, 2.7–3.5 mm long; fruiting peduncles 8–10 mm long, fruiting calyx 10-costate, 15–18 mm long, 14–16 mm wide, berry 10–11 mm in diameter.

**Distribution and habitat:** Endemic to the Tehuacán area in the state of Puebla at 1800 m asl. Pue. (Figure 10B).

**Diagnostic characters:** Long, coarse, multicellular trichomes intermixed with shorter ones, many of the trichomes tipped with reddish-brown glands, coarsely and irregularly dentate leaves, and yellow anthers.

**Common names and uses:** None known.

**Phenology:** Collected with flowers in June.

**Conservation status:** CR.

**Representative specimens examined.** MEXICO. **Puebla**: Tehuacán city dump, C. E. Smith Jr, Peterson and Tejeda 3992 (F).

59. *Physalis vestita* Waterf., Rhodora 69: 326 (1967)

**Type:** Mexico, Sinaloa, vicinity of Mazatlán, J.N. Rose 13766 (holotype: US barcode 00027372!). (Figure 14H).

Erect perennial herb 15–65 cm tall from woody elongated roots; stems highly ramified, densely covered with branched trichomes throughout; leaves geminate, petioles 10–35 mm long; blades ovate, 25–40 mm long, 15–28 mm wide, apex acute, base truncate to oblique, margin entire or with two to three teeth per side, densely covered with gray branched trichomes; flowers solitary on peduncles 4–6 mm long; calyx with deltoid lobes 1 mm long, corolla rotate, yellow, 10–13 mm in diameter, with five purple maculations, corolla throat densely pubescent; anthers violet, 3–4 mm long; fruiting peduncle 7–13 mm long, fruiting calyx 5-angled, densely covered with branched trichomes, 15–22 mm long, 13–18 mm wide; mature berry green, 8–10 mm in diameter with numerous yellow seeds ca. 1 mm in diameter.

**Distribution and habitat:** Endemic to the rocky coast of Sinaloa, known only from three localities at sea level.

**Diagnostic characters:** A perennial plant densely covered with branched trichomes. However, the trichomes are forked, whereas the trichomes of *P. cinerascens* have several ramifications.

**Common names and uses:** None known.

**Phenology:** Bears flowers and fruits in June.

**Conservation status:** Not evaluated, but due to its restricted distribution, probably VU.

**Representative specimens examined.** MEXICO. **Sinaloa:** barra de Piaxtla, en el faro, *M. Martínez 9712* (QMEX).

60. *Physalis volubilis* Waterf., Rhodora 69: 229 (1967)

**Type:** Mexico, Michoacán, two miles above Tancitaro, Wm. C. Leavenworth 519 (holotype: GH 00077383!; isotypes: F barcode V0073021F!, MICH 138898!, NY 1109892!, MEXU!). (Figure 14G).

= *Physalis viridoflava* Waterf., Rhodora 69: 230 (1967). Type: Mexico, Jalisco, on route 110 at km 59–60, 20 miles due WSW of Jiquilpan, and several miles S of Mazamitla, D. P. Gregory, G. Eiton 99 (holotype: MICH barcode 1109893!, isotype: NY barcode 138896!).

Decumbent perennial herb up to 80 cm long, lignified at the base, rooting at the basal nodes, glabrous or with few eglandular, septate, appressed trichomes; leaves alternate throughout, petioles 1.2–7.1 cm long; blades ovate to broadly ovate, 1.0–5.0 cm long, 1.0–4.5 cm wide, apex obtuse to acute, base truncate to cordate, margin entire; flowers solitary on peduncle 3.5–8.5 cm long; calyx with narrow triangular lobes 4–7 mm long, corolla campanulate, rotate-reflected, yellow, 2.0–2.8 cm in diameter, with dark, purple-brown, compound maculations, corolla throat pubescent; anthers yellow, blue-tinged, 2.5–4.0 mm long; fruiting peduncles 1.7–6.0 cm long, fruiting calyx 5-angled, 2.0–2.6 cm long, 1.1–1.4 cm wide, mature berry 0.8–1.2 cm in diameter with yellow seeds ca. 1.5 mm in diameter.

**Distribution and habitat:** It develops mainly on slopes, margins, or clearings of pine–oak and cloud forests. It grows at an altitude range of 1,390–2,700 m. Mich., Jal. (Figure 5F).

**Diagnostic characters:** A prostrate habit, ovate leaves, a short rhizome, and long peduncles in flower and fruit.

**Common names and uses:** None known.

**Phenology:** Bears flowers and fruits from June to March.

**Conservation status:** LC.

**Representative specimens examined.** MEXICO. **Jalisco:** Concepción de Buenos Aires, 5 km al S de Buenos Aires, brecha a Ciudad Guzmán*, Villa 756* (IEB). **Michoacán:** Lado S de La Laguna Verde, zona geotérmica Los Azufres, municipio de Zinapécuaro, *M.J. Jasso 1094* (IEB).

61. *Physalis waterfallii* O.Vargas, M. Martínez & Dávila, Acta Bot. Mex. 48: 22 (1999)

**Type:** Mexico, Jalisco, municipio de Chiquilistlán, brecha Tapalpa-Chiquilistlán, A. Rodríguez 986 (holotype: IBUG!; isotypes: ENCB!, MEXU 445306!). (Figure 14I).

Perennial prostrate herb 60–100 cm long, lignified at the base, with eglandular trichomes ca. 3 mm long; leaves alternate at the base, soon geminate, petiole 1.3–4.0 cm long, blades ovate-lanceolate to broadly ovate, 3.5–11.0 cm long, 2–7 cm wide, apex acute to caudate, base truncate, oblique, narrowly decurrent, margin entire, rarely with one to three teeth, pubescence appressed; flowers solitary on peduncles 1.3–3.0 cm long; calyx with triangular lobes 0.6–1.0 cm long, corolla campanulate rotate, yellow, 1.4–2.0 cm long, 2–3 cm in diameter, with simple to compound reddish-brown maculations; anthers purple or blue, 3.0–3.5 mm long; fruiting peduncles 1.5–2.8 cm long, fruiting calyx 5-angled, 2.2–3.8 cm long, 1.2–2.6 mm wide; mature berry green to yellow ca. 1 cm in diameter with numerous yellow seeds ca. 2 mm in diameter.

**Distribution and habitat:** In pine–oak and cloud forests, in clearings and agricultural fields near the forest at 1,700–2,450 m. Jal., Mich., Qro. (Figure 13F).

**Diagnostic characters:** A long flower peduncle, the 5-angled fruiting calyx, and the long calyx lobes in flower and fruit up to 1.0 cm in length.

**Common names and uses:** None known.

**Phenology:** It flowers and bears fruit from July to November.

**Conservation status:** LC.

**Representative specimens examined.** MEXICO. **Jalisco:** Chiquilistlán, brecha Tapalpa-Chiqulistlán, *Rodríguez 986* (ENCB, IBUG, MEXU). **Michoacán:** municipio de Zinapécuaro, El Cerrito, 1 km al E de Jeráhuaro, *M. Jasso 199* (IEB, QMEX). **Querétaro:** entre San Joaquín y Corral Blanco, municipio de San Joaquín, *S. Zamudio 7945* (IEB).

## 9 Doubtful and excluded names

*Physalis ixocarpa* Brot. ex Hornem, Hort. Bot. Hafn. Suppl. 26 (1819).

**Type:** Mexico (BR 0000013069179!).

Annual herb up to 130 cm high, stems angled, puberulent, glabrescent; leaves alternate, petioles 1.8–4.1 cm long; blades linear to rhombic or ovate, 4.5–14 cm long, 2.3–5.1 cm wide, apex acute, base attenuate, sometimes oblique, margin dentate; flowers solitary on peduncle ca. 5 mm long; calyx with triangular lobes 1.7 mm long, corolla rotate, yellow, maculate, 0.7–1.2 cm in diameter; anthers green or blue-tinged, 1–2 mm long; fruiting peduncle 1 cm long, fruiting calyx 10-costate, 1.6 cm long, 3.4 cm wide, berry 1–2 cm in diameter.

Waterfall (1967) did not include this species in his treatment. Under our concept, the plant is similar to *P. philadelphica*, from which it differs in that the lectotype does not have convoluted anthers. Additionally, a capitate stigma was referred to *P. ixocarpa* (Fernandes, 1974), whereas *P. philadelphica* has a clavate stigma. Plants from Southern Mexico (Tabasco and Chiapas) can be referred to this taxon and were treated as such for Flora Mesoamericana (unpublished). The name *P. ixocarpa* is misapplied to cultivated husk tomato. According to molecular data, both species form a clade behaving as sisters (Deanna et al., 2018). We consider that *P. ixocarpa* could be synonymous with *P. philadelphica*; however, more extensive work is needed to address this issue.

*Physalis microphysa* A. Gray, Proc. Amer. Acad. Arts 21: 402 (1886).

**Type:** Mexico, near the city of Chihuahua: Santa Eulalia Mountains, limestone cliffs, C. G. Pringle 317 (holotype: GH 3286!, isotypes: BR not located, F barcode V0073004F!, MEXU-2762!).

= *Physalis campanulata* Brandegee, Univ. Calif. Publ. Bot. 4: 278 (1912). Type: Mexico, San Luis Potosí, C. A. Purpus 5313 (holotype: UC not located, isotypes: F not located, GH 00077343!, MEXU-27635, US barcode 00027317!).

Perennial herb from a woody base up to 60 cm tall; trichomes a mixture of glandular and eglandular trichomes; leaves alternate, petioles 5–12 mm long; blades reniform to ovate, 5–12 mm long, 10–23 mm wide, apex acute, base attenuate, margin dentate; flowers in pairs on peduncles 2 mm long; calyx with triangular lobes 2–3 mm long, corolla rotate, yellow, immaculate, 8–10 mm in diameter; fruiting calyx 10-costate, open apically, 9–12 mm long; fruit dry, dehiscent through irregular lines; seeds few, black.

The species is to be removed from *Physalis* because molecular evidence places it closer to *Chamaesaracha* and *Quincula* than to the rest of *Physalis* (Zamora-Tavares et al., 2016). Morphologically, it differs from *Physalis* in its open fruiting calyx, dry dehiscent fruit, and black seeds.

*Physalis muelleri* Waterf., Rhodora 69: 115 (1967).

**Type:** Mexico, Nuevo León, Diente Canyon, mountains near Monterrey, C. H. Mueller 129 (holotype: GH 77357!, isotype: F V0073005F!)

Perennial herb, 25–45 cm tall; trichomes short, up to 0.5 mm long, partly capitate-glandular; petioles 15–35 mm long, leaves ovate, 4–8 cm long, 2.8–5.5 cm wide; flowers solitary on peduncles 5–6 mm long; calyx 6–8 mm long and ca. 5 mm wide at the base of the ovate-deltoid or lanceolate lobes; corolla campanulate, yellow, 10–12 mm long, 12–15 mm wide with dark maculations; anthers yellow, 3.5–4 mm long; fruiting peduncles 10–15 mm long, fruiting calyx 10-costate, with short trichomes, partly capitate-glandular, 17–22 mm long and 15–18 m wide; mature berry 10–14 mm wide, seeds unknown.

The species is only known from the type specimen. The plant is a perennial with coarsely dentate leaves and glandular trichomes, but all those characters are also present in *P. hederifolia*. In his key, Waterfall (1967) distinguished *P. muelleri* from *P. hederifolia* because *P. muelleri* has a fruiting calyx almost filled by the berry and opened apically. However, the characters may be pressing artifacts.

*Physalis philippensis* Fernald, Proc. Amer. Acad. Arts 35: 568 (1900).

**Type:** Mexico: Oaxaca, Sierra de San Felipe C.G. Pringle 5621 (holotype: GH 00003292!, isotype: VT not located).

Perennial herb, 15–20 cm long, several-branched; vestiture of flattened, spreading, jointed trichomes of varying sizes; petioles 6–12 mm long, blades ovate to rhombic ovate to hastate-ovate, 15–20 mm long, 15–20 mm wide, apex obtuse to acute, base decurrent, margin usually with one to three irregularly shaped teeth, spreading-hairy to appressed-hairy on both sides; solitary flowers on peduncles 5–8 mm long; calyx lobes ovate-lanceolate to deltoid; corolla slightly lobed, 20–25 mm wide, with violet or purplish compound maculations, corolla throat tomentose; anthers bluish or violet, oblong, 3–4 mm long; fruit and seeds unknown.

The species was described over 120 years ago, but no recent collections fit the description. The lack of fruit further complicates the species concept.
